# Designing network based intervention strategies for epidemics of infectious diseases from edge based infection probability

**DOI:** 10.1038/s41598-025-33300-3

**Published:** 2026-01-06

**Authors:** Veronika Halász, Joacim Rocklöv

**Affiliations:** 1 Interdisciplinary Centre for Scientific Computing, Heidelberg University, Heidelberg, Germany; 2 Heidelberg Institute of Global Health, Heidelberg University, Heidelberg, Germany; 3 Department of Epidemiology and Global Health, Section of Sustainable Health, Umea University, Umea, Sweden

**Keywords:** Network topology, Probabilistic data networks

## Abstract

Epidemics underscore the critical role of human contact networks in shaping the spread of infectious diseases. Transmission varies depending on a range of factors, including virus characteristics, the type and duration of contact, and whether it occurs indoors or outdoors. However, not only does the probability of transmission differ, but the impact of each transmission event depends on the ability of a single event to spread the virus to new, previously unaffected, socially segmented groups in society. Effective policymaking should be guided by a nuanced understanding of how infections spread, ensuring that interventions are proportional to the risks they aim to address. In this study, we conducted a series of theoretical experiments on generated networks that are structurally similar to real social contact networks. Using models that distinguish between regular, repeated contacts and occasional, random, or transient contacts, we simulated fictitious epidemics on different sample graphs with varying contact restrictions and then compared their trajectories. Based on the observed differences, we identified the contact types whose restriction can effectively curb the epidemic. We find that it is particularly important to focus on relationships that form a bridge between clusters or communities and on contacts with particularly high transmission probability. By doing so, public health efforts can more effectively balance the dual goals of minimizing transmission and maintaining social and economic stability.

## Introduction

Pandemics present significant challenges to global public health systems, underscoring the interconnectedness of human populations. Understanding the role of networks in disease transmission is crucial for effective control measures^1–3^. Networks act as conduits for disease spread, with their structure influencing the speed and extent of transmission. Social networks, especially in densely populated areas or highly interactive communities, play a key role. Network science provides tools for measuring connectivity, centrality, and resilience, offering insights into disease dynamics^4,5^. Metrics like degree, betweenness, and closeness centrality^6,7^ help identify critical nodes and transmission pathways. By applying network analysis to epidemiological data, we can uncover transmission patterns, improve outbreak detection, and prioritize interventions. Network-based strategies leverage these insights to design targeted control measures, such as contact tracing and social distancing, to disrupt transmission.

Recent pandemics have shown that a pandemic is not just a health crisis but also presents societal and economic challenges. Countermeasures must be credible and supported by impact studies. Reducing encounters and social contacts is a primary method of controlling epidemics, but the effectiveness depends on how these reductions are implemented. Blanket restrictions, such as closing all non-essential businesses, can have severe economic consequences, particularly for small businesses and vulnerable workers, leading to job losses, closures, and long-term instability. Additionally, overly restrictive measures can harm mental health, increasing social isolation, anxiety, and depression. This can decrease public compliance and provoke unrest as people resist what they perceive as unreasonable rules.

Our goal is to establish theoretical foundations for effective action, even with partial knowledge of social networks. With incomplete understanding, it’s essential to rely on general principles to take effective action despite missing data.

This study investigates which strategy is most effective for limiting transmission and how to identify which connections should be restricted. In a network model^8^, this involves determining which edges to remove to reduce infection risk. While individuals with more neighbors may appear more exposed, the risk of infection varies depending on factors such as proximity, contact duration, and the broader network. The spread of infection depends not only on immediate neighbors but also on the entire network, including chains of infection within the same network component. Our models account for these factors using centrality measures.

While there are few studies directly addressing these questions, some provide comparable insights. Maier and Brockmann compare the impact of implemented restrictions with the epidemic’s potential course without measures^9^, and Ferguson et al. examine the combined effects of various interventions^10^. In the wake of COVID-19, studies like those by Gozzi et al.^11^ and Sneppen et al.^12^ explore the effects of lockdowns and the impact of contact categories (close, regular, random).

Related studies, while using different methods, often draw similar conclusions. Block et al. assess the effectiveness of various strategies against random edge removal and no-restriction scenarios. Their findings highlight the importance of node-to-node distances, which correlate with closeness centrality^13^. Gosak et al. also stress the effectiveness of limiting contacts outside communities^14^, while Nande et al. focus on within-household interactions and the probabilistic nature of transmission^15^. Recent studies have investigated epidemic spreading and control strategies in networked populations. Matamalas et al.^16^ and Liang et al.^17^ proposed link- and edge-based containment methods in specific network types, while Yang et al.^18^ and Yang et al.^19^ focused on traffic-driven spreading and local network bottlenecks, respectively. Brockmann and Helbing^20^ explored the hidden geometry of contagion, Gross and Havlin^21^ analyzed epidemic control in spatial modular networks, and more recent works, including Hedde-von Westernhagen et al.^22^, Maheshwari and Albert^23^, and Yucel et al.^24^, examined multi-layer centrality measures, social distancing effects, and the role of network centrality and socio-economic factors in slowing infection spread. While these studies provide valuable insights into network-driven epidemic processes, our work systematically compares multiple network types and thinning strategies, quantifying their impact on both network structure and epidemic dynamics, thereby extending and generalizing previous findings.

Our experiments follow these steps: We generate networks similar to real-world social networks, each containing 300 vertices, which balance pattern recognition and computational efficiency. Probabilities are randomly assigned to network edges based on different distributions and different expected values, indicating the likelihood of infection transmission between connected individuals. These probabilities are converted into edge weights to create a weighted network. We remove edges based on various strategies to sparsify the networks. We simulate epidemics on both original and sparse networks to assess how different restrictions affect transmission. For each parameter setting five different graphs were generated. For each graph five simulations were performed with each strategy. The same steps are performed on other sample network types.

## Methods

### Sample networks

We examine the cases of random and regular type contacts separately. That is why we use sample graphs that model the network of random contacts on the one hand, and those that model the regular ones on the other. There are also close contacts, mainly between people living in the same household. We refrain from examining them because such contacts cannot be limited in any way. Regular contacts refer to those with individuals having frequent face-to-face interactions. These could include close relatives who see each other regularly, close friends, persons who are in each other’s social circle (who meet regularly), colleagues and classmates, neighbors, members of the same club, religious group, or other local organizations, and regular service providers (like hairdressers or trainers, etc.). On the contrary, random and temporary contacts are typically characterized by their spontaneity and lack of ongoing interaction. These refer to infrequent, casual, or unplanned interactions. These contacts are typically not part of each other’s regular social circle and may include encounters in public spaces, like on the street, customers in a store, or on public transportation, brief encounters with service providers, event-based acquaintances, temporary colleagues, or travel encounters.

The networks of random contacts change much more dynamically than those of regular ones, precisely because of their irregularity. In real life, there are many social interactions that depend very little or not at all on people’s networks of relationships. In our model, we consider whether two people come into contact at such a public location as uniformly random, i.e., the probability of contact between any two individuals present is the same. The Erdős-Rényi model corresponds to this assumption.^25,26^

In the paper^27^, Barabási and Albert showed that networks of human relationships are typically scale-independent. Since regular contacts are generally based on such relationships, we model them here with so-called Barabási-Albert graphs. We can best interpret the networks of regular contacts within natural communities (for example, within a workplace, residential area, or village).

- **Erdős-Rényi graph:** The *erdos_renyi_graph(n, p)* is a binomial random graph with *n* nodes, where each of the possible edges is chosen with probability *p*.
- **Barabási-Albert graph:** The *barabasi_albert_graph(n, m)* gives a graph of *n* nodes is grown by attaching new nodes each with *m* edges that are preferentially attached to existing nodes with high degree. So, *n* is an integer denoting the number of nodes in the graph and *m* is another integer denoting the number of edges to attach from a new node to existing nodes.

The Barabási-Albert (BA) and Erdős-Rényi (ER) graphs are both models of random networks, but with distinct structural properties. In the appendix, you will find a table that itemizes the characteristics of these two network types.

Each time, our initial network is generated as either an Erdős-Rényi(*n*, *p*) graph or a Barabási-Albert(*n*, *m*) graph, each with different parameter pairs (*n*, *p*) and (*n*, *m*), respectively. We can generate such sample networks using Python’s NetworkX package with arbitrary parameter pairs, ensuring manageable network sizes.

### Weights

In graph theory, a path is a sequence of vertices where each adjacent pair is connected by an edge. In an unweighted graph, the path length refers to the number of edges in a path between two vertices. The shortest path is the path with the fewest edges between the two vertices. In a weighted graph, the path length is the sum of the weights of the edges in that path. The shortest path is the path where the sum of the edge weights is minimized. A shortest path between two vertices is not necessarily unique; there can be multiple paths with the same minimum length. That minimum length is also called the distance between the two end vertices.

In this case, we want the edge weights to express the probability of the infection spreading, while the weights of the paths should express the probability of spread between the two endpoints via the path. Specifically, it signifies the likelihood that the infection will traverse the given edge/path if one of its endpoints represents an infected individual. Since the joint probability is multiplicative (not additive), we cannot directly use probabilities as edge weights. We introduce a function transformation that allows us to calculate the distances and thus the centralities in the usual way.

This is $w(p)=\ln (\frac{1}{p})$, where *p* is a real number between 0 and 1, since the interpretation domain of the weight function *w*() is the value set of the probability function interpreted at the edges (*p*()). This transformation allows us to continue using addition in the shortest path algorithm. The property $\sum\nolimits_{{i \in E}} {w(p_{i} )} = w\left( {\prod\nolimits_{{i \in E}} {p_{i} } } \right)$ follows from the identity $\sum\nolimits_{i} {\ln \left( {\frac{1}{{p_{i} }}} \right) = \ln \left( {\frac{1}{{\prod\nolimits_{i} {p_{i} } }}} \right)}$ .

Indeed, the sum of the weights corresponding to the probabilities assigned to the edges within a set equals the weight corresponding to the product of the individual probabilities. We note that assigning a value of $p=0$ would result in an error, so it is not permitted. This limitation does not introduce a theoretical flaw, as $p=0$ would imply the absolute impossibility of transmission. However, practically, we always operate under the assumption of a minimal, albeit positive, probability, which can be arbitrarily small. If transmission can be definitively ruled out, it simply means there is no edge in the network through which the infection can propagate. Similarly, we also disallow the case of $p=1$, even though it wouldn’t result in a theoretical error like the former scenario. A value of $p=1$ would indicate a situation where transmission is guaranteed to occur. Essentially, this mirrors the concept of collapsing two endpoints of a representing edge into a single vertex within the graph. The weight $w(1)=\ln (\frac{1}{1})=0$ would also imply that, during the start of the Dijkstra algorithm from any vertex, the inclusion of this edge in a path does not increase its total weight, i.e., it is computationally very cost-effective.

Probabilities were assigned to edges based on different distributions. First, based on a uniform distribution in the range 0.05-0.15. On the other hand, based on an exponential distribution in the range (0,1) with different scale parameters (equal to the expected value on the full range of positive numbers). Since the probabilities can only take values between 0 and 1, therefore values greater than 1 were not allowed. Thirdly, we also tried the normal distribution on the range (0,1) with mean of 0.1 and standard deviation of 0.05. With this diversity, we aim to perform a thorough analysis and make observations on how the distribution type of transmission probabilities affects the spread in the network. For the exponential distribution, we also made a comparison between different parametric cases.

### Edge removal strategies

Edge centrality metrics measure the role of each edge in the flow within a network in different ways. The exact definitions can be found in the supplementary materials. Therefore, we will examine how appropriate it might be to decide on the edges to be eliminated (restrictive measures) based on these metrics. In total, we try five different strategies, each with several thinning levels, namely:

- Removing the top 20%/30%/40%/50%/80%/90% of all edges by their probability of infection transmission, without computing any centralities.
- Removing the top 20%/30%/40%/50%/80%/90% of all edges, prioritized by betweenness centrality, considering weights.
- Removing the top 20%/30%/40%/50%/80%/90% of all edges, prioritized by betweenness centrality, not considering their weights.
- Removing the top 20%/30%/40%/50%/80%/90% of all edges, prioritized by closeness centrality, considering their weights.
- Removing the top 20%/30%/40%/50%/80%/90% of all edges, prioritized by closeness centrality, not considering their weights.

### Simulations

The simulation is designed to model the spread of a disease over a network. The network structure is defined as either BA or ER graphs with 300 nodes that are generated. Each simulation starts from a randomly infectious site, all other nodes are set to a susceptible state. At each time step, infectious nodes attempt to influence their neighbors according to the probability determined by two factors: One is the original probability characteristic of the edge between them, and the other is the current immunity of the neighbor. Infection of a healthy individual can occur based on interactions with its neighbors, following a probabilistic rule. For each individual contact a random number on the range [0,1) is generated and compared to its actual infection probability. If the number is greater than the actual infection probability then the neighbor gets infected. In this case, the entry in the state vector representing this neighbor is updated to 1. The elements of the (binary) state vector and the vertices of the network have a mutually unique correspondence, with the infection for each element being denoted by 1.

During the simulation, the probability of infection transmission between two individuals corresponds to the probability assigned to the corresponding edge in the network. If there is no edge between the vertices representing two individuals, the infection cannot spread directly from one to the other during the simulation. At the onset of the simulation, the matrix containing the transmission probabilities is symmetric, meaning that for any two vertices “A” and “B” in the network, the probability of “A” infecting “B” is the same as the probability of “B” infecting “A”.

However, as the simulation progresses, we assume that individuals develop some level of immunity with each infection. In this scenario, we exclude fatalities, meaning all infected individuals return to the system in a healthy state. This places our model conceptually between classical SIS and SIR frameworks: individuals can be reinfected (as in SIS models), but their susceptibility is reduced after each recovery due to accumulated immunity (a feature reminiscent of SIR-type immunity dynamics). The immunity of individuals affects both their susceptibility to infection and their recovery. The greater the immunity of an individual, the more the probability of infection through any contact decreases. On the other hand, greater immunity also speeds up recovery. To model this, we use a vector whose dimension is equal to the number of individuals. At the start of the simulation, all elements of the immunity vector are equal to unity, i.e. exactly 1. Each time an individual recovers from an infection, the element representing its immunity is multiplied by an immunity parameter. Of course, the level of immunity developed depends on many factors, such as the immune system of the individual or the virus itself. These factors are generalised by assuming that each individual acquires, with each infection, an immunity that reduces the likelihood of infection by the same amount. At the same time, their probability of recovery at each time unit is correspondingly higher than during their most recent infection.

In our simulations, time is treated discretely rather than continuously. This means that we count the infected individuals per time unit. This unit of time corresponds to the average time during which an infected individual can infect another individual without yet infecting others. This period is different for each pathogen. During the simulation, we update the “status” vector at each time step. On the other hand, currently infected individuals can be returned to a healthy state. In our model, the probability of recovery follows a geometric distribution, i.e. the probability that an infected individual will recover in a given step is $\frac{1}{E}$, where the expected value of recovery time, denoted by *E*, is the average number of steps that a person spends in the diseased state without interruption. The length of a unit step is given by the latency time, so the expected recovery time in this (discrete) model is the ratio of the average disease duration to the latency time. In the simulation, a randomly generated number between 0 and 1 is compared to the *recovery parameter*; recovery occurs if this number exceeds the *recovery parameter*, so the actual probability of recovery in each step is ($1-$*recovery parameter*), following a geometric distribution with expected value *E*.

To assess the robustness of our results for the default parameter settings, we performed an expanded set of simulations with a substantially larger number of realizations. Specifically, we generated 250 simulations, 5 runs on each of 50 generated networks. This procedure was repeated for both network types and for all strategies and thinning levels, under the default parameter configuration (Immunity and the recovery parameters set to 1.5 and to 0.8, respectively, for BA(300, 20) and ER(300, 0.125)). For all other parameter combinations, the original 25 simulations per setting were used. Outcomes were averaged to obtain more accurate benchmark curves, allowing us to confirm that the trends observed with 25 runs are highly representative and that the differences between network types remain minimal. This approach provides high-confidence validation of the expected behaviour for the default parameters.

## Results

The behavior of the epidemic spread was broadly consistent across Barabási-Albert (BA) and Erdős-Rényi (ER) network models, provided that the number of edges was approximately equal. Under such conditions, both network types exhibited nearly identical epidemic dynamics.

In the absence of intervention (referred to as the ’Initial’ curve), a rapid escalation led to high infection peaks across all tested probability distributions. For exponential distributions, significant maximum infection levels have emerged even at transmission probabilities with low expected values (as low as 0.02). At an expected value of 0.05, maximum infection counts rarely fell below 200. In the range between 0.2 and 0.5, peak values showed little variation (see Table 1). Despite the seemingly low expected values, the exponential distribution yields a considerable number of high-probability edges, especially in denser networks, which facilitates extensive transmission.

**Table 1:** Average runoff parameters for unconstrained epidemics with different expected values of the edge transmission probabilities for exponential distributions (At each time step, the infection rate of a total of 25 runs at that time point was averaged. “peak” shows the maximum of the average curve obtained in this way, $t_{peak}$ shows when this maximum was reached, and $t_{half}$ indicates the number of steps after the maximum at which the number of infected individuals fell to half of the peak value. Here the immunity and the recovery parameters were set to 1.5 and to 0.8, respectively).

$\lambda$	Barabási-Albert(300, 20)			Erdős-Rényi(300, 0.125)		
	Peak	$t_{peak}$	$t_{half}$	Peak	$t_{peak}$	$t_{half}$
0.01	30.8	28	16	31.2	33	16
0.02	109.5	16	9	105.8	17	9
0.05	191.5	9	9	206.4	9	9
0.10	241.0	5	11	242.3	5	11
0.15	253.6	4	12	255.1	4	12
0.20	252.7	4	13	253.6	4	13
0.25	251.7	4	13	248.4	4	13
0.30	259.7	3	14	267.4	3	14
0.35	265.9	3	14	271.2	3	14
0.40	263.4	3	14	273.1	3	14
0.45	267.5	3	14	271.3	3	14
0.50	264.1	3	14	272.1	3	15

Just as reducing the degree of nodes has an impact on reducing the spread of an epidemic, the original degrees without restrictions have an impact on the spread of an epidemic without restrictions. To investigate this, we conducted several comparisons on networks with 300 vertices at different average degrees (see Table 2). We find that up to around 35, the epidemic spreads significantly faster as the average degree increases, while above that the increase in the avergage degree leads to a barely noticeable acceleration or expansion. In contrast, the decline phase, characterized by the half-life, does exhibit a deceleration, but not to the same magnitude as the acceleration observed during the growth phase.

**Table 2:** Average runoff parameters for unconstrained epidemics with different average degrees, controlled by the second parameter of the Barabási-Albert and the Erdős-Rényi networks, respectively. (At each time step, the infection rate of a total of 25 runs at that time point was averaged. “ peak” shows the maximum of the average curve obtained in this way, $t_{peak}$ shows when this maximum was reached, and $t_{half}$ indicates the number of steps after the maximum at which the number of infected individuals fell to half of the peak value. The the edge transmission probabilities followed an exponential distribution with expected value 0.1. The immunity parameter was set to 1.5 and the recovery parameter to 0.8).

Barabási-Albert(300,*m*)					Erdős-Rényi(300,*p*)				
*m*	Average degree	Peak	$t_{peak}$	$t_{half}$	*p*	Average degree	Peak	$t_{peak}$	$t_{half}$
5	$\approx$ 9.833	112.5	12	10	0.033	9.867	128.5	14	9
10	$\approx$ 19.33	196.0	8	9	0.065	19.435	208.4	8	9
20	$\approx$ 37.33	241.0	5	11	0.125	37.375	242.3	5	11
30	$\approx$ 54	246.8	4	12	0.18	53.82	238.6	4	13
50	$\approx$ 83.33	249.3	4	13	0.3	89.7	264.0	3	14

Across uniform, exponential, and normal distributions, the general shape of the ’Initial’ curve remained consistent when expected values of edge transmission probabilities were held constant.

Under uniform probability distributions, mitigation strategies showed limited effect at lower restriction levels. Even at a 50% restriction rate, the epidemic trajectory closely resembled the uncontrolled case. Only at 80–90% restriction did notable differences emerge; however, no clear, systematic advantage could be observed across all five strategies (see Table 3).

**Table 3:** Average peak values of the trajectories in the unrestricted and restricted cases due to the ’Weights’ strategy of varying degrees The networks were of type BA(300, 20), the immunity parameter was set to 1.5 and the recovery parameter to 0.8. The expected value for all three distributions was 0.1. (Exponential dist. with scale parameter $\lambda =0.1$, normal dist. with mean $\mu =0.1$ and standard deviation $\sigma =0.05$, and uniform dist. between 0.05 and 0.15).

Distribution	Initial	Weights					
		20%	30%	40%	50%	80%	90%
Exponential	241.0	200.8	146.9	125.1	70.1	1.0	1.0
Normal	239.0	224.2	200.7	172.4	147.7	10.9	1.0
Uniform	240.3	217.7	210.0	187.2	174.5	27.1	1.9

Remarkably, outbreaks could occur even at an expected value of transmission probability of 0.01, with simultaneous infection affecting up to 20–25% of the population in denser networks. Under favorable conditions—namely higher immunity (1.5) and faster recovery (0.6)—the ’Weights’ and ’Weighted Betweenness Centrality (B.C.)’ strategies were able to fully or nearly fully suppress outbreaks with as little as 20% restriction, while the remaining strategies required at least 40–50% restriction for comparable containment. In scenarios with lower immunity or slower recovery, maximum infection counts increased and differences in strategy effectiveness became more pronounced. At medium expected values of edge transmission probability, outbreaks persisted even with 40–50% restriction, though ’Weights’ and ’Weighted B.C.’ consistently outperformed the other strategies. This trend held true even at high transmission probabilities (e.g. with expected value 0.5).

The superiority of the ’Weights’ and ’Weighted B.C.’ strategies was especially evident under exponential distributions, which aligns with the fact that these strategies preferentially remove highly weighted (and thus high-risk) edges (see Table 4).

**Table 4:** Average peak values of the trajectories in the unrestricted and restricted cases due to the different strategies of 50% degree Network type and parameters of the simulations are the same as in Table 3.

Distribution	Initial	Weights	B.C.		C.C.	
			Weighted	Unweighted	Weighted	Unweighted
Exponential	241.0	70.1	74.0	175.2	190.0	187.6
Normal	239.0	147.7	160.5	182.6	183.9	187.9
Uniform	240.3	174.5	168.5	201.2	185.4	196.1

Higher immunity levels led to reduced peak infection values and shorter epidemic durations, while the time to peak remained relatively stable. Interestingly, the effectiveness of intervention strategies appeared largely independent of the immunity parameter. (See Table 5)

**Table 5:** Average peak values of the trajectories in the unrestricted and restricted cases due to the different strategies of 50% degree. The immunity parameter varied between 1.3 and 2.0. The networks were of type BA(300, 20), the recovery parameter was set to 0.8. The transmission probabilities of edges followed an exponential distribution with $\lambda =0.1$.

Immunity parameter	Initial	Weights	B.C.		C.C.	
			Weighted	Unweighted	Weighted	Unweighted
1.3	240.2	90.8	78.0	186.2	180.8	191.8
1.4	235.4	70.6	82.8	185.9	168.6	199.4
1.5	241.0	70.1	74.0	175.2	190.0	187.6
1.6	237.3	50.3	76.9	147.7	181.6	183.5
1.7	235.7	56.6	68.2	171.2	151.2	182.4
1.8	227.6	70.0	60.8	166.2	192.4	194.9
1.9	242.0	68.2	70.0	159.1	159.6	182.4
2.0	229.9	61.6	69.3	173.4	167.8	180.8

Faster average recovery rates were associated with both lower maximum infection counts and shorter epidemic lifespans. In such cases, the efficacy of ’Weights’ and ’Weighted B.C.’ was further enhanced (see Table 6). For the remaining strategies, significant benefits were only observed at high restriction levels (80–90%).

**Table 6:** Average peak values of the trajectories in the unrestricted and restricted cases due to the different strategies of 50% degree. The recovery parameter varied between 0.6 and 0.9. The networks were of type BA(300, 20), the immunity parameter was set to 1.5. The transmission probabilities of edges followed an exponential distribution with $\lambda =0.1$.

Recovery parameter	Initial	Weights	B.C.		C.C.	
			Weighted	Unweighted	Weighted	Unweighted
0.6	185.4	16.2	30.6	117.7	130.8	135.0
0.7	218.8	33.0	54.5	141.1	168.4	114.5
0.8	241.0	70.1	74.0	175.2	190.0	187.6
0.85	251.4	89.9	121.1	183.2	190.1	199.3
0.9	264.8	137.3	130.0	215.6	211.6	231.6

Most of the calculations were carried out on sample networks with 300 vertices. We were curious to see how well our observations would hold in larger networks. Therefore, we also performed some simulations on 600-vertices sample networks. When comparing the BA-type networks, we see that the maximum location and half-life of the ’Initial’ curves are roughly the same, while the maximum value is roughly twice that seen for the 300-vertices networks with the same second network parameters (see Table 7).

**Table 7:** Average runoff parameters for unconstrained epidemics on 300 and 600 vertices, respectively, with different average degrees controlled by the second parameter of the Barabási-Albert network. (At each time step, the infection rate of a total of 25 runs at that time point was averaged. “ peak” shows the maximum of the average curve obtained in this way, $t_{peak}$ shows when this maximum was reached, and $t_{half}$ indicates the number of steps after the maximum at which the number of infected individuals fell to half of the peak value. The the edge transmission probabilities followed an exponential distribution with expected value 0.1. The immunity parameter was set to 1.5 and the recovery parameter to 0.8).

*m*	Barabási-Albert(300,*m*)			Barabási-Albert(600,*m*)		
	Peak	$t_{peak}$	$t_{half}$	Peak	$t_{peak}$	$t_{half}$
5	112.5	12	10	211.4	12	11
10	196.0	8	9	355.1	9	9
20	241.0	5	11	479.1	6	11
30	246.8	4	12	473.9	4	13
50	249.3	4	13	503.2	4	13

We also made another comparison to see how the effects of the restrictions compare between networks of 300 and 600 vertices, respectively. To do this, we tried the ’Weights’ strategy on BA-type networks. Here again, we see that the maximum infection rate is roughly twice as high on 600-vertices networks compared to 300-vertices networks when the second parameter of the network type is the same (see Table 8).

**Table 8:** Average maximum infection numbers for constrained epidemics based on the ’Weights’ strategy on 300 and 600 vertices, respectively, with different average degrees controlled by the second parameter of the Barabási-Albert network. (Parameters of the simulations are the same as in Table 7).

*m*	Barabási-Albert(300,*m*)				Barabási-Albert(600,*m*)			
	Initial	20%	30%	50%	Initial	20%	30%	50
5	112.5	45.2	15.8	2.0	211.4	74.5	44.4	2.4
10	196.0	115.3	65.2	13.4	355.1	234.1	141.0	22.4
20	241.0	200.8	146.9	70.1	479.1	415.5	313.8	183.5
30	246.8	218.3	187.3	103.8	473.9	443.0	384.2	216.9
50	249.3	231.8	212.8	147.1	503.2	480.4	459.3	369.0

The trends reported above were confirmed by a larger set of simulations for the default parameter settings (see Methods), demonstrating that the observed behaviours are robust and representative. For all other parameter combinations, the results are based on the original 25 simulations per setting. The supplementary document contains the averaged simulation outcomes for all parametrizations. It offers the full set of simulation results in a systematic and transparent form.

**Figure 1:**
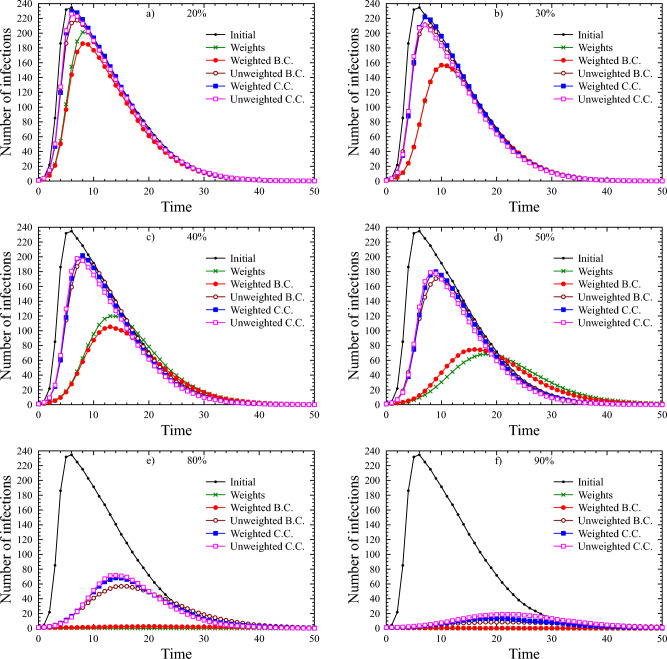
Simulations on Barabási-Albert(300, 20) networks. The six figures show average epidemic runs simulated on Barabási-Albert(300,20) sample networks. Here the immunity parameter was set to 1.5 and the recovery parameter to 0.8. In each figure, we present the trajectory of the case without restrictions, as well as the simulation of the execution of different strategies with a given restriction level (20%, 30%, 40%, 50%, 80% and 90%, respectively). The six graphics belong to the six restrictive measures.

**Figure 2:**
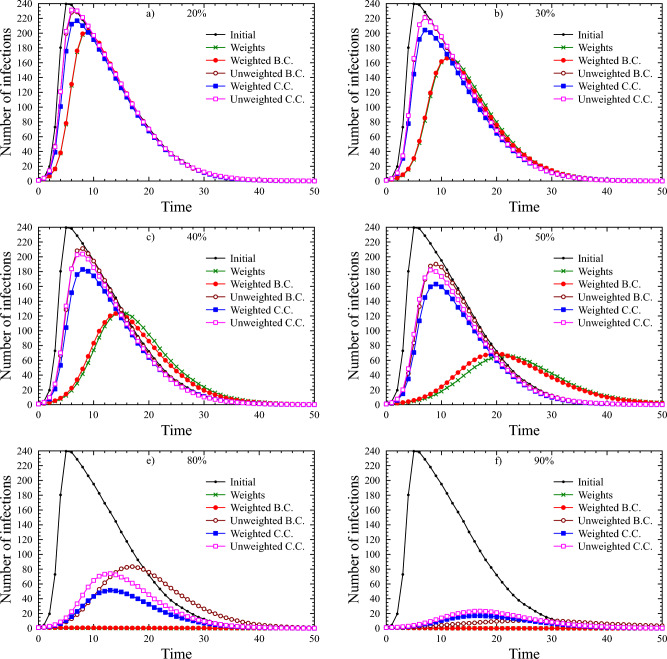
Simulations on Erdős-Rényi(300, 0.125) networks. The six figures show average epidemic runs simulated on Erdős-Rényi(300, 0.125) sample networks. Here the immunity parameter was set to 1.5 and the recovery parameter to 0.8. In each figure, we present the trajectory of the case without restrictions, as well as the simulation of the execution of different strategies with a given restriction level (20%, 30%, 40%, 50%, 80% and 90%, repectively). The six graphics belong to the six restrictive measures.

The figures below show full average trajectories. The transmission probabilities on edges followed exponential distribution with $\lambda =0.1$. The immunity factor was 1.5 and the recovery parameter was 0.8 in all cases. Averages are based on 5 simulations run on each of 50 different networks of the same type. The one type of network was Barabási-Albert(300, 20) (see Fig. 1), and the other type was Erdős-Rényi(300, 0.125) (see Fig. 2).

**Translating result into practice** It can be seen that focusing on contacts with a high transmission probability can lead to effective protection. These are encounters that occur in a confined airspace at a small physical distance. Reducing the number of these encounters and introducing appropriate protection (e.g. free-space positioning) is one effective strategy. Identification of high WBC edges is hardly possible without detailed knowledge of the relevant contact networks. In contrast, high BC contacts are easier to identify. Eliminating these and reducing the high transmission probabilities may be an effective alternative.

Although calculating exact edge betweenness centrality (EBC) is computationally intensive for large networks, previous results from the literature suggest that high-EBC edges can often be approximated based on common structural features. Such features include:

- Bridging edges between communities^28,29^: Critical for inter-community transmission; for instance, links between distinct social groups or regions in zoonotic spillover networks.
- Sparse connections or articulation points^30,31^: These often represent key transmission pathways in fragmented or rural populations.
- Edges on many shortest paths^32,33^: Typical in hierarchical systems, such as healthcare referral networks, where hubs play a central role in patient movement.
- Long-range edges^34,35^: Analogous to rapid long-distance transmission events, such as those observed in global air travel networks during pandemics.
- Central or bottleneck edges^36,37^: These correspond to high-contact points, such as densely populated markets or transportation hubs.

This also means that the elimination of contacts in very small communities does not play a significant role in the overall reduction, as these are typically of low betweenness centrality.

Taking these into account, we can also make some approximate estimates of the topology of the subnetworks remaining after thinning. Removing edges that act as bridges between different communities may lead to a more fragmented network. Social networks tend to be single connected graphs or a few large components. After the proposed eliminations, several smaller, weakly connected subgraphs may be formed. After removing high-betweenness edges, shortest paths become longer because the most efficient routes are cut off. In other words, the shortcuts disappear, and paths between communities take more hops. Interactions become more confined within small groups, meaning that the remaining links become locally denser. On the other hand, global reach decreases. Deleting high-betweenness edges disproportionately affects hubs, making the degree distribution more uniform. Especially in scale-free networks, a few high-degree nodes (the so-called hubs) control the connectivity. This could change significantly with the proposed restrictions, resulting in more but less centralized hubs. Last but not least, the alternative paths between nodes become fewer.

Our simulations confirm these qualitative expectations with quantitative evidence derived from the structural properties of the residual subnetworks. We computed the mean, median and standard deviation of the unweighted degrees, the weighted degrees and the clustering coefficients after thinning. The average weighted degree decreases most markedly under the two most efficient strategies. At the same time, the standard deviation of both the degrees and clustering coefficients shows a stronger decrease under less efficient strategies with milder restrictions, whereas it remains substantially higher when better-performing strategies are applied, indicating that these preserve more structural heterogeneity.

While the mean degree depends primarily on the overall extent of thinning, the median degree is strongly strategy dependent: it is significantly lower under the most effective strategies, reflecting the disproportionate removal of nodes that would otherwise remain moderately connected. This trend, however, does not persist in Erdős–R nyi networks at high thinning levels, where between 50% and 80% removal the behaviour reverses, favouring strategies based on closeness centrality. These results demonstrate that thinning strategies do not merely reduce connectivity but reshape the underlying topology in systematic and measurable ways.

## Discussion

This study explored strategies for mitigating epidemic spread by targeting specific network structures. Our findings provide theoretical frameworks adaptable to pathogens with diverse transmission dynamics, offering practical implications for epidemic control. It is important to note, however, that these findings arise from simulated scenarios using stylized network models; therefore, their applicability may vary across different epidemiological conditions and real-world transmission settings.

The results suggest that, on the one hand, restricting edges with high weighted betweenness centrality can be an effective strategy. On the other hand, targeting high-risk contacts with increased transmission potential yields similar results. While these patterns are robust within the simulated conditions, the relative effectiveness of the strategies may shift under alternative parameter sets, pathogen characteristics, or intervention environments. These strategies produce similar results to other strategies with a much higher level of restrictions, but with less socio-economic impact. This approach is especially relevant for diseases with heterogeneous transmission, like COVID-19, where superspreader events are key.

### Future outlook

While our simulations provide valuable insights, applying these strategies to real-world networks using empirical data would enhance the robustness of our findings. Future research could incorporate data from social, transportation, or healthcare networks to validate and refine our approach.

Our current model assumes static intervention strategies throughout the epidemic, which simplifies real-world dynamics. Future work could explore adaptive strategies, adjusting interventions based on the epidemic’s progression. Periodic adjustments to restricted edges at key phases of the epidemic could optimize outcomes and offer actionable insights for public health policies.

Another area for future research is the feasibility of edge removal. Not all contacts are modifiable—some, like those within healthcare or critical infrastructure, are harder to restrict than others. Categorizing edges based on both infection transmission potential and the social cost of restriction could make models more realistic and practical. This added complexity would enhance the applicability of the strategies to real-world situations.

Ultimately, advancing these research avenues will bridge the gap between theoretical models and practical public health interventions. A deeper understanding of network-based strategies will better equip policymakers to design interventions that balance effectiveness with social and economic considerations.

## Supplementary Information

Supplementary Information.

## Data Availability

All data generated or analysed during this study are included in this published article and its supplementary information files.

## References

[1] Pastor-Satorras, R. & Vespignani, A. Epidemic spreading in scale-free networks. *Phys. Rev. Lett.* **86**(14), 3200 (2001). 11290142 10.1103/PhysRevLett.86.3200

[2] Newman, M. E. Spread of epidemic disease on networks. *Phys. Rev. E* **66**(1), 016128 (2002). 10.1103/PhysRevE.66.016128 12241447

[3] Keeling, M. J. & Eames, K. T. Networks and epidemic models. *J. R. Soc. Interface* **2**(4), 295–307 (2005). 16849187 10.1098/rsif.2005.0051 PMC1578276

[4] Salathé, M. & Jones, J. H. Dynamics and control of diseases in networks with community structure. *PLoS Comput. Biol.* **6**(4), e1000736 (2010). 20386735 10.1371/journal.pcbi.1000736 PMC2851561

[5] Hufnagel, L., Brockmann, D. & Geisel, T. Forecast and control of epidemics in a globalized world. *Proc. Natl. Acad. Sci.* **101**(42), 15124–15129 (2004). 15477600 10.1073/pnas.0308344101 PMC524041

[6] Freeman, L. C. Centrality in social networks: Conceptual clarification. *Social Networks* **1**(3), 215–239 (1979).

[7] Borgatti, S. P. Centrality and network flow. *Social Networks* **27**(1), 55–71 (2005).

[8] Newman, M. E. Networks: An introduction. Oxford university press (2010)

[9] Maier, B. F. & Brockmann, D. Effective Containment Explains Subexponential Growth in Recent Confirmed COVID-19 Cases in China. *Science* **368**(6492), 742–746 (2020). 32269067 10.1126/science.abb4557 PMC7164388

[10] Ferguson, N. M., Cummings, D. A. T. & Fraser, C. Strategies for Containing an Emerging Influenza Pandemic in Southeast Asia. *Nature* **437**, 209–214 (2006). 10.1038/nature04017 16079797

[11] Gozzi, N., Bajardi, P. & Perra, N. Modelling the impact of social distancing and targeted vaccination on the spread of COVID-19 through a Real City-Scale Contact Network. *J. Theor. Biol.* **534**, 110973 (2021). 35039781 10.1093/comnet/cnab042 PMC8754788

[12] Sneppen, K., Nielsen, B. F., Taylor, R. J. & Simonsen, L. Overdispersion in COVID-19 increases the effectiveness of limiting nonrepetitive contacts for transmission control. *Proc. Natl. Acad. Sci.* **118**(14), e2016623118 (2021). 33741734 10.1073/pnas.2016623118 PMC8040586

[13] Block, P. et al. Social Network-Based Distancing Strategies to Flatten the COVID-19 Curve in a Post-Lockdown World. *Nat. Hum. Behav.* **4**, 588–596 (2020). 32499576 10.1038/s41562-020-0898-6

[14] Gosak, M., Duh, M., Markovič, R. & Perc, M. Community lockdowns in social networks hardly mitigate epidemic spreading. *New J. Phys.* **23**, 043039 (2021).

[15] Nande, A., Adlam, B., Sheen, J., Levy, M. Z. & Hill, A. L. Dynamics of COVID-19 under social distancing measures are driven by transmission network structure. *PLoS Comput. Biol.* **17**(2), e1008684. 10.1371/journal.pcbi.1008684 (2021). 33534808 10.1371/journal.pcbi.1008684 PMC7886148

[16] Matamalas, J. T. et al. Effective approach to epidemic containment using link equations in complex networks. *Sci. Adv.* **4**, eaau4212, (2018). 10.1126/sciadv.aau4212 PMC6281434 30525105

[17] Liang, G., Cui, X. & Zhu, P. An effective method for epidemic suppression by edge removing in complex network Front. *Phys.* **11**, 1164847 (2023).

[18] Yang, H.-X., Wu, Z.-X. & Wang, B.-H. Suppressing traffic-driven epidemic spreading by edge-removal strategies Phys. *Rev. E* **87**, 064801 (2013). 10.1103/PhysRevE.87.064801 23848813

[19] Yang, S., Senapati, P., Wang, D., Bauch, C. T. & Fountoulakis, K. Targeted pandemic containment through identifying local contact network bottlenecks. *PLoS Comput. Biol.* **17**, e1009351 (2021). 34460813 10.1371/journal.pcbi.1009351 PMC8432902

[20] Brockmann, D. & Helbing, D. The hidden geometry of complex, network-driven contagion phenomena. *Science* **342**, 1337–1342 (2013). 24337289 10.1126/science.1245200

[21] Gross, B. & Havlin, S. Epidemic spreading and control strategies in spatial modular network. *Appl. Netw. Sci.* **5**, 95 (2020). 33263074 10.1007/s41109-020-00337-4 PMC7689394

[22] Hedde-von Westernhagen, C., Bagheri, A. & Garcia-Bernardo, J. Predicting COVID-19 infections using multi-layer centrality measures in population-scale networks. *Appl. Netw. Sci.* **9**, 27 (2024).

[23] Maheshwari, P. & Albert, R. Network model and analysis of the spread of Covid-19 with social distancing. *Appl. Netw. Sci.* **5**, 100 (2020). 33392389 10.1007/s41109-020-00344-5 PMC7770744

[24] Y cel, S. G., Pereira, R. H. M., Peixoto, P. S., & Camargo, C. Q. Impact of network centrality and income on slowing infection spread after outbreaks. *Appl Netw Sci.* **8**(1), 16 (2023). 10.1007/s41109-023-00540-z PMC9951146 36855413

[25] Erdős, P. & Rényi, A. On the evolution of random graphs. *Publ. Math. Inst. Hung Acad. Sci.* **5**, 17–61 (1960).

[26] Bollobás, B. “Random Graphs.” 2nd ed. Cambridge University Press (2001).

[27] Barabási, A. L. & Albert, R. Emergence of scaling in random networks. *Science* **286**(5439), 509–512. 10.1126/science.286.5439.509 (1999). 10521342 10.1126/science.286.5439.509

[28] Girvan, M. & Newman, M. E. J. Community structure in social and biological networks. *Proc. Natl. Acad. Sci.* **99**(12), 7821–7826 (2002). 12060727 10.1073/pnas.122653799 PMC122977

[29] Newman, M. E. The structure and function of complex networks. *SIAM Rev.* **45**(2), 167–256 (2003).

[30] Holme, P., Kim, B. J., Yoon, C. N. & Han, S. K. Attack vulnerability of complex networks. *Phys. Rev. E* **65**(5), 056109 (2002). 10.1103/PhysRevE.65.056109 12059649

[31] Boccaletti, S., Latora, V., Moreno, Y., Chavez, M. & Hwang, D. U. Complex networks: Structure and dynamics. *Phys. Rep.* **424**(4–5), 175–308 (2006).

[32] Brandes, U. A faster algorithm for betweenness centrality. *J. Math. Sociol.* **25**(2), 163–177 (2001).

[33] Barthélemy, M. Betweenness centrality in large complex networks. *Eur. Phys. J. B* **38**(2), 163–168 (2004).

[34] Watts, D. J. & Strogatz, S. H. Collective dynamics of “small-world’’ networks. *Nature* **393**(6684), 440–442 (1998). 9623998 10.1038/30918

[35] Newman, M. E. & Watts, D. J. Renormalization group analysis of the small-world network model. *Phys. Lett. A* **263**(4–6), 341–346 (1999).

[36] Guimer, R., Mossa, S., Turtschi, A. & Amaral, L. A. N. The worldwide air transportation network: Anomalous centrality, community structure, and cities’ global roles. *Proc. Natl. Acad. Sci.* **102**(22), 7794–7799 (2005). 15911778 10.1073/pnas.0407994102 PMC1142352

[37] Freeman, L. C. A set of measures of centrality based on betweenness. *Sociometry* **40**(1), 35–41 (1977).

