# Supplementary material for: Designing network based intervention strategies for epidemics of infectious diseases from edge based infection probability
Source: Sci Rep. 2026 Jan 6;16:808. doi: 10.1038/s41598-025-33300-3 (PMC12780171; doi:10.1038/s41598-025-33300-3)
Supplement: Supplementary file 1 — Supplementary Information. [file 41598_2025_33300_MOESM1_ESM.pdf]

```
#-# parameters:
#-#  Model                    Barabasi
#-#  Number_of_nodes          300
#-#  Parameter_of_network     20
#-#  Distribution             exponential 0.01
#-#  Immunity_update          1.5 0.8
#-#  N_graph                  5
#-#  N_initial                1
#-#  Number_of_simulations    5
## Averages of all simulations (peak, t_peak, t_half)
## 0% 'Initial'          (30.8, 28, 16)
## 20% 'Weights'         (1.1, 1, 25)
## 20% 'Weighted B.C.'   (1.3, 57, 9)
## 20% 'Weighted C.C.'   (6.2, 41, 17)
## 20% 'Unweighted B.C.' (13.1, 26, 24)
## 20% 'Unweighted C.C.' (4.5, 45, 21)
## 30% 'Weights'         (1.0, 0, 6)
## 30% 'Weighted B.C.'   (1.6, 14, 15)
## 30% 'Weighted C.C.'   (4.4, 45, 19)
## 30% 'Unweighted B.C.' (7.7, 28, 21)
## 30% 'Unweighted C.C.' (2.0, 33, 48)
## 40% 'Weights'         (1.0, 0, 6)
## 40% 'Weighted B.C.'   (1.0, 0, 7)
## 40% 'Weighted C.C.'   (1.0, 0, 46)
## 40% 'Unweighted B.C.' (6.1, 29, 25)
## 40% 'Unweighted C.C.' (2.5, 46, 18)
## 50% 'Weights'         (1.0, 0, 5)
## 50% 'Weighted B.C.'   (1.0, 0, 7)
## 50% 'Weighted C.C.'   (1.1, 15, 13)
## 50% 'Unweighted B.C.' (1.2, 2, 40)
## 50% 'Unweighted C.C.' (1.0, 0, 23)
## 80% 'Weights'         (1.0, 0, 2)
## 80% 'Weighted B.C.'   (1.0, 0, 4)
## 80% 'Weighted C.C.'   (1.0, 0, 3)
## 80% 'Unweighted B.C.' (1.0, 0, 4)
## 80% 'Unweighted C.C.' (1.1, 1, 5)
## 90% 'Weights'         (1.0, 0, 4)
## 90% 'Weighted B.C.'   (1.0, 0, 2)
## 90% 'Weighted C.C.'   (1.0, 0, 3)
## 90% 'Unweighted B.C.' (1.0, 0, 4)
## 90% 'Unweighted C.C.' (1.0, 0, 5)

#-# parameters:
#-#  Model                    Erdos
#-#  Number_of_nodes          300
#-#  Parameter_of_network     0.125
#-#  Distribution             exponential 0.01
#-#  Immunity_update          1.5 0.8
#-#  N_graph                  5
#-#  N_initial                1
#-#  Number_of_simulations    5
## Averages of all simulations (peak, t_peak, t_half)
## 0% 'Initial'          (31.2, 33, 16)
## 20% 'Weights'         (1.2, 1, 25)
## 20% 'Weighted B.C.'   (1.1, 2, 14)
## 20% 'Weighted C.C.'   (4.2, 34, 27)
## 20% 'Unweighted B.C.' (16.3, 39, 18)
## 20% 'Unweighted C.C.' (7.8, 38, 31)
## 30% 'Weights'         (1.0, 0, 6)
## 30% 'Weighted B.C.'   (1.0, 0, 12)
## 30% 'Weighted C.C.'   (3.1, 13, 38)
## 30% 'Unweighted B.C.' (2.2, 34, 37)
## 30% 'Unweighted C.C.' (4.8, 38, 19)
## 40% 'Weights'         (1.0, 0, 7)
## 40% 'Weighted B.C.'   (1.0, 0, 7)
## 40% 'Weighted C.C.'   (1.6, 27, 39)
## 40% 'Unweighted B.C.' (2.2, 30, 27)
## 40% 'Unweighted C.C.' (2.1, 20, 14)
## 50% 'Weights'         (1.0, 0, 9)
## 50% 'Weighted B.C.'   (1.0, 0, 4)
## 50% 'Weighted C.C.'   (1.1, 1, 33)
```

```
## 50% 'Unweighted B.C.' (1.2, 1, 39)
## 50% 'Unweighted C.C.' (1.8, 21, 39)
## 80% 'Weights'         (1.0, 0, 2)
## 80% 'Weighted B.C.'   (1.0, 0, 4)
## 80% 'Weighted C.C.'   (1.0, 0, 4)
## 80% 'Unweighted B.C.' (1.0, 0, 4)
## 80% 'Unweighted C.C.' (1.0, 0, 9)
## 90% 'Weights'         (1.0, 0, 4)
## 90% 'Weighted B.C.'   (1.0, 0, 2)
## 90% 'Weighted C.C.'   (1.0, 0, 4)
## 90% 'Unweighted B.C.' (1.0, 0, 4)
## 90% 'Unweighted C.C.' (1.0, 0, 4)

#-# parameters:
#-#  Model                    Barabasi
#-#  Number_of_nodes          300
#-#  Parameter_of_network     20
#-#  Distribution             exponential 0.02
#-#  Immunity_update          1.5 0.8
#-#  N_graph                  5
#-#  N_initial                1
#-#  Number_of_simulations    5
## Averages of all simulations (peak, t_peak, t_half)
## 0% 'Initial'          (109.5, 16, 9)
## 20% 'Weights'         (22.3, 22, 16)
## 20% 'Weighted B.C.'   (26.5, 26, 16)
## 20% 'Weighted C.C.'   (78.6, 19, 12)
## 20% 'Unweighted B.C.' (71.4, 17, 12)
## 20% 'Unweighted C.C.' (61.2, 17, 12)
## 30% 'Weights'         (4.3, 29, 24)
## 30% 'Weighted B.C.'   (11.0, 33, 15)
## 30% 'Weighted C.C.'   (52.2, 22, 15)
## 30% 'Unweighted B.C.' (44.8, 20, 14)
## 30% 'Unweighted C.C.' (50.4, 22, 15)
## 40% 'Weights'         (2.5, 30, 10)
## 40% 'Weighted B.C.'   (1.2, 21, 79)
## 40% 'Weighted C.C.'   (30.5, 29, 14)
## 40% 'Unweighted B.C.' (48.2, 21, 12)
## 40% 'Unweighted C.C.' (41.8, 24, 14)
## 50% 'Weights'         (1.1, 7, 4)
## 50% 'Weighted B.C.'   (1.0, 0, 5)
## 50% 'Weighted C.C.'   (14.4, 33, 16)
## 50% 'Unweighted B.C.' (21.8, 28, 11)
## 50% 'Unweighted C.C.' (25.9, 30, 16)
## 80% 'Weights'         (1.0, 0, 6)
## 80% 'Weighted B.C.'   (1.0, 0, 4)
## 80% 'Weighted C.C.'   (1.2, 5, 13)
## 80% 'Unweighted B.C.' (1.1, 19, 22)
## 80% 'Unweighted C.C.' (1.0, 0, 14)
## 90% 'Weights'         (1.0, 0, 5)
## 90% 'Weighted B.C.'   (1.0, 0, 3)
## 90% 'Weighted C.C.'   (1.0, 0, 6)
## 90% 'Unweighted B.C.' (1.0, 0, 7)
## 90% 'Unweighted C.C.' (1.0, 0, 5)

#-# parameters:
#-#  Model                    Erdos
#-#  Number_of_nodes          300
#-#  Parameter_of_network     0.125
#-#  Distribution             exponential 0.02
#-#  Immunity_update          1.5 0.8
#-#  N_graph                  5
#-#  N_initial                1
#-#  Number_of_simulations    5
## Averages of all simulations (peak, t_peak, t_half)
## 0% 'Initial'          (105.8, 17, 9)
## 20% 'Weights'         (25.0, 31, 18)
## 20% 'Weighted B.C.'   (19.3, 36, 11)
## 20% 'Weighted C.C.'   (74.4, 22, 12)
## 20% 'Unweighted B.C.' (52.3, 23, 9)
## 20% 'Unweighted C.C.' (78.4, 19, 11)
## 30% 'Weights'         (1.8, 7, 39)
## 30% 'Weighted B.C.'   (2.8, 19, 26)
```

```
## 30% 'Weighted C.C.'   (44.0, 23, 11)
## 30% 'Unweighted B.C.' (53.4, 22, 12)
## 30% 'Unweighted C.C.' (54.4, 25, 10)
## 40% 'Weights'         (1.4, 4, 25)
## 40% 'Weighted B.C.'   (1.0, 0, 22)
## 40% 'Weighted C.C.'   (30.5, 26, 15)
## 40% 'Unweighted B.C.' (35.5, 24, 12)
## 40% 'Unweighted C.C.' (36.4, 24, 17)
## 50% 'Weights'         (1.0, 0, 9)
## 50% 'Weighted B.C.'   (1.1, 1, 11)
## 50% 'Weighted C.C.'   (16.5, 27, 19)
## 50% 'Unweighted B.C.' (21.6, 30, 14)
## 50% 'Unweighted C.C.' (35.1, 30, 12)
## 80% 'Weights'         (1.0, 0, 3)
## 80% 'Weighted B.C.'   (1.0, 0, 3)
## 80% 'Weighted C.C.'   (1.0, 0, 5)
## 80% 'Unweighted B.C.' (1.0, 0, 19)
## 80% 'Unweighted C.C.' (1.1, 28, 19)
## 90% 'Weights'         (1.0, 0, 4)
## 90% 'Weighted B.C.'   (1.0, 0, 2)
## 90% 'Weighted C.C.'   (1.0, 0, 3)
## 90% 'Unweighted B.C.' (1.0, 0, 6)
## 90% 'Unweighted C.C.' (1.0, 0, 5)

#-# parameters:
#-#  Model                   Barabasi
#-#  Number_of_nodes         300
#-#  Parameter_of_network    20
#-#  Distribution            exponential 0.05
#-#  Immunity_update         1.5 0.8
#-#  N_graph                 5
#-#  N_initial               1
#-#  Number_of_simulations   5
## Averages of all simulations (peak, t_peak, t_half)
## 0% 'Initial'          (191.5, 9, 9)
## 20% 'Weights'         (119.0, 13, 9)
## 20% 'Weighted B.C.'   (123.0, 14, 9)
## 20% 'Weighted C.C.'   (182.5, 10, 9)
## 20% 'Unweighted B.C.' (187.7, 9, 9)
## 20% 'Unweighted C.C.' (183.6, 10, 8)
## 30% 'Weights'         (83.6, 16, 12)
## 30% 'Weighted B.C.'   (84.0, 16, 11)
## 30% 'Weighted C.C.'   (168.5, 12, 8)
## 30% 'Unweighted B.C.' (151.8, 11, 8)
## 30% 'Unweighted C.C.' (149.7, 10, 9)
## 40% 'Weights'         (46.4, 23, 14)
## 40% 'Weighted B.C.'   (42.6, 20, 12)
## 40% 'Weighted C.C.'   (142.4, 12, 9)
## 40% 'Unweighted B.C.' (138.8, 13, 9)
## 40% 'Unweighted C.C.' (149.6, 12, 9)
## 50% 'Weights'         (9.0, 29, 24)
## 50% 'Weighted B.C.'   (20.9, 28, 16)
## 50% 'Weighted C.C.'   (98.4, 14, 9)
## 50% 'Unweighted B.C.' (105.0, 13, 10)
## 50% 'Unweighted C.C.' (98.3, 14, 9)
## 80% 'Weights'         (1.0, 0, 3)
## 80% 'Weighted B.C.'   (1.0, 0, 8)
## 80% 'Weighted C.C.'   (19.7, 28, 13)
## 80% 'Unweighted B.C.' (10.8, 27, 17)
## 80% 'Unweighted C.C.' (20.2, 25, 14)
## 90% 'Weights'         (1.0, 0, 2)
## 90% 'Weighted B.C.'   (1.0, 0, 3)
## 90% 'Weighted C.C.'   (2.0, 21, 36)
## 90% 'Unweighted B.C.' (1.0, 0, 11)
## 90% 'Unweighted C.C.' (1.8, 21, 6)

#-# parameters:
#-#  Model                   Erdos
#-#  Number_of_nodes         300
#-#  Parameter_of_network    0.125
#-#  Distribution            exponential 0.05
#-#  Immunity_update         1.5 0.8
#-#  N_graph                 5
```

```
#-#  N_initial              1
#-#  Number_of_simulations   5
## Averages of all simulations (peak, t_peak, t_half)
## 0% 'Initial'          (206.4, 9, 9)
## 20% 'Weights'         (129.4, 14, 10)
## 20% 'Weighted B.C.'   (125.8, 16, 8)
## 20% 'Weighted C.C.'   (174.1, 11, 9)
## 20% 'Unweighted B.C.' (192.4, 10, 9)
## 20% 'Unweighted C.C.' (166.4, 10, 8)
## 30% 'Weights'         (103.2, 20, 9)
## 30% 'Weighted B.C.'   (79.7, 19, 10)
## 30% 'Weighted C.C.'   (140.2, 11, 9)
## 30% 'Unweighted B.C.' (174.4, 11, 9)
## 30% 'Unweighted C.C.' (164.8, 11, 8)
## 40% 'Weights'         (24.1, 28, 13)
## 40% 'Weighted B.C.'   (35.5, 26, 16)
## 40% 'Weighted C.C.'   (134.7, 13, 9)
## 40% 'Unweighted B.C.' (111.4, 13, 9)
## 40% 'Unweighted C.C.' (153.1, 12, 9)
## 50% 'Weights'         (4.2, 50, 17)
## 50% 'Weighted B.C.'   (7.0, 32, 23)
## 50% 'Weighted C.C.'   (98.7, 14, 9)
## 50% 'Unweighted B.C.' (132.6, 14, 9)
## 50% 'Unweighted C.C.' (134.3, 12, 9)
## 80% 'Weights'         (1.0, 0, 4)
## 80% 'Weighted B.C.'   (1.0, 0, 4)
## 80% 'Weighted C.C.'   (20.6, 22, 14)
## 80% 'Unweighted B.C.' (22.2, 28, 15)
## 80% 'Unweighted C.C.' (32.3, 19, 14)
## 90% 'Weights'         (1.0, 0, 2)
## 90% 'Weighted B.C.'   (1.0, 0, 4)
## 90% 'Weighted C.C.'   (2.7, 34, 24)
## 90% 'Unweighted B.C.' (1.6, 6, 6)
## 90% 'Unweighted C.C.' (3.7, 34, 12)

#-# parameters:
#-#  Model                  Barabasi
#-#  Number_of_nodes        300
#-#  Parameter_of_network    20
#-#  Distribution           exponential 0.1
#-#  Immunity_update        1.5 0.8
#-#  N_graph                5
#-#  N_initial              1
#-#  Number_of_simulations   5
## Averages of all simulations (peak, t_peak, t_half)
## 0% 'Initial'          (241.0, 5, 11)
## 20% 'Weights'         (200.8, 9, 9)
## 20% 'Weighted B.C.'   (186.0, 9, 9)
## 20% 'Weighted C.C.'   (228.7, 6, 10)
## 20% 'Unweighted B.C.' (225.7, 7, 10)
## 20% 'Unweighted C.C.' (227.8, 7, 10)
## 30% 'Weights'         (146.9, 10, 9)
## 30% 'Weighted B.C.'   (149.6, 11, 8)
## 30% 'Weighted C.C.'   (214.2, 7, 10)
## 30% 'Unweighted B.C.' (224.6, 7, 9)
## 30% 'Unweighted C.C.' (210.5, 7, 10)
## 40% 'Weights'         (125.1, 13, 10)
## 40% 'Weighted B.C.'   (121.9, 13, 9)
## 40% 'Weighted C.C.'   (213.3, 8, 9)
## 40% 'Unweighted B.C.' (185.2, 8, 9)
## 40% 'Unweighted C.C.' (209.8, 8, 9)
## 50% 'Weights'         (70.1, 18, 10)
## 50% 'Weighted B.C.'   (74.0, 15, 12)
## 50% 'Weighted C.C.'   (190.0, 9, 9)
## 50% 'Unweighted B.C.' (175.2, 9, 9)
## 50% 'Unweighted C.C.' (187.6, 8, 9)
## 80% 'Weights'         (1.0, 0, 3)
## 80% 'Weighted B.C.'   (4.1, 22, 16)
## 80% 'Weighted C.C.'   (66.0, 15, 10)
## 80% 'Unweighted B.C.' (80.8, 16, 11)
## 80% 'Unweighted C.C.' (85.7, 14, 10)
## 90% 'Weights'         (1.0, 0, 3)
## 90% 'Weighted B.C.'   (1.0, 0, 17)
```

```
## 90% 'Weighted C.C.'   (15.5, 18, 12)
## 90% 'Unweighted B.C.' (16.4, 15, 14)
## 90% 'Unweighted C.C.' (21.6, 22, 11)

#-# parameters:
#-#  Model                  Erdos
#-#  Number_of_nodes        300
#-#  Parameter_of_network    0.125
#-#  Distribution           exponential 0.1
#-#  Immunity_update        1.5 0.8
#-#  N_graph                5
#-#  N_initial              1
#-#  Number_of_simulations   5
## Averages of all simulations (peak, t_peak, t_half)
## 0% 'Initial'          (242.3, 5, 11)
## 20% 'Weights'         (200.4, 9, 9)
## 20% 'Weighted B.C.'   (210.5, 9, 9)
## 20% 'Weighted C.C.'   (225.4, 7, 10)
## 20% 'Unweighted B.C.' (226.0, 6, 10)
## 20% 'Unweighted C.C.' (220.0, 6, 11)
## 30% 'Weights'         (153.0, 12, 8)
## 30% 'Weighted B.C.'   (143.9, 12, 8)
## 30% 'Weighted C.C.'   (215.0, 7, 10)
## 30% 'Unweighted B.C.' (228.0, 7, 10)
## 30% 'Unweighted C.C.' (210.0, 7, 9)
## 40% 'Weights'         (127.6, 15, 9)
## 40% 'Weighted B.C.'   (136.4, 16, 8)
## 40% 'Weighted C.C.'   (176.1, 8, 9)
## 40% 'Unweighted B.C.' (219.8, 8, 9)
## 40% 'Unweighted C.C.' (206.6, 8, 9)
## 50% 'Weights'         (64.3, 23, 10)
## 50% 'Weighted B.C.'   (75.0, 22, 10)
## 50% 'Weighted C.C.'   (143.2, 10, 8)
## 50% 'Unweighted B.C.' (199.8, 9, 9)
## 50% 'Unweighted C.C.' (150.0, 9, 10)
## 80% 'Weights'         (1.0, 0, 5)
## 80% 'Weighted B.C.'   (1.0, 0, 10)
## 80% 'Weighted C.C.'   (53.4, 14, 9)
## 80% 'Unweighted B.C.' (65.8, 18, 10)
## 80% 'Unweighted C.C.' (82.6, 11, 9)
## 90% 'Weights'         (1.0, 0, 2)
## 90% 'Weighted B.C.'   (1.0, 0, 4)
## 90% 'Weighted C.C.'   (13.0, 19, 10)
## 90% 'Unweighted B.C.' (9.2, 32, 27)
## 90% 'Unweighted C.C.' (25.2, 19, 8)

#-# parameters:
#-#  Model                  Barabasi
#-#  Number_of_nodes        300
#-#  Parameter_of_network    20
#-#  Distribution           exponential 0.15
#-#  Immunity_update        1.5 0.8
#-#  N_graph                5
#-#  N_initial              1
#-#  Number_of_simulations   5
## Averages of all simulations (peak, t_peak, t_half)
## 0% 'Initial'          (253.6, 4, 12)
## 20% 'Weights'         (220.2, 7, 10)
## 20% 'Weighted B.C.'   (218.6, 7, 9)
## 20% 'Weighted C.C.'   (235.5, 5, 11)
## 20% 'Unweighted B.C.' (230.8, 6, 10)
## 20% 'Unweighted C.C.' (244.7, 5, 11)
## 30% 'Weights'         (194.2, 9, 9)
## 30% 'Weighted B.C.'   (186.1, 9, 9)
## 30% 'Weighted C.C.'   (238.1, 6, 10)
## 30% 'Unweighted B.C.' (226.7, 6, 10)
## 30% 'Unweighted C.C.' (219.6, 5, 11)
## 40% 'Weights'         (148.1, 10, 10)
## 40% 'Weighted B.C.'   (145.0, 9, 10)
## 40% 'Weighted C.C.'   (221.5, 6, 10)
## 40% 'Unweighted B.C.' (229.3, 7, 10)
## 40% 'Unweighted C.C.' (209.6, 6, 11)
## 50% 'Weights'         (124.6, 13, 9)
```

```
## 50% 'Weighted B.C.'   (116.9, 11, 9)
## 50% 'Weighted C.C.'   (206.1, 7, 10)
## 50% 'Unweighted B.C.' (198.4, 8, 10)
## 50% 'Unweighted C.C.' (190.8, 6, 10)
## 80% 'Weights'         (1.0, 0, 4)
## 80% 'Weighted B.C.'   (14.0, 23, 10)
## 80% 'Weighted C.C.'   (116.2, 10, 10)
## 80% 'Unweighted B.C.' (92.1, 12, 10)
## 80% 'Unweighted C.C.' (100.9, 10, 9)
## 90% 'Weights'         (1.0, 0, 3)
## 90% 'Weighted B.C.'   (1.0, 0, 48)
## 90% 'Weighted C.C.'   (36.9, 14, 11)
## 90% 'Unweighted B.C.' (25.8, 19, 11)
## 90% 'Unweighted C.C.' (37.2, 17, 9)

#-# parameters:
#-#  Model                    Erdos
#-#  Number_of_nodes          300
#-#  Parameter_of_network     0.125
#-#  Distribution             exponential 0.15
#-#  Immunity_update          1.5 0.8
#-#  N_graph                  5
#-#  N_initial                1
#-#  Number_of_simulations    5
## Averages of all simulations (peak, t_peak, t_half)
## 0% 'Initial'          (255.1, 4, 12)
## 20% 'Weights'         (228.7, 7, 10)
## 20% 'Weighted B.C.'   (222.1, 6, 11)
## 20% 'Weighted C.C.'   (237.3, 6, 10)
## 20% 'Unweighted B.C.' (238.1, 5, 11)
## 20% 'Unweighted C.C.' (234.8, 5, 11)
## 30% 'Weights'         (204.4, 9, 9)
## 30% 'Weighted B.C.'   (211.9, 9, 9)
## 30% 'Weighted C.C.'   (226.8, 6, 11)
## 30% 'Unweighted B.C.' (221.6, 5, 11)
## 30% 'Unweighted C.C.' (232.6, 5, 11)
## 40% 'Weights'         (188.4, 11, 8)
## 40% 'Weighted B.C.'   (172.6, 11, 9)
## 40% 'Weighted C.C.'   (209.4, 7, 10)
## 40% 'Unweighted B.C.' (220.8, 6, 11)
## 40% 'Unweighted C.C.' (218.7, 7, 10)
## 50% 'Weights'         (134.9, 15, 9)
## 50% 'Weighted B.C.'   (110.6, 14, 9)
## 50% 'Weighted C.C.'   (188.9, 7, 10)
## 50% 'Unweighted B.C.' (225.3, 7, 10)
## 50% 'Unweighted C.C.' (182.8, 7, 10)
## 80% 'Weights'         (1.0, 0, 4)
## 80% 'Weighted B.C.'   (16.0, 20, 14)
## 80% 'Weighted C.C.'   (74.4, 9, 11)
## 80% 'Unweighted B.C.' (124.8, 13, 9)
## 80% 'Unweighted C.C.' (94.4, 11, 8)
## 90% 'Weights'         (1.0, 0, 4)
## 90% 'Weighted B.C.'   (5.6, 22, 25)
## 90% 'Weighted C.C.'   (24.3, 13, 9)
## 90% 'Unweighted B.C.' (37.7, 21, 13)
## 90% 'Unweighted C.C.' (31.6, 12, 9)

#-# parameters:
#-#  Model                    Barabasi
#-#  Number_of_nodes          300
#-#  Parameter_of_network     20
#-#  Distribution             exponential 0.2
#-#  Immunity_update          1.5 0.8
#-#  N_graph                  5
#-#  N_initial                1
#-#  Number_of_simulations    5
## Averages of all simulations (peak, t_peak, t_half)
## 0% 'Initial'          (252.7, 4, 13)
## 20% 'Weights'         (227.8, 6, 10)
## 20% 'Weighted B.C.'   (234.4, 5, 11)
## 20% 'Weighted C.C.'   (247.0, 5, 11)
## 20% 'Unweighted B.C.' (242.5, 4, 12)
## 20% 'Unweighted C.C.' (240.0, 4, 13)
```

```
## 30% 'Weights'         (215.1, 8, 9)
## 30% 'Weighted B.C.'   (169.5, 8, 10)
## 30% 'Weighted C.C.'   (243.0, 5, 12)
## 30% 'Unweighted B.C.' (242.4, 5, 11)
## 30% 'Unweighted C.C.' (240.2, 5, 12)
## 40% 'Weights'         (177.6, 9, 9)
## 40% 'Weighted B.C.'   (200.6, 8, 9)
## 40% 'Weighted C.C.'   (237.9, 5, 11)
## 40% 'Unweighted B.C.' (234.6, 6, 11)
## 40% 'Unweighted C.C.' (221.2, 6, 11)
## 50% 'Weights'         (118.8, 12, 9)
## 50% 'Weighted B.C.'   (141.6, 8, 10)
## 50% 'Weighted C.C.'   (203.4, 6, 11)
## 50% 'Unweighted B.C.' (228.4, 7, 10)
## 50% 'Unweighted C.C.' (208.0, 6, 11)
## 80% 'Weights'         (1.0, 0, 8)
## 80% 'Weighted B.C.'   (49.2, 15, 8)
## 80% 'Weighted C.C.'   (99.9, 9, 10)
## 80% 'Unweighted B.C.' (78.0, 13, 9)
## 80% 'Unweighted C.C.' (117.6, 9, 9)
## 90% 'Weights'         (1.0, 0, 4)
## 90% 'Weighted B.C.'   (8.0, 25, 14)
## 90% 'Weighted C.C.'   (36.4, 12, 8)
## 90% 'Unweighted B.C.' (34.6, 17, 12)
## 90% 'Unweighted C.C.' (39.2, 14, 9)

#-# parameters:
#-#  Model                   Erdos
#-#  Number_of_nodes         300
#-#  Parameter_of_network    0.125
#-#  Distribution            exponential 0.2
#-#  Immunity_update         1.5 0.8
#-#  N_graph                 5
#-#  N_initial               1
#-#  Number_of_simulations   5
## Averages of all simulations (peak, t_peak, t_half)
## 0% 'Initial'          (253.6, 4, 13)
## 20% 'Weights'         (243.6, 6, 11)
## 20% 'Weighted B.C.'   (239.8, 6, 11)
## 20% 'Weighted C.C.'   (233.4, 5, 12)
## 20% 'Unweighted B.C.' (253.3, 4, 13)
## 20% 'Unweighted C.C.' (248.5, 4, 13)
## 30% 'Weights'         (225.8, 7, 10)
## 30% 'Weighted B.C.'   (225.6, 7, 10)
## 30% 'Weighted C.C.'   (221.0, 5, 12)
## 30% 'Unweighted B.C.' (242.2, 5, 12)
## 30% 'Unweighted C.C.' (237.8, 4, 13)
## 40% 'Weights'         (183.2, 9, 9)
## 40% 'Weighted B.C.'   (207.1, 8, 9)
## 40% 'Weighted C.C.'   (223.9, 6, 11)
## 40% 'Unweighted B.C.' (241.2, 6, 11)
## 40% 'Unweighted C.C.' (237.8, 5, 12)
## 50% 'Weights'         (151.0, 13, 9)
## 50% 'Weighted B.C.'   (126.0, 9, 9)
## 50% 'Weighted C.C.'   (187.5, 6, 11)
## 50% 'Unweighted B.C.' (233.8, 6, 11)
## 50% 'Unweighted C.C.' (229.8, 5, 12)
## 80% 'Weights'         (1.0, 0, 22)
## 80% 'Weighted B.C.'   (54.8, 12, 9)
## 80% 'Weighted C.C.'   (81.8, 8, 9)
## 80% 'Unweighted B.C.' (140.2, 11, 10)
## 80% 'Unweighted C.C.' (112.6, 8, 11)
## 90% 'Weights'         (1.0, 0, 3)
## 90% 'Weighted B.C.'   (12.8, 14, 9)
## 90% 'Weighted C.C.'   (47.8, 10, 9)
## 90% 'Unweighted B.C.' (56.4, 17, 11)
## 90% 'Unweighted C.C.' (51.3, 11, 9)

#-# parameters:
#-#  Model                   Barabasi
#-#  Number_of_nodes         300
#-#  Parameter_of_network    20
#-#  Distribution            exponential 0.25
```

```
#-#  Immunity_update         1.5 0.8
#-#  N_graph                 5
#-#  N_initial               1
#-#  Number_of_simulations   5
## Averages of all simulations (peak, t_peak, t_half)
## 0% 'Initial'          (251.7, 4, 13)
## 20% 'Weights'         (245.8, 5, 11)
## 20% 'Weighted B.C.'   (240.5, 5, 11)
## 20% 'Weighted C.C.'   (254.6, 4, 13)
## 20% 'Unweighted B.C.' (247.8, 4, 13)
## 20% 'Unweighted C.C.' (250.4, 4, 13)
## 30% 'Weights'         (225.1, 6, 11)
## 30% 'Weighted B.C.'   (218.0, 6, 10)
## 30% 'Weighted C.C.'   (239.4, 4, 13)
## 30% 'Unweighted B.C.' (243.1, 4, 13)
## 30% 'Unweighted C.C.' (240.7, 4, 13)
## 40% 'Weights'         (192.1, 8, 9)
## 40% 'Weighted B.C.'   (210.3, 6, 10)
## 40% 'Weighted C.C.'   (232.3, 5, 12)
## 40% 'Unweighted B.C.' (234.8, 6, 11)
## 40% 'Unweighted C.C.' (218.8, 5, 12)
## 50% 'Weights'         (158.8, 9, 9)
## 50% 'Weighted B.C.'   (161.4, 6, 10)
## 50% 'Weighted C.C.'   (217.8, 5, 12)
## 50% 'Unweighted B.C.' (235.7, 6, 11)
## 50% 'Unweighted C.C.' (213.4, 5, 12)
## 80% 'Weights'         (2.4, 30, 13)
## 80% 'Weighted B.C.'   (85.0, 12, 9)
## 80% 'Weighted C.C.'   (140.3, 8, 9)
## 80% 'Unweighted B.C.' (120.4, 10, 11)
## 80% 'Unweighted C.C.' (123.2, 9, 9)
## 90% 'Weights'         (1.0, 0, 3)
## 90% 'Weighted B.C.'   (35.9, 20, 8)
## 90% 'Weighted C.C.'   (45.6, 11, 9)
## 90% 'Unweighted B.C.' (48.6, 15, 11)
## 90% 'Unweighted C.C.' (50.6, 12, 9)

#-# parameters:
#-#  Model                   Erdos
#-#  Number_of_nodes         300
#-#  Parameter_of_network    0.125
#-#  Distribution            exponential 0.25
#-#  Immunity_update         1.5 0.8
#-#  N_graph                 5
#-#  N_initial               1
#-#  Number_of_simulations   5
## Averages of all simulations (peak, t_peak, t_half)
## 0% 'Initial'          (248.4, 4, 13)
## 20% 'Weights'         (248.2, 5, 11)
## 20% 'Weighted B.C.'   (250.1, 5, 12)
## 20% 'Weighted C.C.'   (247.2, 5, 12)
## 20% 'Unweighted B.C.' (254.3, 4, 13)
## 20% 'Unweighted C.C.' (253.6, 4, 13)
## 30% 'Weights'         (240.4, 6, 10)
## 30% 'Weighted B.C.'   (239.2, 6, 10)
## 30% 'Weighted C.C.'   (236.6, 4, 13)
## 30% 'Unweighted B.C.' (253.2, 4, 13)
## 30% 'Unweighted C.C.' (252.5, 4, 13)
## 40% 'Weights'         (209.0, 8, 9)
## 40% 'Weighted B.C.'   (202.1, 6, 10)
## 40% 'Weighted C.C.'   (217.1, 5, 12)
## 40% 'Unweighted B.C.' (242.6, 5, 12)
## 40% 'Unweighted C.C.' (231.9, 5, 12)
## 50% 'Weights'         (182.8, 11, 8)
## 50% 'Weighted B.C.'   (179.3, 7, 9)
## 50% 'Weighted C.C.'   (202.2, 6, 11)
## 50% 'Unweighted B.C.' (234.4, 5, 11)
## 50% 'Unweighted C.C.' (195.4, 5, 12)
## 80% 'Weights'         (1.1, 16, 13)
## 80% 'Weighted B.C.'   (40.1, 9, 9)
## 80% 'Weighted C.C.'   (92.7, 8, 10)
## 80% 'Unweighted B.C.' (143.1, 10, 9)
## 80% 'Unweighted C.C.' (121.1, 7, 11)
```

```
## 90% 'Weights'          (1.0, 0, 6)
## 90% 'Weighted B.C.'    (17.8, 10, 10)
## 90% 'Weighted C.C.'    (32.8, 8, 11)
## 90% 'Unweighted B.C.' (67.9, 16, 12)
## 90% 'Unweighted C.C.' (72.8, 10, 9)

#-# parameters:
#-#  Model                    Barabasi
#-#  Number_of_nodes          300
#-#  Parameter_of_network     20
#-#  Distribution             exponential 0.3
#-#  Immunity_update          1.5 0.8
#-#  N_graph                  5
#-#  N_initial                1
#-#  Number_of_simulations    5
## Averages of all simulations (peak, t_peak, t_half)
## 0% 'Initial'           (259.7, 3, 14)
## 20% 'Weights'          (244.6, 4, 12)
## 20% 'Weighted B.C.'    (242.8, 5, 12)
## 20% 'Weighted C.C.'    (248.5, 4, 13)
## 20% 'Unweighted B.C.' (252.6, 4, 13)
## 20% 'Unweighted C.C.' (250.8, 4, 13)
## 30% 'Weights'          (234.8, 6, 10)
## 30% 'Weighted B.C.'    (234.5, 5, 11)
## 30% 'Weighted C.C.'    (230.4, 4, 13)
## 30% 'Unweighted B.C.' (248.7, 4, 13)
## 30% 'Unweighted C.C.' (228.0, 4, 13)
## 40% 'Weights'          (196.6, 8, 9)
## 40% 'Weighted B.C.'    (188.2, 5, 11)
## 40% 'Weighted C.C.'    (242.1, 5, 12)
## 40% 'Unweighted B.C.' (245.3, 5, 12)
## 40% 'Unweighted C.C.' (228.7, 4, 13)
## 50% 'Weights'          (196.7, 9, 9)
## 50% 'Weighted B.C.'    (192.4, 6, 10)
## 50% 'Weighted C.C.'    (223.8, 5, 11)
## 50% 'Unweighted B.C.' (230.9, 6, 11)
## 50% 'Unweighted C.C.' (223.7, 5, 11)
## 80% 'Weights'          (3.6, 32, 14)
## 80% 'Weighted B.C.'    (112.5, 9, 9)
## 80% 'Weighted C.C.'    (121.3, 7, 11)
## 80% 'Unweighted B.C.' (136.1, 11, 9)
## 80% 'Unweighted C.C.' (133.4, 8, 9)
## 90% 'Weights'          (1.0, 0, 7)
## 90% 'Weighted B.C.'    (40.5, 13, 10)
## 90% 'Weighted C.C.'    (65.5, 10, 9)
## 90% 'Unweighted B.C.' (63.9, 15, 11)
## 90% 'Unweighted C.C.' (52.1, 11, 8)

#-# parameters:
#-#  Model                    Erdos
#-#  Number_of_nodes          300
#-#  Parameter_of_network     0.125
#-#  Distribution             exponential 0.3
#-#  Immunity_update          1.5 0.8
#-#  N_graph                  5
#-#  N_initial                1
#-#  Number_of_simulations    5
## Averages of all simulations (peak, t_peak, t_half)
## 0% 'Initial'           (267.4, 3, 14)
## 20% 'Weights'          (248.5, 5, 12)
## 20% 'Weighted B.C.'    (252.1, 4, 12)
## 20% 'Weighted C.C.'    (249.0, 4, 13)
## 20% 'Unweighted B.C.' (250.2, 4, 13)
## 20% 'Unweighted C.C.' (249.2, 4, 13)
## 30% 'Weights'          (241.3, 6, 10)
## 30% 'Weighted B.C.'    (240.1, 5, 11)
## 30% 'Weighted C.C.'    (233.4, 4, 13)
## 30% 'Unweighted B.C.' (255.0, 4, 13)
## 30% 'Unweighted C.C.' (249.8, 4, 13)
## 40% 'Weights'          (221.2, 7, 9)
## 40% 'Weighted B.C.'    (201.6, 5, 12)
## 40% 'Weighted C.C.'    (212.7, 5, 12)
## 40% 'Unweighted B.C.' (245.7, 5, 12)
```

```
## 40% 'Unweighted C.C.' (247.9, 4, 12)
## 50% 'Weights'         (189.6, 10, 8)
## 50% 'Weighted B.C.'   (184.2, 6, 10)
## 50% 'Weighted C.C.'   (176.4, 5, 12)
## 50% 'Unweighted B.C.' (243.1, 5, 11)
## 50% 'Unweighted C.C.' (239.1, 4, 12)
## 80% 'Weights'         (1.0, 0, 6)
## 80% 'Weighted B.C.'   (64.4, 7, 10)
## 80% 'Weighted C.C.'   (128.6, 7, 10)
## 80% 'Unweighted B.C.' (177.0, 9, 9)
## 80% 'Unweighted C.C.' (121.1, 7, 11)
## 90% 'Weights'         (1.0, 0, 3)
## 90% 'Weighted B.C.'   (8.9, 10, 9)
## 90% 'Weighted C.C.'   (59.4, 9, 8)
## 90% 'Unweighted B.C.' (87.0, 14, 11)
## 90% 'Unweighted C.C.' (69.3, 9, 9)

#-# parameters:
#-#  Model                    Barabasi
#-#  Number_of_nodes          300
#-#  Parameter_of_network     20
#-#  Distribution             exponential 0.35
#-#  Immunity_update          1.5 0.8
#-#  N_graph                  5
#-#  N_initial                1
#-#  Number_of_simulations    5
## Averages of all simulations (peak, t_peak, t_half)
## 0% 'Initial'          (265.9, 3, 14)
## 20% 'Weights'         (246.9, 4, 12)
## 20% 'Weighted B.C.'   (247.2, 4, 12)
## 20% 'Weighted C.C.'   (251.6, 3, 14)
## 20% 'Unweighted B.C.' (249.6, 4, 13)
## 20% 'Unweighted C.C.' (239.9, 3, 15)
## 30% 'Weights'         (238.5, 5, 11)
## 30% 'Weighted B.C.'   (217.1, 5, 11)
## 30% 'Weighted C.C.'   (239.6, 4, 13)
## 30% 'Unweighted B.C.' (251.5, 4, 13)
## 30% 'Unweighted C.C.' (236.0, 4, 13)
## 40% 'Weights'         (234.6, 6, 10)
## 40% 'Weighted B.C.'   (190.4, 5, 11)
## 40% 'Weighted C.C.'   (233.3, 5, 12)
## 40% 'Unweighted B.C.' (243.8, 5, 12)
## 40% 'Unweighted C.C.' (224.9, 4, 13)
## 50% 'Weights'         (187.4, 8, 9)
## 50% 'Weighted B.C.'   (173.6, 5, 11)
## 50% 'Weighted C.C.'   (234.6, 5, 12)
## 50% 'Unweighted B.C.' (237.9, 5, 12)
## 50% 'Unweighted C.C.' (218.4, 4, 13)
## 80% 'Weights'         (7.4, 27, 26)
## 80% 'Weighted B.C.'   (77.8, 8, 9)
## 80% 'Weighted C.C.'   (138.6, 7, 10)
## 80% 'Unweighted B.C.' (165.9, 9, 9)
## 80% 'Unweighted C.C.' (133.8, 8, 9)
## 90% 'Weights'         (1.0, 0, 2)
## 90% 'Weighted B.C.'   (38.6, 11, 9)
## 90% 'Weighted C.C.'   (65.2, 8, 9)
## 90% 'Unweighted B.C.' (63.8, 13, 10)
## 90% 'Unweighted C.C.' (73.0, 10, 8)

#-# parameters:
#-#  Model                    Erdos
#-#  Number_of_nodes          300
#-#  Parameter_of_network     0.125
#-#  Distribution             exponential 0.35
#-#  Immunity_update          1.5 0.8
#-#  N_graph                  5
#-#  N_initial                1
#-#  Number_of_simulations    5
## Averages of all simulations (peak, t_peak, t_half)
## 0% 'Initial'          (271.2, 3, 14)
## 20% 'Weights'         (256.4, 4, 12)
## 20% 'Weighted B.C.'   (254.5, 4, 13)
## 20% 'Weighted C.C.'   (247.1, 4, 13)
```

```
## 20% 'Unweighted B.C.' (249.7, 4, 13)
## 20% 'Unweighted C.C.' (249.7, 4, 14)
## 30% 'Weights'         (244.5, 5, 11)
## 30% 'Weighted B.C.'   (217.1, 4, 12)
## 30% 'Weighted C.C.'   (243.4, 4, 13)
## 30% 'Unweighted B.C.' (251.9, 4, 13)
## 30% 'Unweighted C.C.' (246.0, 5, 13)
## 40% 'Weights'         (226.6, 6, 10)
## 40% 'Weighted B.C.'   (199.3, 5, 11)
## 40% 'Weighted C.C.'   (223.8, 5, 12)
## 40% 'Unweighted B.C.' (250.3, 4, 13)
## 40% 'Unweighted C.C.' (246.4, 4, 13)
## 50% 'Weights'         (215.0, 9, 8)
## 50% 'Weighted B.C.'   (176.2, 5, 11)
## 50% 'Weighted C.C.'   (219.1, 5, 12)
## 50% 'Unweighted B.C.' (244.7, 5, 12)
## 50% 'Unweighted C.C.' (223.2, 4, 12)
## 80% 'Weights'         (1.6, 18, 72)
## 80% 'Weighted B.C.'   (61.0, 6, 10)
## 80% 'Weighted C.C.'   (108.0, 6, 10)
## 80% 'Unweighted B.C.' (179.8, 9, 9)
## 80% 'Unweighted C.C.' (159.1, 7, 10)
## 90% 'Weights'         (1.0, 0, 2)
## 90% 'Weighted B.C.'   (32.4, 9, 8)
## 90% 'Weighted C.C.'   (44.8, 8, 10)
## 90% 'Unweighted B.C.' (116.4, 13, 10)
## 90% 'Unweighted C.C.' (55.4, 8, 12)

#-# parameters:
#-#  Model                   Barabasi
#-#  Number_of_nodes         300
#-#  Parameter_of_network    20
#-#  Distribution            exponential 0.4
#-#  Immunity_update         1.5 0.8
#-#  N_graph                 5
#-#  N_initial               1
#-#  Number_of_simulations   5
## Averages of all simulations (peak, t_peak, t_half)
## 0% 'Initial'          (263.4, 3, 14)
## 20% 'Weights'         (254.2, 4, 13)
## 20% 'Weighted B.C.'   (251.9, 4, 12)
## 20% 'Weighted C.C.'   (253.2, 3, 14)
## 20% 'Unweighted B.C.' (250.2, 4, 13)
## 20% 'Unweighted C.C.' (256.6, 3, 14)
## 30% 'Weights'         (242.2, 5, 11)
## 30% 'Weighted B.C.'   (219.2, 4, 12)
## 30% 'Weighted C.C.'   (248.4, 4, 13)
## 30% 'Unweighted B.C.' (252.2, 4, 13)
## 30% 'Unweighted C.C.' (241.2, 4, 14)
## 40% 'Weights'         (226.2, 6, 10)
## 40% 'Weighted B.C.'   (214.2, 5, 12)
## 40% 'Weighted C.C.'   (248.6, 4, 13)
## 40% 'Unweighted B.C.' (243.2, 4, 13)
## 40% 'Unweighted C.C.' (245.0, 4, 13)
## 50% 'Weights'         (204.1, 8, 9)
## 50% 'Weighted B.C.'   (198.8, 5, 12)
## 50% 'Weighted C.C.'   (230.5, 6, 11)
## 50% 'Unweighted B.C.' (239.1, 5, 12)
## 50% 'Unweighted C.C.' (240.9, 4, 12)
## 80% 'Weights'         (7.6, 27, 18)
## 80% 'Weighted B.C.'   (92.6, 8, 9)
## 80% 'Weighted C.C.'   (110.1, 6, 11)
## 80% 'Unweighted B.C.' (141.5, 8, 10)
## 80% 'Unweighted C.C.' (131.8, 7, 10)
## 90% 'Weights'         (1.0, 0, 5)
## 90% 'Weighted B.C.'   (48.4, 10, 9)
## 90% 'Weighted C.C.'   (54.3, 10, 9)
## 90% 'Unweighted B.C.' (70.8, 12, 9)
## 90% 'Unweighted C.C.' (68.6, 9, 9)

#-# parameters:
#-#  Model                   Erdos
#-#  Number_of_nodes         300
```

```
#-#  Parameter_of_network    0.125
#-#  Distribution            exponential 0.4
#-#  Immunity_update         1.5 0.8
#-#  N_graph                 5
#-#  N_initial               1
#-#  Number_of_simulations   5
## Averages of all simulations (peak, t_peak, t_half)
## 0% 'Initial'          (273.1, 3, 14)
## 20% 'Weights'         (251.2, 4, 13)
## 20% 'Weighted B.C.'   (258.6, 4, 12)
## 20% 'Weighted C.C.'   (246.9, 4, 14)
## 20% 'Unweighted B.C.' (258.5, 3, 14)
## 20% 'Unweighted C.C.' (261.4, 3, 14)
## 30% 'Weights'         (241.7, 5, 12)
## 30% 'Weighted B.C.'   (217.7, 4, 13)
## 30% 'Weighted C.C.'   (234.0, 4, 13)
## 30% 'Unweighted B.C.' (254.3, 4, 13)
## 30% 'Unweighted C.C.' (249.4, 4, 13)
## 40% 'Weights'         (231.3, 6, 10)
## 40% 'Weighted B.C.'   (183.7, 4, 12)
## 40% 'Weighted C.C.'   (232.1, 4, 13)
## 40% 'Unweighted B.C.' (252.4, 4, 13)
## 40% 'Unweighted C.C.' (243.8, 5, 12)
## 50% 'Weights'         (217.1, 8, 9)
## 50% 'Weighted B.C.'   (143.1, 4, 12)
## 50% 'Weighted C.C.'   (206.8, 5, 12)
## 50% 'Unweighted B.C.' (243.4, 4, 13)
## 50% 'Unweighted C.C.' (227.3, 4, 14)
## 80% 'Weights'         (4.5, 44, 13)
## 80% 'Weighted B.C.'   (70.4, 6, 10)
## 80% 'Weighted C.C.'   (108.1, 7, 10)
## 80% 'Unweighted B.C.' (188.8, 8, 9)
## 80% 'Unweighted C.C.' (128.2, 7, 10)
## 90% 'Weights'         (1.0, 0, 7)
## 90% 'Weighted B.C.'   (31.2, 8, 8)
## 90% 'Weighted C.C.'   (60.4, 7, 10)
## 90% 'Unweighted B.C.' (74.6, 12, 10)
## 90% 'Unweighted C.C.' (77.7, 8, 10)

#-# parameters:
#-#  Model                   Barabasi
#-#  Number_of_nodes         300
#-#  Parameter_of_network    20
#-#  Distribution            exponential 0.45
#-#  Immunity_update         1.5 0.8
#-#  N_graph                 5
#-#  N_initial               1
#-#  Number_of_simulations   5
## Averages of all simulations (peak, t_peak, t_half)
## 0% 'Initial'          (267.5, 3, 14)
## 20% 'Weights'         (253.7, 4, 12)
## 20% 'Weighted B.C.'   (252.1, 4, 13)
## 20% 'Weighted C.C.'   (257.1, 3, 14)
## 20% 'Unweighted B.C.' (248.9, 4, 14)
## 20% 'Unweighted C.C.' (259.6, 3, 14)
## 30% 'Weights'         (245.6, 5, 11)
## 30% 'Weighted B.C.'   (238.1, 4, 12)
## 30% 'Weighted C.C.'   (249.6, 4, 13)
## 30% 'Unweighted B.C.' (253.0, 4, 13)
## 30% 'Unweighted C.C.' (238.4, 3, 14)
## 40% 'Weights'         (229.3, 6, 11)
## 40% 'Weighted B.C.'   (185.7, 5, 12)
## 40% 'Weighted C.C.'   (247.6, 4, 13)
## 40% 'Unweighted B.C.' (248.5, 4, 13)
## 40% 'Unweighted C.C.' (223.7, 4, 13)
## 50% 'Weights'         (209.8, 7, 10)
## 50% 'Weighted B.C.'   (198.4, 5, 11)
## 50% 'Weighted C.C.'   (239.2, 5, 12)
## 50% 'Unweighted B.C.' (234.4, 5, 12)
## 50% 'Unweighted C.C.' (214.8, 4, 12)
## 80% 'Weights'         (10.6, 34, 23)
## 80% 'Weighted B.C.'   (108.6, 7, 10)
## 80% 'Weighted C.C.'   (125.2, 7, 10)
```

```
## 80% 'Unweighted B.C.' (152.7, 9, 10)
## 80% 'Unweighted C.C.' (146.8, 6, 11)
## 90% 'Weights'         (1.0, 0, 8)
## 90% 'Weighted B.C.'   (36.3, 11, 8)
## 90% 'Weighted C.C.'   (51.2, 7, 12)
## 90% 'Unweighted B.C.' (82.8, 11, 10)
## 90% 'Unweighted C.C.' (76.7, 10, 8)

#-# parameters:
#-#  Model                   Erdos
#-#  Number_of_nodes         300
#-#  Parameter_of_network    0.125
#-#  Distribution            exponential 0.45
#-#  Immunity_update         1.5 0.8
#-#  N_graph                 5
#-#  N_initial               1
#-#  Number_of_simulations   5
## Averages of all simulations (peak, t_peak, t_half)
## 0% 'Initial'          (271.3, 3, 14)
## 20% 'Weights'         (258.6, 4, 12)
## 20% 'Weighted B.C.'   (256.0, 4, 13)
## 20% 'Weighted C.C.'   (249.7, 3, 14)
## 20% 'Unweighted B.C.' (257.0, 3, 14)
## 20% 'Unweighted C.C.' (261.2, 3, 14)
## 30% 'Weights'         (246.8, 5, 11)
## 30% 'Weighted B.C.'   (226.5, 4, 13)
## 30% 'Weighted C.C.'   (238.0, 3, 14)
## 30% 'Unweighted B.C.' (249.6, 4, 13)
## 30% 'Unweighted C.C.' (249.0, 4, 13)
## 40% 'Weights'         (239.3, 6, 10)
## 40% 'Weighted B.C.'   (199.9, 4, 13)
## 40% 'Weighted C.C.'   (239.9, 5, 12)
## 40% 'Unweighted B.C.' (253.3, 4, 13)
## 40% 'Unweighted C.C.' (245.9, 4, 13)
## 50% 'Weights'         (222.0, 8, 9)
## 50% 'Weighted B.C.'   (137.7, 4, 13)
## 50% 'Weighted C.C.'   (230.9, 4, 13)
## 50% 'Unweighted B.C.' (245.0, 5, 12)
## 50% 'Unweighted C.C.' (228.3, 4, 13)
## 80% 'Weights'         (3.8, 20, 31)
## 80% 'Weighted B.C.'   (52.8, 5, 11)
## 80% 'Weighted C.C.'   (81.6, 7, 11)
## 80% 'Unweighted B.C.' (163.2, 8, 9)
## 80% 'Unweighted C.C.' (151.4, 7, 10)
## 90% 'Weights'         (1.0, 0, 5)
## 90% 'Weighted B.C.'   (29.6, 6, 9)
## 90% 'Weighted C.C.'   (54.2, 9, 8)
## 90% 'Unweighted B.C.' (97.3, 12, 9)
## 90% 'Unweighted C.C.' (78.1, 8, 10)

#-# parameters:
#-#  Model                   Barabasi
#-#  Number_of_nodes         300
#-#  Parameter_of_network    20
#-#  Distribution            exponential 0.5
#-#  Immunity_update         1.5 0.8
#-#  N_graph                 5
#-#  N_initial               1
#-#  Number_of_simulations   5
## Averages of all simulations (peak, t_peak, t_half)
## 0% 'Initial'          (264.1, 3, 14)
## 20% 'Weights'         (250.2, 4, 13)
## 20% 'Weighted B.C.'   (236.6, 4, 13)
## 20% 'Weighted C.C.'   (265.0, 3, 14)
## 20% 'Unweighted B.C.' (248.7, 3, 15)
## 20% 'Unweighted C.C.' (262.1, 3, 14)
## 30% 'Weights'         (246.7, 5, 11)
## 30% 'Weighted B.C.'   (223.2, 4, 13)
## 30% 'Weighted C.C.'   (251.2, 4, 13)
## 30% 'Unweighted B.C.' (251.4, 4, 13)
## 30% 'Unweighted C.C.' (251.4, 3, 14)
## 40% 'Weights'         (206.3, 6, 10)
## 40% 'Weighted B.C.'   (218.5, 4, 12)
```

```
## 40% 'Weighted C.C.'   (249.0, 4, 13)
## 40% 'Unweighted B.C.' (251.8, 4, 13)
## 40% 'Unweighted C.C.' (240.8, 4, 13)
## 50% 'Weights'         (214.2, 7, 10)
## 50% 'Weighted B.C.'   (205.3, 4, 12)
## 50% 'Weighted C.C.'   (237.7, 4, 13)
## 50% 'Unweighted B.C.' (243.9, 5, 12)
## 50% 'Unweighted C.C.' (220.3, 4, 13)
## 80% 'Weights'         (20.5, 25, 17)
## 80% 'Weighted B.C.'   (120.0, 7, 9)
## 80% 'Weighted C.C.'   (147.5, 7, 10)
## 80% 'Unweighted B.C.' (175.1, 9, 10)
## 80% 'Unweighted C.C.' (138.3, 7, 10)
## 90% 'Weights'         (1.0, 0, 11)
## 90% 'Weighted B.C.'   (42.7, 10, 10)
## 90% 'Weighted C.C.'   (80.6, 8, 10)
## 90% 'Unweighted B.C.' (82.0, 11, 10)
## 90% 'Unweighted C.C.' (106.9, 8, 10)

#-# parameters:
#-#  Model                    Erdos
#-#  Number_of_nodes          300
#-#  Parameter_of_network     0.125
#-#  Distribution             exponential 0.5
#-#  Immunity_update          1.5 0.8
#-#  N_graph                  5
#-#  N_initial                1
#-#  Number_of_simulations    5
## Averages of all simulations (peak, t_peak, t_half)
## 0% 'Initial'          (272.1, 3, 15)
## 20% 'Weights'         (255.7, 4, 13)
## 20% 'Weighted B.C.'   (252.2, 4, 13)
## 20% 'Weighted C.C.'   (256.8, 3, 15)
## 20% 'Unweighted B.C.' (263.8, 3, 14)
## 20% 'Unweighted C.C.' (268.1, 3, 14)
## 30% 'Weights'         (249.5, 5, 12)
## 30% 'Weighted B.C.'   (227.6, 4, 13)
## 30% 'Weighted C.C.'   (245.5, 4, 14)
## 30% 'Unweighted B.C.' (248.7, 4, 13)
## 30% 'Unweighted C.C.' (249.3, 3, 15)
## 40% 'Weights'         (242.5, 5, 11)
## 40% 'Weighted B.C.'   (174.7, 4, 13)
## 40% 'Weighted C.C.'   (233.1, 4, 13)
## 40% 'Unweighted B.C.' (245.7, 4, 13)
## 40% 'Unweighted C.C.' (245.9, 4, 13)
## 50% 'Weights'         (214.4, 7, 10)
## 50% 'Weighted B.C.'   (145.0, 4, 12)
## 50% 'Weighted C.C.'   (227.7, 5, 12)
## 50% 'Unweighted B.C.' (235.8, 4, 13)
## 50% 'Unweighted C.C.' (242.7, 4, 13)
## 80% 'Weights'         (11.2, 40, 21)
## 80% 'Weighted B.C.'   (39.5, 5, 11)
## 80% 'Weighted C.C.'   (111.4, 6, 10)
## 80% 'Unweighted B.C.' (183.5, 8, 9)
## 80% 'Unweighted C.C.' (143.6, 6, 10)
## 90% 'Weights'         (1.0, 0, 4)
## 90% 'Weighted B.C.'   (16.8, 6, 11)
## 90% 'Weighted C.C.'   (50.8, 6, 10)
## 90% 'Unweighted B.C.' (109.7, 13, 10)
## 90% 'Unweighted C.C.' (67.0, 8, 9)

#-# parameters:
#-#  Model                    Barabasi
#-#  Number_of_nodes          300
#-#  Parameter_of_network     5
#-#  Distribution             exponential 0.1
#-#  Immunity_update          1.5 0.8
#-#  N_graph                  5
#-#  N_initial                1
#-#  Number_of_simulations    5
## Averages of all simulations (peak, t_peak, t_half)
## 0% 'Initial'          (112.5, 12, 10)
## 20% 'Weights'         (45.2, 18, 12)
```

```
## 20% 'Weighted B.C.'   (42.6, 22, 9)
## 20% 'Weighted C.C.'   (71.6, 16, 12)
## 20% 'Unweighted B.C.' (88.6, 17, 9)
## 20% 'Unweighted C.C.' (93.6, 16, 10)
## 30% 'Weights'         (15.8, 23, 13)
## 30% 'Weighted B.C.'   (12.0, 34, 9)
## 30% 'Weighted C.C.'   (66.0, 19, 10)
## 30% 'Unweighted B.C.' (75.4, 19, 10)
## 30% 'Unweighted C.C.' (65.3, 18, 16)
## 40% 'Weights'         (2.9, 20, 19)
## 40% 'Weighted B.C.'   (3.5, 20, 18)
## 40% 'Weighted C.C.'   (35.4, 26, 13)
## 40% 'Unweighted B.C.' (49.6, 24, 14)
## 40% 'Unweighted C.C.' (44.1, 24, 12)
## 50% 'Weights'         (2.0, 12, 19)
## 50% 'Weighted B.C.'   (1.4, 20, 11)
## 50% 'Weighted C.C.'   (22.8, 32, 10)
## 50% 'Unweighted B.C.' (22.3, 33, 12)
## 50% 'Unweighted C.C.' (27.2, 24, 21)
## 80% 'Weights'         (1.0, 0, 2)
## 80% 'Weighted B.C.'   (1.0, 0, 5)
## 80% 'Weighted C.C.'   (1.0, 0, 6)
## 80% 'Unweighted B.C.' (1.0, 0, 12)
## 80% 'Unweighted C.C.' (1.0, 0, 29)
## 90% 'Weights'         (1.0, 0, 3)
## 90% 'Weighted B.C.'   (1.0, 0, 3)
## 90% 'Weighted C.C.'   (1.0, 0, 9)
## 90% 'Unweighted B.C.' (1.0, 0, 5)
## 90% 'Unweighted C.C.' (1.0, 0, 6)

#-# parameters:
#-#  Model                    Barabasi
#-#  Number_of_nodes          600
#-#  Parameter_of_network     5
#-#  Distribution             exponential 0.1
#-#  Immunity_update          1.5 0.8
#-#  N_graph                  5
#-#  N_initial                1
#-#  Number_of_simulations    5
## Averages of all simulations (peak, t_peak, t_half)
## 0% 'Initial'          (211.4, 12, 11)
## 20% 'Weights'         (74.5, 20, 11)
## 20% 'Weighted B.C.'   (116.0, 23, 11)
## 20% 'Weighted C.C.'   (177.1, 17, 11)
## 20% 'Unweighted B.C.' (178.5, 17, 11)
## 20% 'Unweighted C.C.' (188.4, 19, 9)
## 30% 'Weights'         (44.4, 21, 20)
## 30% 'Weighted B.C.'   (58.1, 27, 14)
## 30% 'Weighted C.C.'   (116.3, 23, 10)
## 30% 'Unweighted B.C.' (147.8, 19, 11)
## 30% 'Unweighted C.C.' (127.9, 22, 10)
## 40% 'Weights'         (13.9, 33, 22)
## 40% 'Weighted B.C.'   (5.6, 36, 36)
## 40% 'Weighted C.C.'   (78.5, 24, 15)
## 40% 'Unweighted B.C.' (105.2, 25, 13)
## 40% 'Unweighted C.C.' (105.4, 26, 11)
## 50% 'Weights'         (2.4, 26, 13)
## 50% 'Weighted B.C.'   (1.1, 2, 51)
## 50% 'Weighted C.C.'   (52.4, 28, 17)
## 50% 'Unweighted B.C.' (56.9, 34, 17)
## 50% 'Unweighted C.C.' (55.3, 28, 13)
## 80% 'Weights'         (1.0, 0, 2)
## 80% 'Weighted B.C.'   (1.0, 0, 3)
## 80% 'Weighted C.C.'   (1.1, 2, 12)
## 80% 'Unweighted B.C.' (1.1, 1, 17)
## 80% 'Unweighted C.C.' (1.0, 0, 35)
## 90% 'Weights'         (1.0, 0, 4)
## 90% 'Weighted B.C.'   (1.0, 0, 3)
## 90% 'Weighted C.C.'   (1.0, 0, 14)
## 90% 'Unweighted B.C.' (1.0, 0, 6)
## 90% 'Unweighted C.C.' (1.0, 0, 8)

#-# parameters:
```

```
#-#  Model                  Erdos
#-#  Number_of_nodes        300
#-#  Parameter_of_network   0.033
#-#  Distribution           exponential 0.1
#-#  Immunity_update        1.5 0.8
#-#  N_graph                5
#-#  N_initial              1
#-#  Number_of_simulations  5
## Averages of all simulations (peak, t_peak, t_half)
## 0% 'Initial'          (128.5, 14, 9)
## 20% 'Weights'         (37.0, 25, 16)
## 20% 'Weighted B.C.'   (38.3, 26, 20)
## 20% 'Weighted C.C.'   (81.4, 20, 12)
## 20% 'Unweighted B.C.' (85.8, 18, 10)
## 20% 'Unweighted C.C.' (106.6, 18, 10)
## 30% 'Weights'         (5.9, 40, 13)
## 30% 'Weighted B.C.'   (12.2, 35, 19)
## 30% 'Weighted C.C.'   (44.0, 25, 10)
## 30% 'Unweighted B.C.' (74.3, 20, 10)
## 30% 'Unweighted C.C.' (78.6, 21, 11)
## 40% 'Weights'         (2.8, 20, 17)
## 40% 'Weighted B.C.'   (1.6, 22, 33)
## 40% 'Weighted C.C.'   (29.4, 27, 14)
## 40% 'Unweighted B.C.' (43.2, 22, 11)
## 40% 'Unweighted C.C.' (27.9, 29, 9)
## 50% 'Weights'         (1.0, 0, 6)
## 50% 'Weighted B.C.'   (1.2, 7, 9)
## 50% 'Weighted C.C.'   (15.6, 35, 19)
## 50% 'Unweighted B.C.' (20.3, 29, 12)
## 50% 'Unweighted C.C.' (31.7, 26, 17)
## 80% 'Weights'         (1.0, 0, 3)
## 80% 'Weighted B.C.'   (1.0, 0, 5)
## 80% 'Weighted C.C.'   (1.0, 0, 4)
## 80% 'Unweighted B.C.' (1.0, 0, 16)
## 80% 'Unweighted C.C.' (1.3, 26, 11)
## 90% 'Weights'         (1.0, 0, 4)
## 90% 'Weighted B.C.'   (1.0, 0, 3)
## 90% 'Weighted C.C.'   (1.0, 0, 2)
## 90% 'Unweighted B.C.' (1.0, 0, 4)
## 90% 'Unweighted C.C.' (1.0, 0, 9)

#-# parameters:
#-#  Model                  Erdos
#-#  Number_of_nodes        600
#-#  Parameter_of_network   0.033
#-#  Distribution           exponential 0.1
#-#  Immunity_update        1.5 0.8
#-#  N_graph                5
#-#  N_initial              1
#-#  Number_of_simulations  5
## Averages of all simulations (peak, t_peak, t_half)
## 0% 'Initial'          (433.2, 9, 9)
## 20% 'Weights'         (301.2, 16, 9)
## 20% 'Weighted B.C.'   (247.6, 16, 8)
## 20% 'Weighted C.C.'   (346.0, 12, 8)
## 20% 'Unweighted B.C.' (363.6, 11, 9)
## 20% 'Unweighted C.C.' (372.8, 11, 9)
## 30% 'Weights'         (167.9, 20, 10)
## 30% 'Weighted B.C.'   (168.0, 18, 12)
## 30% 'Weighted C.C.'   (262.9, 13, 9)
## 30% 'Unweighted B.C.' (345.0, 12, 8)
## 30% 'Unweighted C.C.' (321.4, 12, 10)
## 40% 'Weights'         (67.2, 27, 16)
## 40% 'Weighted B.C.'   (70.8, 32, 13)
## 40% 'Weighted C.C.'   (247.4, 13, 9)
## 40% 'Unweighted B.C.' (306.0, 14, 8)
## 40% 'Unweighted C.C.' (305.0, 13, 9)
## 50% 'Weights'         (13.0, 52, 26)
## 50% 'Weighted B.C.'   (11.6, 43, 28)
## 50% 'Weighted C.C.'   (205.2, 16, 9)
## 50% 'Unweighted B.C.' (275.9, 17, 8)
## 50% 'Unweighted C.C.' (245.9, 14, 9)
## 80% 'Weights'         (1.0, 0, 4)
```

```
## 80% 'Weighted B.C.'   (1.0, 0, 4)
## 80% 'Weighted C.C.'   (22.1, 27, 12)
## 80% 'Unweighted B.C.' (19.0, 31, 33)
## 80% 'Unweighted C.C.' (64.1, 25, 13)
## 90% 'Weights'         (1.0, 0, 2)
## 90% 'Weighted B.C.'   (1.0, 0, 3)
## 90% 'Weighted C.C.'   (2.4, 22, 20)
## 90% 'Unweighted B.C.' (1.1, 1, 21)
## 90% 'Unweighted C.C.' (2.6, 34, 25)

#-# parameters:
#-#  Model                  Barabasi
#-#  Number_of_nodes        300
#-#  Parameter_of_network   10
#-#  Distribution           exponential 0.1
#-#  Immunity_update        1.5 0.8
#-#  N_graph                5
#-#  N_initial              1
#-#  Number_of_simulations  5
## Averages of all simulations (peak, t_peak, t_half)
## 0% 'Initial'          (196.0, 8, 9)
## 20% 'Weights'         (115.3, 11, 10)
## 20% 'Weighted B.C.'   (119.0, 12, 9)
## 20% 'Weighted C.C.'   (164.7, 11, 8)
## 20% 'Unweighted B.C.' (173.1, 10, 9)
## 20% 'Unweighted C.C.' (168.8, 10, 9)
## 30% 'Weights'         (65.2, 16, 9)
## 30% 'Weighted B.C.'   (84.4, 15, 10)
## 30% 'Weighted C.C.'   (150.6, 11, 9)
## 30% 'Unweighted B.C.' (154.4, 11, 9)
## 30% 'Unweighted C.C.' (165.5, 12, 8)
## 40% 'Weights'         (40.9, 19, 12)
## 40% 'Weighted B.C.'   (26.4, 23, 11)
## 40% 'Weighted C.C.'   (137.5, 13, 9)
## 40% 'Unweighted B.C.' (143.2, 13, 8)
## 40% 'Unweighted C.C.' (127.6, 13, 9)
## 50% 'Weights'         (13.4, 27, 13)
## 50% 'Weighted B.C.'   (21.5, 24, 17)
## 50% 'Weighted C.C.'   (106.2, 14, 9)
## 50% 'Unweighted B.C.' (114.0, 13, 11)
## 50% 'Unweighted C.C.' (98.9, 17, 8)
## 80% 'Weights'         (1.0, 0, 6)
## 80% 'Weighted B.C.'   (1.0, 0, 9)
## 80% 'Weighted C.C.'   (9.5, 25, 14)
## 80% 'Unweighted B.C.' (11.7, 37, 12)
## 80% 'Unweighted C.C.' (16.4, 27, 16)
## 90% 'Weights'         (1.0, 0, 3)
## 90% 'Weighted B.C.'   (1.1, 1, 5)
## 90% 'Weighted C.C.'   (1.0, 0, 12)
## 90% 'Unweighted B.C.' (1.0, 0, 20)
## 90% 'Unweighted C.C.' (1.2, 10, 6)

#-# parameters:
#-#  Model                  Barabasi
#-#  Number_of_nodes        600
#-#  Parameter_of_network   10
#-#  Distribution           exponential 0.1
#-#  Immunity_update        1.5 0.8
#-#  N_graph                5
#-#  N_initial              1
#-#  Number_of_simulations  5
## Averages of all simulations (peak, t_peak, t_half)
## 0% 'Initial'          (355.1, 9, 9)
## 20% 'Weights'         (234.1, 13, 9)
## 20% 'Weighted B.C.'   (277.4, 14, 8)
## 20% 'Weighted C.C.'   (363.7, 11, 9)
## 20% 'Unweighted B.C.' (327.0, 10, 10)
## 20% 'Unweighted C.C.' (334.8, 11, 9)
## 30% 'Weights'         (141.0, 18, 10)
## 30% 'Weighted B.C.'   (175.2, 17, 10)
## 30% 'Weighted C.C.'   (331.8, 12, 9)
## 30% 'Unweighted B.C.' (331.9, 11, 9)
## 30% 'Unweighted C.C.' (311.6, 11, 10)
```

```
## 40% 'Weights'          (62.3, 21, 13)
## 40% 'Weighted B.C.'    (82.8, 20, 12)
## 40% 'Weighted C.C.'    (249.1, 14, 9)
## 40% 'Unweighted B.C.' (295.9, 13, 9)
## 40% 'Unweighted C.C.' (276.3, 13, 9)
## 50% 'Weights'          (22.4, 35, 13)
## 50% 'Weighted B.C.'    (25.4, 27, 16)
## 50% 'Weighted C.C.'    (177.2, 17, 10)
## 50% 'Unweighted B.C.' (237.3, 15, 9)
## 50% 'Unweighted C.C.' (234.8, 15, 9)
## 80% 'Weights'          (1.0, 0, 5)
## 80% 'Weighted B.C.'    (1.4, 8, 19)
## 80% 'Weighted C.C.'    (36.2, 33, 17)
## 80% 'Unweighted B.C.' (18.1, 35, 25)
## 80% 'Unweighted C.C.' (32.6, 32, 19)
## 90% 'Weights'          (1.0, 0, 3)
## 90% 'Weighted B.C.'    (1.0, 0, 3)
## 90% 'Weighted C.C.'    (1.5, 6, 35)
## 90% 'Unweighted B.C.' (1.6, 9, 9)
## 90% 'Unweighted C.C.' (1.0, 0, 14)

#-# parameters:
#-#  Model                    Erdos
#-#  Number_of_nodes          300
#-#  Parameter_of_network     0.065
#-#  Distribution             exponential 0.1
#-#  Immunity_update          1.5 0.8
#-#  N_graph                  5
#-#  N_initial                1
#-#  Number_of_simulations    5
## Averages of all simulations (peak, t_peak, t_half)
## 0% 'Initial'           (208.4, 8, 9)
## 20% 'Weights'          (123.2, 14, 10)
## 20% 'Weighted B.C.'    (115.2, 13, 9)
## 20% 'Weighted C.C.'    (184.5, 10, 9)
## 20% 'Unweighted B.C.' (186.3, 11, 8)
## 20% 'Unweighted C.C.' (187.0, 11, 8)
## 30% 'Weights'          (81.6, 19, 10)
## 30% 'Weighted B.C.'    (76.2, 19, 9)
## 30% 'Weighted C.C.'    (126.4, 12, 9)
## 30% 'Unweighted B.C.' (177.7, 11, 9)
## 30% 'Unweighted C.C.' (147.1, 11, 9)
## 40% 'Weights'          (27.0, 25, 20)
## 40% 'Weighted B.C.'    (43.0, 22, 18)
## 40% 'Weighted C.C.'    (123.6, 13, 9)
## 40% 'Unweighted B.C.' (140.8, 12, 8)
## 40% 'Unweighted C.C.' (140.3, 12, 9)
## 50% 'Weights'          (3.4, 32, 33)
## 50% 'Weighted B.C.'    (7.8, 26, 38)
## 50% 'Weighted C.C.'    (92.5, 15, 10)
## 50% 'Unweighted B.C.' (107.8, 14, 9)
## 50% 'Unweighted C.C.' (105.3, 14, 9)
## 80% 'Weights'          (1.0, 0, 3)
## 80% 'Weighted B.C.'    (1.0, 0, 4)
## 80% 'Weighted C.C.'    (21.4, 22, 12)
## 80% 'Unweighted B.C.' (10.4, 16, 23)
## 80% 'Unweighted C.C.' (26.5, 25, 12)
## 90% 'Weights'          (1.0, 0, 3)
## 90% 'Weighted B.C.'    (1.0, 0, 3)
## 90% 'Weighted C.C.'    (2.5, 22, 10)
## 90% 'Unweighted B.C.' (1.2, 2, 48)
## 90% 'Unweighted C.C.' (1.0, 0, 22)

#-# parameters:
#-#  Model                    Erdos
#-#  Number_of_nodes          600
#-#  Parameter_of_network     0.065
#-#  Distribution             exponential 0.1
#-#  Immunity_update          1.5 0.8
#-#  N_graph                  5
#-#  N_initial                1
#-#  Number_of_simulations    5
## Averages of all simulations (peak, t_peak, t_half)
```

```
## 0% 'Initial'          (486.7, 6, 11)
## 20% 'Weights'         (417.5, 10, 8)
## 20% 'Weighted B.C.'   (407.6, 9, 9)
## 20% 'Weighted C.C.'   (465.2, 7, 10)
## 20% 'Unweighted B.C.' (472.9, 7, 10)
## 20% 'Unweighted C.C.' (469.1, 7, 10)
## 30% 'Weights'         (349.2, 12, 9)
## 30% 'Weighted B.C.'   (365.5, 11, 9)
## 30% 'Weighted C.C.'   (436.4, 8, 10)
## 30% 'Unweighted B.C.' (458.8, 7, 10)
## 30% 'Unweighted C.C.' (458.9, 7, 10)
## 40% 'Weights'         (221.3, 16, 9)
## 40% 'Weighted B.C.'   (254.7, 15, 10)
## 40% 'Weighted C.C.'   (368.0, 9, 9)
## 40% 'Unweighted B.C.' (398.4, 8, 10)
## 40% 'Unweighted C.C.' (372.8, 8, 10)
## 50% 'Weights'         (149.2, 24, 11)
## 50% 'Weighted B.C.'   (162.8, 20, 10)
## 50% 'Weighted C.C.'   (325.2, 9, 10)
## 50% 'Unweighted B.C.' (403.6, 9, 9)
## 50% 'Unweighted C.C.' (294.9, 10, 10)
## 80% 'Weights'         (1.0, 0, 7)
## 80% 'Weighted B.C.'   (1.0, 0, 7)
## 80% 'Weighted C.C.'   (153.4, 15, 9)
## 80% 'Unweighted B.C.' (163.7, 19, 10)
## 80% 'Unweighted C.C.' (93.6, 15, 10)
## 90% 'Weights'         (1.0, 0, 5)
## 90% 'Weighted B.C.'   (1.0, 0, 7)
## 90% 'Weighted C.C.'   (36.9, 19, 12)
## 90% 'Unweighted B.C.' (19.4, 35, 19)
## 90% 'Unweighted C.C.' (44.5, 15, 11)

#-# parameters:
#-#  Model                  Barabasi
#-#  Number_of_nodes        300
#-#  Parameter_of_network   20
#-#  Distribution           exponential 0.1
#-#  Immunity_update        1.5 0.8
#-#  N_graph                5
#-#  N_initial              1
#-#  Number_of_simulations  5
## Averages of all simulations (peak, t_peak, t_half)
## 0% 'Initial'          (241.0, 5, 11)
## 20% 'Weights'         (200.8, 9, 9)
## 20% 'Weighted B.C.'   (186.0, 9, 9)
## 20% 'Weighted C.C.'   (228.7, 6, 10)
## 20% 'Unweighted B.C.' (225.7, 7, 10)
## 20% 'Unweighted C.C.' (227.8, 7, 10)
## 30% 'Weights'         (146.9, 10, 9)
## 30% 'Weighted B.C.'   (149.6, 11, 8)
## 30% 'Weighted C.C.'   (214.2, 7, 10)
## 30% 'Unweighted B.C.' (224.6, 7, 9)
## 30% 'Unweighted C.C.' (210.5, 7, 10)
## 40% 'Weights'         (125.1, 13, 10)
## 40% 'Weighted B.C.'   (121.9, 13, 9)
## 40% 'Weighted C.C.'   (213.3, 8, 9)
## 40% 'Unweighted B.C.' (185.2, 8, 9)
## 40% 'Unweighted C.C.' (209.8, 8, 9)
## 50% 'Weights'         (70.1, 18, 10)
## 50% 'Weighted B.C.'   (74.0, 15, 12)
## 50% 'Weighted C.C.'   (190.0, 9, 9)
## 50% 'Unweighted B.C.' (175.2, 9, 9)
## 50% 'Unweighted C.C.' (187.6, 8, 9)
## 80% 'Weights'         (1.0, 0, 3)
## 80% 'Weighted B.C.'   (4.1, 22, 16)
## 80% 'Weighted C.C.'   (66.0, 15, 10)
## 80% 'Unweighted B.C.' (80.8, 16, 11)
## 80% 'Unweighted C.C.' (85.7, 14, 10)
## 90% 'Weights'         (1.0, 0, 3)
## 90% 'Weighted B.C.'   (1.0, 0, 17)
## 90% 'Weighted C.C.'   (15.5, 18, 12)
## 90% 'Unweighted B.C.' (16.4, 15, 14)
## 90% 'Unweighted C.C.' (21.6, 22, 11)
```

```
#-# parameters:
#-#  Model                    Barabasi
#-#  Number_of_nodes          600
#-#  Parameter_of_network     20
#-#  Distribution             exponential 0.1
#-#  Immunity_update          1.5 0.8
#-#  N_graph                  5
#-#  N_initial                1
#-#  Number_of_simulations    5
## Averages of all simulations (peak, t_peak, t_half)
## 0% 'Initial'          (479.1, 6, 11)
## 20% 'Weights'         (415.5, 8, 10)
## 20% 'Weighted B.C.'   (357.1, 9, 10)
## 20% 'Weighted C.C.'   (469.6, 7, 10)
## 20% 'Unweighted B.C.' (455.0, 7, 10)
## 20% 'Unweighted C.C.' (468.8, 7, 10)
## 30% 'Weights'         (313.8, 11, 9)
## 30% 'Weighted B.C.'   (277.4, 11, 9)
## 30% 'Weighted C.C.'   (455.4, 7, 10)
## 30% 'Unweighted B.C.' (415.4, 9, 9)
## 30% 'Unweighted C.C.' (453.7, 8, 10)
## 40% 'Weights'         (283.7, 14, 9)
## 40% 'Weighted B.C.'   (241.3, 13, 9)
## 40% 'Weighted C.C.'   (351.0, 8, 10)
## 40% 'Unweighted B.C.' (424.1, 9, 9)
## 40% 'Unweighted C.C.' (335.5, 9, 9)
## 50% 'Weights'         (183.5, 17, 10)
## 50% 'Weighted B.C.'   (178.9, 16, 11)
## 50% 'Weighted C.C.'   (316.6, 9, 10)
## 50% 'Unweighted B.C.' (401.4, 9, 9)
## 50% 'Unweighted C.C.' (395.5, 9, 9)
## 80% 'Weights'         (1.0, 0, 3)
## 80% 'Weighted B.C.'   (2.5, 41, 26)
## 80% 'Weighted C.C.'   (135.4, 15, 10)
## 80% 'Unweighted B.C.' (172.8, 17, 11)
## 80% 'Unweighted C.C.' (177.0, 16, 9)
## 90% 'Weights'         (1.0, 0, 3)
## 90% 'Weighted B.C.'   (1.0, 0, 6)
## 90% 'Weighted C.C.'   (50.3, 22, 12)
## 90% 'Unweighted B.C.' (42.0, 25, 13)
## 90% 'Unweighted C.C.' (62.4, 22, 14)

#-# parameters:
#-#  Model                    Erdos
#-#  Number_of_nodes          300
#-#  Parameter_of_network     0.125
#-#  Distribution             exponential 0.1
#-#  Immunity_update          1.5 0.8
#-#  N_graph                  5
#-#  N_initial                1
#-#  Number_of_simulations    5
## Averages of all simulations (peak, t_peak, t_half)
## 0% 'Initial'          (242.3, 5, 11)
## 20% 'Weights'         (200.4, 9, 9)
## 20% 'Weighted B.C.'   (210.5, 9, 9)
## 20% 'Weighted C.C.'   (225.4, 7, 10)
## 20% 'Unweighted B.C.' (226.0, 6, 10)
## 20% 'Unweighted C.C.' (220.0, 6, 11)
## 30% 'Weights'         (153.0, 12, 8)
## 30% 'Weighted B.C.'   (143.9, 12, 8)
## 30% 'Weighted C.C.'   (215.0, 7, 10)
## 30% 'Unweighted B.C.' (228.0, 7, 10)
## 30% 'Unweighted C.C.' (210.0, 7, 9)
## 40% 'Weights'         (127.6, 15, 9)
## 40% 'Weighted B.C.'   (136.4, 16, 8)
## 40% 'Weighted C.C.'   (176.1, 8, 9)
## 40% 'Unweighted B.C.' (219.8, 8, 9)
## 40% 'Unweighted C.C.' (206.6, 8, 9)
## 50% 'Weights'         (64.3, 23, 10)
## 50% 'Weighted B.C.'   (75.0, 22, 10)
## 50% 'Weighted C.C.'   (143.2, 10, 8)
## 50% 'Unweighted B.C.' (199.8, 9, 9)
```

```
## 50% 'Unweighted C.C.' (150.0, 9, 10)
## 80% 'Weights'         (1.0, 0, 5)
## 80% 'Weighted B.C.'   (1.0, 0, 10)
## 80% 'Weighted C.C.'   (53.4, 14, 9)
## 80% 'Unweighted B.C.' (65.8, 18, 10)
## 80% 'Unweighted C.C.' (82.6, 11, 9)
## 90% 'Weights'         (1.0, 0, 2)
## 90% 'Weighted B.C.'   (1.0, 0, 4)
## 90% 'Weighted C.C.'   (13.0, 19, 10)
## 90% 'Unweighted B.C.' (9.2, 32, 27)
## 90% 'Unweighted C.C.' (25.2, 19, 8)

#-# parameters:
#-#  Model                   Erdos
#-#  Number_of_nodes         600
#-#  Parameter_of_network    0.125
#-#  Distribution            exponential 0.1
#-#  Immunity_update         1.5 0.8
#-#  N_graph                 5
#-#  N_initial               1
#-#  Number_of_simulations   5
## Averages of all simulations (peak, t_peak, t_half)
## 0% 'Initial'          (525.8, 4, 13)
## 20% 'Weights'         (479.0, 6, 11)
## 20% 'Weighted B.C.'   (467.0, 6, 11)
## 20% 'Weighted C.C.'   (490.8, 5, 12)
## 20% 'Unweighted B.C.' (481.2, 4, 13)
## 20% 'Unweighted C.C.' (493.3, 5, 12)
## 30% 'Weights'         (438.1, 8, 9)
## 30% 'Weighted B.C.'   (446.2, 7, 10)
## 30% 'Weighted C.C.'   (443.4, 5, 12)
## 30% 'Unweighted B.C.' (496.7, 5, 12)
## 30% 'Unweighted C.C.' (474.2, 6, 11)
## 40% 'Weights'         (380.1, 10, 9)
## 40% 'Weighted B.C.'   (367.5, 9, 10)
## 40% 'Weighted C.C.'   (460.8, 6, 11)
## 40% 'Unweighted B.C.' (480.8, 6, 11)
## 40% 'Unweighted C.C.' (458.4, 5, 12)
## 50% 'Weights'         (237.6, 13, 8)
## 50% 'Weighted B.C.'   (366.4, 12, 9)
## 50% 'Weighted C.C.'   (365.7, 7, 10)
## 50% 'Unweighted B.C.' (471.7, 6, 11)
## 50% 'Unweighted C.C.' (450.0, 6, 11)
## 80% 'Weights'         (1.0, 0, 6)
## 80% 'Weighted B.C.'   (50.0, 26, 9)
## 80% 'Weighted C.C.'   (169.1, 10, 9)
## 80% 'Unweighted B.C.' (365.4, 10, 9)
## 80% 'Unweighted C.C.' (176.8, 10, 9)
## 90% 'Weights'         (1.0, 0, 3)
## 90% 'Weighted B.C.'   (18.4, 32, 12)
## 90% 'Weighted C.C.'   (68.0, 11, 11)
## 90% 'Unweighted B.C.' (149.3, 18, 11)
## 90% 'Unweighted C.C.' (96.5, 11, 9)

#-# parameters:
#-#  Model                   Barabasi
#-#  Number_of_nodes         300
#-#  Parameter_of_network    30
#-#  Distribution            exponential 0.1
#-#  Immunity_update         1.5 0.8
#-#  N_graph                 5
#-#  N_initial               1
#-#  Number_of_simulations   5
## Averages of all simulations (peak, t_peak, t_half)
## 0% 'Initial'          (246.8, 4, 12)
## 20% 'Weights'         (218.3, 7, 9)
## 20% 'Weighted B.C.'   (227.6, 7, 10)
## 20% 'Weighted C.C.'   (241.0, 5, 11)
## 20% 'Unweighted B.C.' (239.6, 5, 11)
## 20% 'Unweighted C.C.' (231.0, 5, 11)
## 30% 'Weights'         (187.3, 8, 10)
## 30% 'Weighted B.C.'   (194.1, 8, 9)
## 30% 'Weighted C.C.'   (240.1, 6, 10)
```

```
## 30% 'Unweighted B.C.' (236.0, 6, 11)
## 30% 'Unweighted C.C.' (231.4, 5, 11)
## 40% 'Weights'         (155.4, 11, 9)
## 40% 'Weighted B.C.'   (170.2, 10, 9)
## 40% 'Weighted C.C.'   (220.5, 6, 10)
## 40% 'Unweighted B.C.' (227.5, 6, 10)
## 40% 'Unweighted C.C.' (205.8, 6, 10)
## 50% 'Weights'         (103.8, 14, 9)
## 50% 'Weighted B.C.'   (130.7, 11, 10)
## 50% 'Weighted C.C.'   (211.6, 6, 11)
## 50% 'Unweighted B.C.' (201.4, 7, 11)
## 50% 'Unweighted C.C.' (175.0, 7, 10)
## 80% 'Weights'         (1.0, 0, 5)
## 80% 'Weighted B.C.'   (15.8, 17, 17)
## 80% 'Weighted C.C.'   (86.6, 11, 9)
## 80% 'Unweighted B.C.' (61.4, 14, 9)
## 80% 'Unweighted C.C.' (89.2, 10, 9)
## 90% 'Weights'         (1.0, 0, 5)
## 90% 'Weighted B.C.'   (1.0, 0, 18)
## 90% 'Weighted C.C.'   (45.8, 15, 10)
## 90% 'Unweighted B.C.' (19.7, 13, 12)
## 90% 'Unweighted C.C.' (65.7, 13, 9)

#-# parameters:
#-#  Model                   Barabasi
#-#  Number_of_nodes         600
#-#  Parameter_of_network    30
#-#  Distribution            exponential 0.1
#-#  Immunity_update         1.5 0.8
#-#  N_graph                 5
#-#  N_initial               1
#-#  Number_of_simulations   5
## Averages of all simulations (peak, t_peak, t_half)
## 0% 'Initial'          (473.9, 4, 13)
## 20% 'Weights'         (443.0, 7, 10)
## 20% 'Weighted B.C.'   (440.3, 7, 10)
## 20% 'Weighted C.C.'   (497.3, 5, 12)
## 20% 'Unweighted B.C.' (463.6, 5, 12)
## 20% 'Unweighted C.C.' (487.9, 5, 12)
## 30% 'Weights'         (384.2, 9, 9)
## 30% 'Weighted B.C.'   (384.8, 8, 10)
## 30% 'Weighted C.C.'   (457.2, 6, 11)
## 30% 'Unweighted B.C.' (473.2, 6, 10)
## 30% 'Unweighted C.C.' (435.2, 6, 11)
## 40% 'Weights'         (329.8, 10, 9)
## 40% 'Weighted B.C.'   (324.4, 11, 9)
## 40% 'Weighted C.C.'   (469.3, 6, 11)
## 40% 'Unweighted B.C.' (434.9, 7, 10)
## 40% 'Unweighted C.C.' (444.4, 6, 11)
## 50% 'Weights'         (216.9, 15, 9)
## 50% 'Weighted B.C.'   (241.8, 14, 9)
## 50% 'Weighted C.C.'   (428.6, 7, 10)
## 50% 'Unweighted B.C.' (435.6, 9, 9)
## 50% 'Unweighted C.C.' (440.2, 7, 10)
## 80% 'Weights'         (1.0, 0, 5)
## 80% 'Weighted B.C.'   (28.5, 26, 19)
## 80% 'Weighted C.C.'   (250.0, 11, 9)
## 80% 'Unweighted B.C.' (172.6, 13, 11)
## 80% 'Unweighted C.C.' (205.7, 10, 9)
## 90% 'Weights'         (1.0, 0, 2)
## 90% 'Weighted B.C.'   (1.0, 0, 17)
## 90% 'Weighted C.C.'   (81.9, 15, 11)
## 90% 'Unweighted B.C.' (55.8, 20, 12)
## 90% 'Unweighted C.C.' (113.8, 16, 10)

#-# parameters:
#-#  Model                   Erdos
#-#  Number_of_nodes         300
#-#  Parameter_of_network    0.18
#-#  Distribution            exponential 0.1
#-#  Immunity_update         1.5 0.8
#-#  N_graph                 5
#-#  N_initial               1
```

```
#-#  Number_of_simulations   5
## Averages of all simulations (peak, t_peak, t_half)
## 0% 'Initial'          (238.6, 4, 13)
## 20% 'Weights'         (221.0, 7, 10)
## 20% 'Weighted B.C.'   (226.4, 7, 10)
## 20% 'Weighted C.C.'   (238.7, 6, 11)
## 20% 'Unweighted B.C.' (238.1, 5, 11)
## 20% 'Unweighted C.C.' (239.3, 5, 12)
## 30% 'Weights'         (214.0, 8, 10)
## 30% 'Weighted B.C.'   (214.0, 8, 9)
## 30% 'Weighted C.C.'   (221.4, 6, 10)
## 30% 'Unweighted B.C.' (237.6, 6, 10)
## 30% 'Unweighted C.C.' (218.4, 5, 11)
## 40% 'Weights'         (186.2, 11, 9)
## 40% 'Weighted B.C.'   (179.0, 11, 9)
## 40% 'Weighted C.C.'   (213.4, 6, 11)
## 40% 'Unweighted B.C.' (233.0, 6, 11)
## 40% 'Unweighted C.C.' (217.4, 6, 11)
## 50% 'Weights'         (94.5, 16, 11)
## 50% 'Weighted B.C.'   (121.6, 15, 8)
## 50% 'Weighted C.C.'   (191.6, 7, 10)
## 50% 'Unweighted B.C.' (204.5, 7, 10)
## 50% 'Unweighted C.C.' (171.6, 7, 10)
## 80% 'Weights'         (1.0, 0, 9)
## 80% 'Weighted B.C.'   (13.4, 30, 14)
## 80% 'Weighted C.C.'   (83.2, 11, 8)
## 80% 'Unweighted B.C.' (117.6, 12, 9)
## 80% 'Unweighted C.C.' (83.7, 9, 10)
## 90% 'Weights'         (1.0, 0, 3)
## 90% 'Weighted B.C.'   (2.8, 16, 26)
## 90% 'Weighted C.C.'   (23.2, 13, 12)
## 90% 'Unweighted B.C.' (40.2, 17, 13)
## 90% 'Unweighted C.C.' (31.9, 12, 11)

#-# parameters:
#-#  Model                  Erdos
#-#  Number_of_nodes        600
#-#  Parameter_of_network   0.18
#-#  Distribution           exponential 0.1
#-#  Immunity_update        1.5 0.8
#-#  N_graph                5
#-#  N_initial              1
#-#  Number_of_simulations   5
## Averages of all simulations (peak, t_peak, t_half)
## 0% 'Initial'          (505.6, 3, 15)
## 20% 'Weights'         (500.2, 5, 12)
## 20% 'Weighted B.C.'   (500.2, 5, 12)
## 20% 'Weighted C.C.'   (507.0, 4, 13)
## 20% 'Unweighted B.C.' (514.2, 4, 13)
## 20% 'Unweighted C.C.' (507.8, 4, 13)
## 30% 'Weights'         (470.4, 6, 11)
## 30% 'Weighted B.C.'   (464.3, 6, 11)
## 30% 'Weighted C.C.'   (469.9, 4, 13)
## 30% 'Unweighted B.C.' (513.3, 4, 13)
## 30% 'Unweighted C.C.' (484.6, 5, 13)
## 40% 'Weights'         (437.9, 7, 10)
## 40% 'Weighted B.C.'   (466.0, 7, 10)
## 40% 'Weighted C.C.'   (422.3, 6, 12)
## 40% 'Unweighted B.C.' (493.8, 5, 12)
## 40% 'Unweighted C.C.' (462.2, 5, 12)
## 50% 'Weights'         (413.1, 10, 9)
## 50% 'Weighted B.C.'   (412.2, 9, 9)
## 50% 'Weighted C.C.'   (385.0, 6, 11)
## 50% 'Unweighted B.C.' (494.3, 5, 12)
## 50% 'Unweighted C.C.' (449.4, 5, 12)
## 80% 'Weights'         (3.1, 42, 46)
## 80% 'Weighted B.C.'   (174.6, 13, 9)
## 80% 'Weighted C.C.'   (255.1, 7, 10)
## 80% 'Unweighted B.C.' (427.5, 8, 10)
## 80% 'Unweighted C.C.' (296.8, 7, 11)
## 90% 'Weights'         (1.0, 0, 4)
## 90% 'Weighted B.C.'   (35.3, 17, 10)
## 90% 'Weighted C.C.'   (84.2, 9, 9)
```

```
## 90% 'Unweighted B.C.' (222.0, 14, 8)
## 90% 'Unweighted C.C.' (105.1, 9, 10)

#-# parameters:
#-#  Model                   Barabasi
#-#  Number_of_nodes         300
#-#  Parameter_of_network    50
#-#  Distribution            exponential 0.1
#-#  Immunity_update         1.5 0.8
#-#  N_graph                 5
#-#  N_initial               1
#-#  Number_of_simulations   5
## Averages of all simulations (peak, t_peak, t_half)
## 0% 'Initial'          (249.3, 4, 13)
## 20% 'Weights'         (231.8, 6, 11)
## 20% 'Weighted B.C.'   (240.8, 5, 11)
## 20% 'Weighted C.C.'   (248.3, 4, 13)
## 20% 'Unweighted B.C.' (249.5, 4, 13)
## 20% 'Unweighted C.C.' (240.2, 4, 13)
## 30% 'Weights'         (212.8, 6, 10)
## 30% 'Weighted B.C.'   (230.7, 7, 10)
## 30% 'Weighted C.C.'   (246.4, 4, 13)
## 30% 'Unweighted B.C.' (243.2, 5, 12)
## 30% 'Unweighted C.C.' (230.7, 4, 13)
## 40% 'Weights'         (196.9, 8, 9)
## 40% 'Weighted B.C.'   (210.4, 8, 9)
## 40% 'Weighted C.C.'   (241.0, 5, 11)
## 40% 'Unweighted B.C.' (230.4, 5, 11)
## 40% 'Unweighted C.C.' (201.6, 4, 13)
## 50% 'Weights'         (147.1, 12, 8)
## 50% 'Weighted B.C.'   (170.5, 9, 9)
## 50% 'Weighted C.C.'   (235.5, 5, 11)
## 50% 'Unweighted B.C.' (225.4, 7, 10)
## 50% 'Unweighted C.C.' (190.7, 5, 11)
## 80% 'Weights'         (1.1, 2, 6)
## 80% 'Weighted B.C.'   (48.2, 14, 11)
## 80% 'Weighted C.C.'   (89.7, 8, 9)
## 80% 'Unweighted B.C.' (73.0, 12, 12)
## 80% 'Unweighted C.C.' (102.2, 7, 9)
## 90% 'Weights'         (1.0, 0, 4)
## 90% 'Weighted B.C.'   (12.7, 31, 9)
## 90% 'Weighted C.C.'   (60.0, 10, 9)
## 90% 'Unweighted B.C.' (18.7, 10, 10)
## 90% 'Unweighted C.C.' (43.2, 9, 10)

#-# parameters:
#-#  Model                   Barabasi
#-#  Number_of_nodes         600
#-#  Parameter_of_network    50
#-#  Distribution            exponential 0.1
#-#  Immunity_update         1.5 0.8
#-#  N_graph                 5
#-#  N_initial               1
#-#  Number_of_simulations   5
## Averages of all simulations (peak, t_peak, t_half)
## 0% 'Initial'          (503.2, 4, 13)
## 20% 'Weights'         (480.4, 6, 11)
## 20% 'Weighted B.C.'   (488.4, 5, 11)
## 20% 'Weighted C.C.'   (514.4, 4, 13)
## 20% 'Unweighted B.C.' (489.8, 5, 12)
## 20% 'Unweighted C.C.' (487.0, 4, 13)
## 30% 'Weights'         (459.3, 7, 10)
## 30% 'Weighted B.C.'   (446.6, 7, 10)
## 30% 'Weighted C.C.'   (497.2, 4, 13)
## 30% 'Unweighted B.C.' (493.8, 5, 12)
## 30% 'Unweighted C.C.' (471.2, 4, 13)
## 40% 'Weights'         (442.6, 8, 9)
## 40% 'Weighted B.C.'   (413.8, 8, 9)
## 40% 'Weighted C.C.'   (493.5, 5, 12)
## 40% 'Unweighted B.C.' (484.6, 5, 12)
## 40% 'Unweighted C.C.' (425.2, 5, 12)
## 50% 'Weights'         (369.0, 10, 9)
## 50% 'Weighted B.C.'   (316.4, 9, 10)
```

```
## 50% 'Weighted C.C.'   (435.8, 5, 12)
## 50% 'Unweighted B.C.' (446.2, 6, 11)
## 50% 'Unweighted C.C.' (427.2, 5, 12)
## 80% 'Weights'         (2.2, 53, 19)
## 80% 'Weighted B.C.'   (120.2, 16, 11)
## 80% 'Weighted C.C.'   (289.4, 10, 9)
## 80% 'Unweighted B.C.' (162.3, 12, 11)
## 80% 'Unweighted C.C.' (235.6, 7, 10)
## 90% 'Weights'         (1.0, 0, 3)
## 90% 'Weighted B.C.'   (28.0, 29, 16)
## 90% 'Weighted C.C.'   (174.2, 10, 9)
## 90% 'Unweighted B.C.' (52.9, 9, 16)
## 90% 'Unweighted C.C.' (138.8, 9, 9)

#-# parameters:
#-#  Model                   Erdos
#-#  Number_of_nodes         300
#-#  Parameter_of_network    0.3
#-#  Distribution            exponential 0.1
#-#  Immunity_update         1.5 0.8
#-#  N_graph                 5
#-#  N_initial               1
#-#  Number_of_simulations   5
## Averages of all simulations (peak, t_peak, t_half)
## 0% 'Initial'          (264.0, 3, 14)
## 20% 'Weights'         (243.6, 5, 12)
## 20% 'Weighted B.C.'   (247.2, 5, 11)
## 20% 'Weighted C.C.'   (251.8, 4, 13)
## 20% 'Unweighted B.C.' (255.1, 4, 13)
## 20% 'Unweighted C.C.' (245.4, 4, 13)
## 30% 'Weights'         (232.8, 6, 10)
## 30% 'Weighted B.C.'   (238.1, 6, 10)
## 30% 'Weighted C.C.'   (212.7, 4, 13)
## 30% 'Unweighted B.C.' (252.5, 4, 12)
## 30% 'Unweighted C.C.' (247.7, 4, 13)
## 40% 'Weights'         (200.3, 8, 9)
## 40% 'Weighted B.C.'   (225.9, 8, 9)
## 40% 'Weighted C.C.'   (222.4, 5, 12)
## 40% 'Unweighted B.C.' (244.4, 5, 12)
## 40% 'Unweighted C.C.' (241.3, 4, 13)
## 50% 'Weights'         (180.9, 10, 9)
## 50% 'Weighted B.C.'   (169.3, 9, 9)
## 50% 'Weighted C.C.'   (205.4, 5, 11)
## 50% 'Unweighted B.C.' (236.2, 6, 11)
## 50% 'Unweighted C.C.' (208.4, 6, 11)
## 80% 'Weights'         (1.8, 20, 37)
## 80% 'Weighted B.C.'   (75.2, 12, 10)
## 80% 'Weighted C.C.'   (89.6, 8, 10)
## 80% 'Unweighted B.C.' (152.9, 9, 9)
## 80% 'Unweighted C.C.' (123.5, 7, 11)
## 90% 'Weights'         (1.0, 0, 2)
## 90% 'Weighted B.C.'   (15.4, 18, 7)
## 90% 'Weighted C.C.'   (50.6, 8, 11)
## 90% 'Unweighted B.C.' (93.0, 14, 9)
## 90% 'Unweighted C.C.' (48.2, 9, 9)

#-# parameters:
#-#  Model                   Erdos
#-#  Number_of_nodes         600
#-#  Parameter_of_network    0.3
#-#  Distribution            exponential 0.1
#-#  Immunity_update         1.5 0.8
#-#  N_graph                 5
#-#  N_initial               1
#-#  Number_of_simulations   5
## Averages of all simulations (peak, t_peak, t_half)
## 0% 'Initial'          (546.0, 3, 16)
## 20% 'Weights'         (515.0, 4, 13)
## 20% 'Weighted B.C.'   (521.3, 4, 13)
## 20% 'Weighted C.C.'   (531.9, 3, 15)
## 20% 'Unweighted B.C.' (551.1, 3, 15)
## 20% 'Unweighted C.C.' (539.7, 3, 15)
## 30% 'Weights'         (513.3, 4, 13)
```

```
## 30% 'Weighted B.C.'   (515.0, 4, 13)
## 30% 'Weighted C.C.'   (498.7, 3, 15)
## 30% 'Unweighted B.C.' (512.5, 3, 15)
## 30% 'Unweighted C.C.' (506.4, 3, 15)
## 40% 'Weights'         (481.4, 6, 11)
## 40% 'Weighted B.C.'   (500.1, 5, 12)
## 40% 'Weighted C.C.'   (476.4, 5, 13)
## 40% 'Unweighted B.C.' (499.7, 3, 15)
## 40% 'Unweighted C.C.' (469.8, 3, 15)
## 50% 'Weights'         (473.3, 7, 10)
## 50% 'Weighted B.C.'   (360.3, 5, 12)
## 50% 'Weighted C.C.'   (436.5, 4, 14)
## 50% 'Unweighted B.C.' (512.5, 4, 13)
## 50% 'Unweighted C.C.' (480.8, 5, 13)
## 80% 'Weights'         (66.0, 36, 13)
## 80% 'Weighted B.C.'   (188.8, 8, 9)
## 80% 'Weighted C.C.'   (217.3, 6, 11)
## 80% 'Unweighted B.C.' (445.0, 6, 11)
## 80% 'Unweighted C.C.' (276.1, 6, 11)
## 90% 'Weights'         (1.0, 0, 2)
## 90% 'Weighted B.C.'   (91.7, 8, 9)
## 90% 'Weighted C.C.'   (147.9, 9, 10)
## 90% 'Unweighted B.C.' (308.7, 9, 10)
## 90% 'Unweighted C.C.' (146.3, 8, 9)

#-# parameters:
#-#  Model                   Barabasi
#-#  Number_of_nodes         600
#-#  Parameter_of_network    5
#-#  Distribution            exponential 0.1
#-#  Immunity_update         1.5 0.8
#-#  N_graph                 5
#-#  N_initial               1
#-#  Number_of_simulations   5
## Averages of all simulations (peak, t_peak, t_half)
## 0% 'Initial'          (211.4, 12, 11)
## 20% 'Weights'         (74.5, 20, 11)
## 20% 'Weighted B.C.'   (116.0, 23, 11)
## 20% 'Weighted C.C.'   (177.1, 17, 11)
## 20% 'Unweighted B.C.' (178.5, 17, 11)
## 20% 'Unweighted C.C.' (188.4, 19, 9)
## 30% 'Weights'         (44.4, 21, 20)
## 30% 'Weighted B.C.'   (58.1, 27, 14)
## 30% 'Weighted C.C.'   (116.3, 23, 10)
## 30% 'Unweighted B.C.' (147.8, 19, 11)
## 30% 'Unweighted C.C.' (127.9, 22, 10)
## 40% 'Weights'         (13.9, 33, 22)
## 40% 'Weighted B.C.'   (5.6, 36, 36)
## 40% 'Weighted C.C.'   (78.5, 24, 15)
## 40% 'Unweighted B.C.' (105.2, 25, 13)
## 40% 'Unweighted C.C.' (105.4, 26, 11)
## 50% 'Weights'         (2.4, 26, 13)
## 50% 'Weighted B.C.'   (1.1, 2, 51)
## 50% 'Weighted C.C.'   (52.4, 28, 17)
## 50% 'Unweighted B.C.' (56.9, 34, 17)
## 50% 'Unweighted C.C.' (55.3, 28, 13)
## 80% 'Weights'         (1.0, 0, 2)
## 80% 'Weighted B.C.'   (1.0, 0, 3)
## 80% 'Weighted C.C.'   (1.1, 2, 12)
## 80% 'Unweighted B.C.' (1.1, 1, 17)
## 80% 'Unweighted C.C.' (1.0, 0, 35)
## 90% 'Weights'         (1.0, 0, 4)
## 90% 'Weighted B.C.'   (1.0, 0, 3)
## 90% 'Weighted C.C.'   (1.0, 0, 14)
## 90% 'Unweighted B.C.' (1.0, 0, 6)
## 90% 'Unweighted C.C.' (1.0, 0, 8)

#-# parameters:
#-#  Model                   Erdos
#-#  Number_of_nodes         600
#-#  Parameter_of_network    0.01625
#-#  Distribution            exponential 0.1
#-#  Immunity_update         1.5 0.8
```

```
#-#  N_graph                 5
#-#  N_initial               1
#-#  Number_of_simulations   5
## Averages of all simulations (peak, t_peak, t_half)
## 0% 'Initial'          (274.0, 16, 9)
## 20% 'Weights'         (53.2, 34, 14)
## 20% 'Weighted B.C.'   (80.8, 30, 14)
## 20% 'Weighted C.C.'   (152.2, 23, 10)
## 20% 'Unweighted B.C.' (164.0, 19, 10)
## 20% 'Unweighted C.C.' (198.8, 22, 10)
## 30% 'Weights'         (8.6, 59, 14)
## 30% 'Weighted B.C.'   (14.5, 42, 26)
## 30% 'Weighted C.C.'   (100.9, 28, 13)
## 30% 'Unweighted B.C.' (148.7, 23, 12)
## 30% 'Unweighted C.C.' (149.1, 24, 11)
## 40% 'Weights'         (1.8, 13, 41)
## 40% 'Weighted B.C.'   (1.2, 33, 45)
## 40% 'Weighted C.C.'   (69.4, 31, 14)
## 40% 'Unweighted B.C.' (80.3, 26, 15)
## 40% 'Unweighted C.C.' (98.0, 26, 11)
## 50% 'Weights'         (1.0, 0, 5)
## 50% 'Weighted B.C.'   (1.0, 0, 19)
## 50% 'Weighted C.C.'   (19.2, 44, 13)
## 50% 'Unweighted B.C.' (43.3, 35, 16)
## 50% 'Unweighted C.C.' (42.1, 35, 16)
## 80% 'Weights'         (1.0, 0, 3)
## 80% 'Weighted B.C.'   (1.0, 0, 4)
## 80% 'Weighted C.C.'   (1.0, 0, 11)
## 80% 'Unweighted B.C.' (1.0, 0, 5)
## 80% 'Unweighted C.C.' (1.1, 1, 19)
## 90% 'Weights'         (1.0, 0, 4)
## 90% 'Weighted B.C.'   (1.0, 0, 4)
## 90% 'Weighted C.C.'   (1.0, 0, 5)
## 90% 'Unweighted B.C.' (1.0, 0, 7)
## 90% 'Unweighted C.C.' (1.0, 0, 14)

#-# parameters:
#-#  Model                   Barabasi
#-#  Number_of_nodes         600
#-#  Parameter_of_network    10
#-#  Distribution            exponential 0.1
#-#  Immunity_update         1.5 0.8
#-#  N_graph                 5
#-#  N_initial               1
#-#  Number_of_simulations   5
## Averages of all simulations (peak, t_peak, t_half)
## 0% 'Initial'          (355.1, 9, 9)
## 20% 'Weights'         (234.1, 13, 9)
## 20% 'Weighted B.C.'   (277.4, 14, 8)
## 20% 'Weighted C.C.'   (363.7, 11, 9)
## 20% 'Unweighted B.C.' (327.0, 10, 10)
## 20% 'Unweighted C.C.' (334.8, 11, 9)
## 30% 'Weights'         (141.0, 18, 10)
## 30% 'Weighted B.C.'   (175.2, 17, 10)
## 30% 'Weighted C.C.'   (331.8, 12, 9)
## 30% 'Unweighted B.C.' (331.9, 11, 9)
## 30% 'Unweighted C.C.' (311.6, 11, 10)
## 40% 'Weights'         (62.3, 21, 13)
## 40% 'Weighted B.C.'   (82.8, 20, 12)
## 40% 'Weighted C.C.'   (249.1, 14, 9)
## 40% 'Unweighted B.C.' (295.9, 13, 9)
## 40% 'Unweighted C.C.' (276.3, 13, 9)
## 50% 'Weights'         (22.4, 35, 13)
## 50% 'Weighted B.C.'   (25.4, 27, 16)
## 50% 'Weighted C.C.'   (177.2, 17, 10)
## 50% 'Unweighted B.C.' (237.3, 15, 9)
## 50% 'Unweighted C.C.' (234.8, 15, 9)
## 80% 'Weights'         (1.0, 0, 5)
## 80% 'Weighted B.C.'   (1.4, 8, 19)
## 80% 'Weighted C.C.'   (36.2, 33, 17)
## 80% 'Unweighted B.C.' (18.1, 35, 25)
## 80% 'Unweighted C.C.' (32.6, 32, 19)
## 90% 'Weights'         (1.0, 0, 3)
```

```
## 90% 'Weighted B.C.'   (1.0, 0, 3)
## 90% 'Weighted C.C.'   (1.5, 6, 35)
## 90% 'Unweighted B.C.' (1.6, 9, 9)
## 90% 'Unweighted C.C.' (1.0, 0, 14)

#-# parameters:
#-#  Model                   Erdos
#-#  Number_of_nodes         600
#-#  Parameter_of_network    0.0325
#-#  Distribution            exponential 0.1
#-#  Immunity_update         1.5 0.8
#-#  N_graph                 5
#-#  N_initial               1
#-#  Number_of_simulations   5
## Averages of all simulations (peak, t_peak, t_half)
## 0% 'Initial'          (403.5, 10, 9)
## 20% 'Weights'         (245.3, 15, 10)
## 20% 'Weighted B.C.'   (263.0, 17, 8)
## 20% 'Weighted C.C.'   (311.1, 11, 9)
## 20% 'Unweighted B.C.' (382.5, 11, 9)
## 20% 'Unweighted C.C.' (348.7, 11, 9)
## 30% 'Weights'         (175.6, 22, 10)
## 30% 'Weighted B.C.'   (176.9, 22, 10)
## 30% 'Weighted C.C.'   (301.4, 12, 9)
## 30% 'Unweighted B.C.' (338.0, 12, 9)
## 30% 'Unweighted C.C.' (324.4, 13, 8)
## 40% 'Weights'         (60.6, 32, 15)
## 40% 'Weighted B.C.'   (77.6, 30, 13)
## 40% 'Weighted C.C.'   (258.6, 14, 10)
## 40% 'Unweighted B.C.' (300.0, 14, 9)
## 40% 'Unweighted C.C.' (311.4, 13, 9)
## 50% 'Weights'         (9.6, 35, 43)
## 50% 'Weighted B.C.'   (12.4, 46, 25)
## 50% 'Weighted C.C.'   (193.1, 16, 9)
## 50% 'Unweighted B.C.' (196.7, 17, 10)
## 50% 'Unweighted C.C.' (228.4, 15, 10)
## 80% 'Weights'         (1.0, 0, 3)
## 80% 'Weighted B.C.'   (1.0, 0, 5)
## 80% 'Weighted C.C.'   (20.9, 28, 17)
## 80% 'Unweighted B.C.' (29.1, 33, 14)
## 80% 'Unweighted C.C.' (59.7, 24, 13)
## 90% 'Weights'         (1.0, 0, 3)
## 90% 'Weighted B.C.'   (1.0, 0, 3)
## 90% 'Weighted C.C.'   (1.6, 23, 17)
## 90% 'Unweighted B.C.' (1.2, 2, 12)
## 90% 'Unweighted C.C.' (5.8, 23, 35)

#-# parameters:
#-#  Model                   Barabasi
#-#  Number_of_nodes         600
#-#  Parameter_of_network    20
#-#  Distribution            exponential 0.1
#-#  Immunity_update         1.5 0.8
#-#  N_graph                 5
#-#  N_initial               1
#-#  Number_of_simulations   5
## Averages of all simulations (peak, t_peak, t_half)
## 0% 'Initial'          (479.1, 6, 11)
## 20% 'Weights'         (415.5, 8, 10)
## 20% 'Weighted B.C.'   (357.1, 9, 10)
## 20% 'Weighted C.C.'   (469.6, 7, 10)
## 20% 'Unweighted B.C.' (455.0, 7, 10)
## 20% 'Unweighted C.C.' (468.8, 7, 10)
## 30% 'Weights'         (313.8, 11, 9)
## 30% 'Weighted B.C.'   (277.4, 11, 9)
## 30% 'Weighted C.C.'   (455.4, 7, 10)
## 30% 'Unweighted B.C.' (415.4, 9, 9)
## 30% 'Unweighted C.C.' (453.7, 8, 10)
## 40% 'Weights'         (283.7, 14, 9)
## 40% 'Weighted B.C.'   (241.3, 13, 9)
## 40% 'Weighted C.C.'   (351.0, 8, 10)
## 40% 'Unweighted B.C.' (424.1, 9, 9)
## 40% 'Unweighted C.C.' (335.5, 9, 9)
```

```
## 50% 'Weights'         (183.5, 17, 10)
## 50% 'Weighted B.C.'   (178.9, 16, 11)
## 50% 'Weighted C.C.'   (316.6, 9, 10)
## 50% 'Unweighted B.C.' (401.4, 9, 9)
## 50% 'Unweighted C.C.' (395.5, 9, 9)
## 80% 'Weights'         (1.0, 0, 3)
## 80% 'Weighted B.C.'   (2.5, 41, 26)
## 80% 'Weighted C.C.'   (135.4, 15, 10)
## 80% 'Unweighted B.C.' (172.8, 17, 11)
## 80% 'Unweighted C.C.' (177.0, 16, 9)
## 90% 'Weights'         (1.0, 0, 3)
## 90% 'Weighted B.C.'   (1.0, 0, 6)
## 90% 'Weighted C.C.'   (50.3, 22, 12)
## 90% 'Unweighted B.C.' (42.0, 25, 13)
## 90% 'Unweighted C.C.' (62.4, 22, 14)

#-# parameters:
#-#  Model                    Erdos
#-#  Number_of_nodes          600
#-#  Parameter_of_network     0.0625
#-#  Distribution             exponential 0.1
#-#  Immunity_update          1.5 0.8
#-#  N_graph                  5
#-#  N_initial                1
#-#  Number_of_simulations    5
## Averages of all simulations (peak, t_peak, t_half)
## 0% 'Initial'          (487.7, 6, 11)
## 20% 'Weights'         (359.2, 10, 9)
## 20% 'Weighted B.C.'   (421.4, 10, 9)
## 20% 'Weighted C.C.'   (419.1, 7, 10)
## 20% 'Unweighted B.C.' (467.2, 7, 10)
## 20% 'Unweighted C.C.' (469.4, 7, 10)
## 30% 'Weights'         (326.3, 13, 8)
## 30% 'Weighted B.C.'   (335.5, 12, 8)
## 30% 'Weighted C.C.'   (411.3, 8, 10)
## 30% 'Unweighted B.C.' (456.1, 7, 10)
## 30% 'Unweighted C.C.' (459.0, 7, 10)
## 40% 'Weights'         (238.8, 17, 9)
## 40% 'Weighted B.C.'   (242.3, 17, 8)
## 40% 'Weighted C.C.'   (344.4, 8, 10)
## 40% 'Unweighted B.C.' (439.1, 8, 10)
## 40% 'Unweighted C.C.' (376.2, 8, 10)
## 50% 'Weights'         (92.0, 24, 10)
## 50% 'Weighted B.C.'   (139.9, 23, 10)
## 50% 'Weighted C.C.'   (327.4, 9, 11)
## 50% 'Unweighted B.C.' (366.6, 10, 8)
## 50% 'Unweighted C.C.' (370.6, 9, 9)
## 80% 'Weights'         (1.0, 0, 4)
## 80% 'Weighted B.C.'   (1.0, 0, 3)
## 80% 'Weighted C.C.'   (108.7, 15, 10)
## 80% 'Unweighted B.C.' (154.8, 19, 10)
## 80% 'Unweighted C.C.' (173.6, 13, 10)
## 90% 'Weights'         (1.0, 0, 3)
## 90% 'Weighted B.C.'   (1.0, 0, 14)
## 90% 'Weighted C.C.'   (41.0, 19, 9)
## 90% 'Unweighted B.C.' (10.7, 32, 18)
## 90% 'Unweighted C.C.' (39.4, 21, 12)

#-# parameters:
#-#  Model                    Barabasi
#-#  Number_of_nodes          600
#-#  Parameter_of_network     30
#-#  Distribution             exponential 0.1
#-#  Immunity_update          1.5 0.8
#-#  N_graph                  5
#-#  N_initial                1
#-#  Number_of_simulations    5
## Averages of all simulations (peak, t_peak, t_half)
## 0% 'Initial'          (473.9, 4, 13)
## 20% 'Weights'         (443.0, 7, 10)
## 20% 'Weighted B.C.'   (440.3, 7, 10)
## 20% 'Weighted C.C.'   (497.3, 5, 12)
## 20% 'Unweighted B.C.' (463.6, 5, 12)
```

```
## 20% 'Unweighted C.C.' (487.9, 5, 12)
## 30% 'Weights'         (384.2, 9, 9)
## 30% 'Weighted B.C.'   (384.8, 8, 10)
## 30% 'Weighted C.C.'   (457.2, 6, 11)
## 30% 'Unweighted B.C.' (473.2, 6, 10)
## 30% 'Unweighted C.C.' (435.2, 6, 11)
## 40% 'Weights'         (329.8, 10, 9)
## 40% 'Weighted B.C.'   (324.4, 11, 9)
## 40% 'Weighted C.C.'   (469.3, 6, 11)
## 40% 'Unweighted B.C.' (434.9, 7, 10)
## 40% 'Unweighted C.C.' (444.4, 6, 11)
## 50% 'Weights'         (216.9, 15, 9)
## 50% 'Weighted B.C.'   (241.8, 14, 9)
## 50% 'Weighted C.C.'   (428.6, 7, 10)
## 50% 'Unweighted B.C.' (435.6, 9, 9)
## 50% 'Unweighted C.C.' (440.2, 7, 10)
## 80% 'Weights'         (1.0, 0, 5)
## 80% 'Weighted B.C.'   (28.5, 26, 19)
## 80% 'Weighted C.C.'   (250.0, 11, 9)
## 80% 'Unweighted B.C.' (172.6, 13, 11)
## 80% 'Unweighted C.C.' (205.7, 10, 9)
## 90% 'Weights'         (1.0, 0, 2)
## 90% 'Weighted B.C.'   (1.0, 0, 17)
## 90% 'Weighted C.C.'   (81.9, 15, 11)
## 90% 'Unweighted B.C.' (55.8, 20, 12)
## 90% 'Unweighted C.C.' (113.8, 16, 10)

#-# parameters:
#-#  Model                  Erdos
#-#  Number_of_nodes        600
#-#  Parameter_of_network   0.09
#-#  Distribution           exponential 0.1
#-#  Immunity_update        1.5 0.8
#-#  N_graph                5
#-#  N_initial              1
#-#  Number_of_simulations  5
## Averages of all simulations (peak, t_peak, t_half)
## 0% 'Initial'          (497.2, 5, 12)
## 20% 'Weights'         (463.5, 7, 10)
## 20% 'Weighted B.C.'   (443.8, 7, 10)
## 20% 'Weighted C.C.'   (463.8, 5, 12)
## 20% 'Unweighted B.C.' (491.3, 5, 12)
## 20% 'Unweighted C.C.' (493.2, 5, 12)
## 30% 'Weights'         (399.3, 9, 9)
## 30% 'Weighted B.C.'   (413.4, 9, 9)
## 30% 'Weighted C.C.'   (457.0, 6, 11)
## 30% 'Unweighted B.C.' (455.9, 6, 11)
## 30% 'Unweighted C.C.' (476.2, 6, 11)
## 40% 'Weights'         (358.4, 12, 8)
## 40% 'Weighted B.C.'   (329.7, 12, 8)
## 40% 'Weighted C.C.'   (420.1, 7, 10)
## 40% 'Unweighted B.C.' (462.6, 7, 10)
## 40% 'Unweighted C.C.' (463.6, 7, 10)
## 50% 'Weights'         (230.0, 17, 9)
## 50% 'Weighted B.C.'   (229.6, 17, 9)
## 50% 'Weighted C.C.'   (369.8, 8, 10)
## 50% 'Unweighted B.C.' (414.9, 7, 10)
## 50% 'Unweighted C.C.' (433.6, 7, 10)
## 80% 'Weights'         (1.0, 0, 8)
## 80% 'Weighted B.C.'   (8.9, 52, 20)
## 80% 'Weighted C.C.'   (198.8, 10, 10)
## 80% 'Unweighted B.C.' (257.6, 14, 8)
## 80% 'Unweighted C.C.' (199.7, 11, 9)
## 90% 'Weights'         (1.0, 0, 4)
## 90% 'Weighted B.C.'   (1.2, 2, 6)
## 90% 'Weighted C.C.'   (64.6, 14, 10)
## 90% 'Unweighted B.C.' (52.2, 26, 17)
## 90% 'Unweighted C.C.' (87.3, 15, 9)

#-# parameters:
#-#  Model                  Barabasi
#-#  Number_of_nodes        600
#-#  Parameter_of_network   50
```

```
#-#  Distribution            exponential 0.1
#-#  Immunity_update         1.5 0.8
#-#  N_graph                 5
#-#  N_initial               1
#-#  Number_of_simulations   5
## Averages of all simulations (peak, t_peak, t_half)
## 0% 'Initial'          (503.2, 4, 13)
## 20% 'Weights'         (480.4, 6, 11)
## 20% 'Weighted B.C.'   (488.4, 5, 11)
## 20% 'Weighted C.C.'   (514.4, 4, 13)
## 20% 'Unweighted B.C.' (489.8, 5, 12)
## 20% 'Unweighted C.C.' (487.0, 4, 13)
## 30% 'Weights'         (459.3, 7, 10)
## 30% 'Weighted B.C.'   (446.6, 7, 10)
## 30% 'Weighted C.C.'   (497.2, 4, 13)
## 30% 'Unweighted B.C.' (493.8, 5, 12)
## 30% 'Unweighted C.C.' (471.2, 4, 13)
## 40% 'Weights'         (442.6, 8, 9)
## 40% 'Weighted B.C.'   (413.8, 8, 9)
## 40% 'Weighted C.C.'   (493.5, 5, 12)
## 40% 'Unweighted B.C.' (484.6, 5, 12)
## 40% 'Unweighted C.C.' (425.2, 5, 12)
## 50% 'Weights'         (369.0, 10, 9)
## 50% 'Weighted B.C.'   (316.4, 9, 10)
## 50% 'Weighted C.C.'   (435.8, 5, 12)
## 50% 'Unweighted B.C.' (446.2, 6, 11)
## 50% 'Unweighted C.C.' (427.2, 5, 12)
## 80% 'Weights'         (2.2, 53, 19)
## 80% 'Weighted B.C.'   (120.2, 16, 11)
## 80% 'Weighted C.C.'   (289.4, 10, 9)
## 80% 'Unweighted B.C.' (162.3, 12, 11)
## 80% 'Unweighted C.C.' (235.6, 7, 10)
## 90% 'Weights'         (1.0, 0, 3)
## 90% 'Weighted B.C.'   (28.0, 29, 16)
## 90% 'Weighted C.C.'   (174.2, 10, 9)
## 90% 'Unweighted B.C.' (52.9, 9, 16)
## 90% 'Unweighted C.C.' (138.8, 9, 9)

#-# parameters:
#-#  Model                   Erdos
#-#  Number_of_nodes         600
#-#  Parameter_of_network    0.15
#-#  Distribution            exponential 0.1
#-#  Immunity_update         1.5 0.8
#-#  N_graph                 5
#-#  N_initial               1
#-#  Number_of_simulations   5
## Averages of all simulations (peak, t_peak, t_half)
## 0% 'Initial'          (516.5, 4, 14)
## 20% 'Weights'         (498.0, 5, 12)
## 20% 'Weighted B.C.'   (497.0, 5, 12)
## 20% 'Weighted C.C.'   (505.8, 4, 13)
## 20% 'Unweighted B.C.' (505.6, 4, 13)
## 20% 'Unweighted C.C.' (514.2, 4, 13)
## 30% 'Weights'         (475.5, 7, 10)
## 30% 'Weighted B.C.'   (475.7, 7, 10)
## 30% 'Weighted C.C.'   (461.5, 5, 12)
## 30% 'Unweighted B.C.' (496.8, 4, 13)
## 30% 'Unweighted C.C.' (499.7, 4, 13)
## 40% 'Weights'         (432.7, 9, 9)
## 40% 'Weighted B.C.'   (442.9, 8, 10)
## 40% 'Weighted C.C.'   (427.1, 6, 11)
## 40% 'Unweighted B.C.' (495.5, 5, 12)
## 40% 'Unweighted C.C.' (472.5, 5, 12)
## 50% 'Weights'         (375.2, 11, 9)
## 50% 'Weighted B.C.'   (394.9, 10, 9)
## 50% 'Weighted C.C.'   (407.3, 6, 11)
## 50% 'Unweighted B.C.' (488.4, 5, 12)
## 50% 'Unweighted C.C.' (410.8, 5, 12)
## 80% 'Weights'         (1.2, 4, 12)
## 80% 'Weighted B.C.'   (100.6, 19, 10)
## 80% 'Weighted C.C.'   (237.6, 8, 10)
## 80% 'Unweighted B.C.' (337.6, 9, 9)
```

```
## 80% 'Unweighted C.C.' (223.2, 8, 10)
## 90% 'Weights'         (1.0, 0, 4)
## 90% 'Weighted B.C.'   (32.3, 22, 10)
## 90% 'Weighted C.C.'   (97.4, 10, 10)
## 90% 'Unweighted B.C.' (242.2, 14, 10)
## 90% 'Unweighted C.C.' (107.8, 9, 13)

#-# parameters:
#-#  Model                   Barabasi
#-#  Number_of_nodes         300
#-#  Parameter_of_network    20
#-#  Distribution            exponential 0.1
#-#  Immunity_update         1.5 0.8
#-#  N_graph                 5
#-#  N_initial               1
#-#  Number_of_simulations   5
## Averages of all simulations (peak, t_peak, t_half)
## 0% 'Initial'          (241.0, 5, 11)
## 20% 'Weights'         (200.8, 9, 9)
## 20% 'Weighted B.C.'   (186.0, 9, 9)
## 20% 'Weighted C.C.'   (228.7, 6, 10)
## 20% 'Unweighted B.C.' (225.7, 7, 10)
## 20% 'Unweighted C.C.' (227.8, 7, 10)
## 30% 'Weights'         (146.9, 10, 9)
## 30% 'Weighted B.C.'   (149.6, 11, 8)
## 30% 'Weighted C.C.'   (214.2, 7, 10)
## 30% 'Unweighted B.C.' (224.6, 7, 9)
## 30% 'Unweighted C.C.' (210.5, 7, 10)
## 40% 'Weights'         (125.1, 13, 10)
## 40% 'Weighted B.C.'   (121.9, 13, 9)
## 40% 'Weighted C.C.'   (213.3, 8, 9)
## 40% 'Unweighted B.C.' (185.2, 8, 9)
## 40% 'Unweighted C.C.' (209.8, 8, 9)
## 50% 'Weights'         (70.1, 18, 10)
## 50% 'Weighted B.C.'   (74.0, 15, 12)
## 50% 'Weighted C.C.'   (190.0, 9, 9)
## 50% 'Unweighted B.C.' (175.2, 9, 9)
## 50% 'Unweighted C.C.' (187.6, 8, 9)
## 80% 'Weights'         (1.0, 0, 3)
## 80% 'Weighted B.C.'   (4.1, 22, 16)
## 80% 'Weighted C.C.'   (66.0, 15, 10)
## 80% 'Unweighted B.C.' (80.8, 16, 11)
## 80% 'Unweighted C.C.' (85.7, 14, 10)
## 90% 'Weights'         (1.0, 0, 3)
## 90% 'Weighted B.C.'   (1.0, 0, 17)
## 90% 'Weighted C.C.'   (15.5, 18, 12)
## 90% 'Unweighted B.C.' (16.4, 15, 14)
## 90% 'Unweighted C.C.' (21.6, 22, 11)

#-# parameters:
#-#  Model                   Erdos
#-#  Number_of_nodes         300
#-#  Parameter_of_network    0.125
#-#  Distribution            exponential 0.1
#-#  Immunity_update         1.5 0.8
#-#  N_graph                 5
#-#  N_initial               1
#-#  Number_of_simulations   5
## Averages of all simulations (peak, t_peak, t_half)
## 0% 'Initial'          (242.3, 5, 11)
## 20% 'Weights'         (200.4, 9, 9)
## 20% 'Weighted B.C.'   (210.5, 9, 9)
## 20% 'Weighted C.C.'   (225.4, 7, 10)
## 20% 'Unweighted B.C.' (226.0, 6, 10)
## 20% 'Unweighted C.C.' (220.0, 6, 11)
## 30% 'Weights'         (153.0, 12, 8)
## 30% 'Weighted B.C.'   (143.9, 12, 8)
## 30% 'Weighted C.C.'   (215.0, 7, 10)
## 30% 'Unweighted B.C.' (228.0, 7, 10)
## 30% 'Unweighted C.C.' (210.0, 7, 9)
## 40% 'Weights'         (127.6, 15, 9)
## 40% 'Weighted B.C.'   (136.4, 16, 8)
## 40% 'Weighted C.C.'   (176.1, 8, 9)
```

```
## 40% 'Unweighted B.C.' (219.8, 8, 9)
## 40% 'Unweighted C.C.' (206.6, 8, 9)
## 50% 'Weights'         (64.3, 23, 10)
## 50% 'Weighted B.C.'   (75.0, 22, 10)
## 50% 'Weighted C.C.'   (143.2, 10, 8)
## 50% 'Unweighted B.C.' (199.8, 9, 9)
## 50% 'Unweighted C.C.' (150.0, 9, 10)
## 80% 'Weights'         (1.0, 0, 5)
## 80% 'Weighted B.C.'   (1.0, 0, 10)
## 80% 'Weighted C.C.'   (53.4, 14, 9)
## 80% 'Unweighted B.C.' (65.8, 18, 10)
## 80% 'Unweighted C.C.' (82.6, 11, 9)
## 90% 'Weights'         (1.0, 0, 2)
## 90% 'Weighted B.C.'   (1.0, 0, 4)
## 90% 'Weighted C.C.'   (13.0, 19, 10)
## 90% 'Unweighted B.C.' (9.2, 32, 27)
## 90% 'Unweighted C.C.' (25.2, 19, 8)

#-# parameters:
#-#  Model                   Barabasi
#-#  Number_of_nodes         300
#-#  Parameter_of_network    20
#-#  Distribution            normal 0.1 0.05
#-#  Immunity_update         1.5 0.8
#-#  N_graph                 5
#-#  N_initial               1
#-#  Number_of_simulations   5
## Averages of all simulations (peak, t_peak, t_half)
## 0% 'Initial'          (239.0, 5, 11)
## 20% 'Weights'         (224.2, 6, 10)
## 20% 'Weighted B.C.'   (215.8, 7, 10)
## 20% 'Weighted C.C.'   (234.9, 6, 11)
## 20% 'Unweighted B.C.' (229.2, 6, 11)
## 20% 'Unweighted C.C.' (232.2, 6, 10)
## 30% 'Weights'         (200.7, 8, 9)
## 30% 'Weighted B.C.'   (205.8, 8, 9)
## 30% 'Weighted C.C.'   (223.6, 7, 10)
## 30% 'Unweighted B.C.' (218.8, 8, 9)
## 30% 'Unweighted C.C.' (205.5, 6, 11)
## 40% 'Weights'         (172.4, 8, 10)
## 40% 'Weighted B.C.'   (151.0, 9, 9)
## 40% 'Weighted C.C.'   (216.8, 7, 10)
## 40% 'Unweighted B.C.' (210.5, 8, 10)
## 40% 'Unweighted C.C.' (199.4, 7, 10)
## 50% 'Weights'         (147.7, 11, 9)
## 50% 'Weighted B.C.'   (160.5, 12, 8)
## 50% 'Weighted C.C.'   (183.9, 8, 9)
## 50% 'Unweighted B.C.' (182.6, 10, 9)
## 50% 'Unweighted C.C.' (187.9, 8, 9)
## 80% 'Weights'         (10.9, 26, 23)
## 80% 'Weighted B.C.'   (16.6, 25, 13)
## 80% 'Weighted C.C.'   (74.0, 15, 9)
## 80% 'Unweighted B.C.' (75.2, 16, 10)
## 80% 'Unweighted C.C.' (86.2, 14, 8)
## 90% 'Weights'         (1.0, 0, 9)
## 90% 'Weighted B.C.'   (1.1, 13, 15)
## 90% 'Weighted C.C.'   (16.8, 20, 10)
## 90% 'Unweighted B.C.' (14.9, 13, 19)
## 90% 'Unweighted C.C.' (22.6, 22, 9)

#-# parameters:
#-#  Model                   Erdos
#-#  Number_of_nodes         300
#-#  Parameter_of_network    0.125
#-#  Distribution            normal 0.1 0.05
#-#  Immunity_update         1.5 0.8
#-#  N_graph                 5
#-#  N_initial               1
#-#  Number_of_simulations   5
## Averages of all simulations (peak, t_peak, t_half)
## 0% 'Initial'          (249.3, 5, 11)
## 20% 'Weights'         (219.8, 7, 9)
## 20% 'Weighted B.C.'   (224.0, 7, 10)
```

```
## 20% 'Weighted C.C.'   (216.9, 6, 11)
## 20% 'Unweighted B.C.' (217.3, 6, 11)
## 20% 'Unweighted C.C.' (235.4, 6, 10)
## 30% 'Weights'         (216.3, 8, 9)
## 30% 'Weighted B.C.'   (219.7, 8, 9)
## 30% 'Weighted C.C.'   (219.2, 7, 10)
## 30% 'Unweighted B.C.' (209.1, 7, 10)
## 30% 'Unweighted C.C.' (181.8, 7, 10)
## 40% 'Weights'         (182.3, 10, 9)
## 40% 'Weighted B.C.'   (190.8, 9, 9)
## 40% 'Weighted C.C.'   (215.2, 8, 10)
## 40% 'Unweighted B.C.' (218.8, 8, 9)
## 40% 'Unweighted C.C.' (221.4, 7, 10)
## 50% 'Weights'         (173.6, 12, 9)
## 50% 'Weighted B.C.'   (158.1, 11, 10)
## 50% 'Weighted C.C.'   (175.2, 8, 10)
## 50% 'Unweighted B.C.' (186.4, 8, 9)
## 50% 'Unweighted C.C.' (168.8, 8, 9)
## 80% 'Weights'         (2.6, 21, 22)
## 80% 'Weighted B.C.'   (9.7, 41, 23)
## 80% 'Weighted C.C.'   (73.6, 13, 9)
## 80% 'Unweighted B.C.' (97.8, 15, 10)
## 80% 'Unweighted C.C.' (77.2, 12, 9)
## 90% 'Weights'         (1.0, 0, 6)
## 90% 'Weighted B.C.'   (1.0, 0, 9)
## 90% 'Weighted C.C.'   (13.8, 22, 8)
## 90% 'Unweighted B.C.' (15.8, 27, 16)
## 90% 'Unweighted C.C.' (24.5, 18, 9)

#-# parameters:
#-#  Model                   Barabasi
#-#  Number_of_nodes         300
#-#  Parameter_of_network    20
#-#  Distribution            uniform 0.05 0.15
#-#  Immunity_update         1.5 0.8
#-#  N_graph                 5
#-#  N_initial               1
#-#  Number_of_simulations   5
## Averages of all simulations (peak, t_peak, t_half)
## 0% 'Initial'          (240.3, 5, 11)
## 20% 'Weights'         (217.7, 6, 10)
## 20% 'Weighted B.C.'   (210.4, 6, 10)
## 20% 'Weighted C.C.'   (224.0, 6, 10)
## 20% 'Unweighted B.C.' (225.7, 7, 10)
## 20% 'Unweighted C.C.' (233.1, 6, 10)
## 30% 'Weights'         (210.0, 8, 9)
## 30% 'Weighted B.C.'   (211.9, 7, 9)
## 30% 'Weighted C.C.'   (229.8, 7, 10)
## 30% 'Unweighted B.C.' (214.7, 8, 9)
## 30% 'Unweighted C.C.' (224.0, 7, 9)
## 40% 'Weights'         (187.2, 8, 9)
## 40% 'Weighted B.C.'   (198.2, 8, 9)
## 40% 'Weighted C.C.'   (200.2, 7, 10)
## 40% 'Unweighted B.C.' (209.5, 8, 9)
## 40% 'Unweighted C.C.' (206.6, 7, 10)
## 50% 'Weights'         (174.5, 10, 9)
## 50% 'Weighted B.C.'   (168.5, 10, 9)
## 50% 'Weighted C.C.'   (185.4, 9, 9)
## 50% 'Unweighted B.C.' (201.2, 8, 10)
## 50% 'Unweighted C.C.' (196.1, 8, 9)
## 80% 'Weights'         (27.1, 23, 14)
## 80% 'Weighted B.C.'   (43.2, 15, 11)
## 80% 'Weighted C.C.'   (65.2, 14, 9)
## 80% 'Unweighted B.C.' (77.4, 15, 10)
## 80% 'Unweighted C.C.' (81.1, 14, 8)
## 90% 'Weights'         (1.9, 29, 18)
## 90% 'Weighted B.C.'   (4.5, 17, 16)
## 90% 'Weighted C.C.'   (25.6, 20, 12)
## 90% 'Unweighted B.C.' (14.0, 21, 18)
## 90% 'Unweighted C.C.' (23.4, 22, 11)

#-# parameters:
#-#  Model                   Erdos
```

```
#-#  Number_of_nodes         300
#-#  Parameter_of_network    0.125
#-#  Distribution            uniform 0.05 0.15
#-#  Immunity_update         1.5 0.8
#-#  N_graph                 5
#-#  N_initial               1
#-#  Number_of_simulations   5
## Averages of all simulations (peak, t_peak, t_half)
## 0% 'Initial'          (243.6, 6, 10)
## 20% 'Weights'         (232.8, 7, 9)
## 20% 'Weighted B.C.'   (230.0, 7, 10)
## 20% 'Weighted C.C.'   (235.9, 6, 10)
## 20% 'Unweighted B.C.' (231.6, 6, 10)
## 20% 'Unweighted C.C.' (233.1, 6, 11)
## 30% 'Weights'         (225.9, 8, 9)
## 30% 'Weighted B.C.'   (217.0, 8, 9)
## 30% 'Weighted C.C.'   (217.6, 7, 9)
## 30% 'Unweighted B.C.' (220.9, 7, 10)
## 30% 'Unweighted C.C.' (221.8, 6, 11)
## 40% 'Weights'         (209.9, 9, 9)
## 40% 'Weighted B.C.'   (211.8, 9, 9)
## 40% 'Weighted C.C.'   (197.9, 8, 9)
## 40% 'Unweighted B.C.' (199.2, 8, 9)
## 40% 'Unweighted C.C.' (207.5, 8, 9)
## 50% 'Weights'         (179.2, 10, 9)
## 50% 'Weighted B.C.'   (175.3, 10, 9)
## 50% 'Weighted C.C.'   (171.7, 9, 9)
## 50% 'Unweighted B.C.' (201.3, 9, 9)
## 50% 'Unweighted C.C.' (187.4, 8, 9)
## 80% 'Weights'         (49.8, 28, 14)
## 80% 'Weighted B.C.'   (39.4, 28, 12)
## 80% 'Weighted C.C.'   (77.9, 12, 10)
## 80% 'Unweighted B.C.' (60.0, 17, 10)
## 80% 'Unweighted C.C.' (46.5, 12, 9)
## 90% 'Weights'         (1.2, 25, 11)
## 90% 'Weighted B.C.'   (2.4, 26, 8)
## 90% 'Weighted C.C.'   (21.5, 16, 12)
## 90% 'Unweighted B.C.' (13.9, 26, 17)
## 90% 'Unweighted C.C.' (23.0, 16, 10)

#-# parameters:
#-#  Model                   Barabasi
#-#  Number_of_nodes         300
#-#  Parameter_of_network    20
#-#  Distribution            exponential 0.1
#-#  Immunity_update         1.3 0.8
#-#  N_graph                 5
#-#  N_initial               1
#-#  Number_of_simulations   5
## Averages of all simulations (peak, t_peak, t_half)
## 0% 'Initial'          (240.2, 6, 15)
## 20% 'Weights'         (208.1, 8, 12)
## 20% 'Weighted B.C.'   (182.8, 8, 14)
## 20% 'Weighted C.C.'   (225.6, 7, 13)
## 20% 'Unweighted B.C.' (230.5, 6, 14)
## 20% 'Unweighted C.C.' (233.3, 7, 14)
## 30% 'Weights'         (151.4, 11, 11)
## 30% 'Weighted B.C.'   (145.6, 10, 11)
## 30% 'Weighted C.C.'   (218.4, 8, 13)
## 30% 'Unweighted B.C.' (214.0, 8, 12)
## 30% 'Unweighted C.C.' (219.4, 7, 13)
## 40% 'Weights'         (127.2, 14, 12)
## 40% 'Weighted B.C.'   (125.6, 13, 11)
## 40% 'Weighted C.C.'   (177.2, 9, 12)
## 40% 'Unweighted B.C.' (207.4, 10, 12)
## 40% 'Unweighted C.C.' (211.9, 8, 13)
## 50% 'Weights'         (90.8, 19, 12)
## 50% 'Weighted B.C.'   (78.0, 17, 12)
## 50% 'Weighted C.C.'   (180.8, 9, 12)
## 50% 'Unweighted B.C.' (186.2, 9, 13)
## 50% 'Unweighted C.C.' (191.8, 8, 13)
## 80% 'Weights'         (1.0, 0, 7)
## 80% 'Weighted B.C.'   (2.8, 28, 18)
```

```
## 80% 'Weighted C.C.'   (64.5, 15, 12)
## 80% 'Unweighted B.C.' (49.1, 17, 10)
## 80% 'Unweighted C.C.' (71.1, 14, 12)
## 90% 'Weights'         (1.0, 0, 3)
## 90% 'Weighted B.C.'   (1.0, 0, 8)
## 90% 'Weighted C.C.'   (16.0, 20, 17)
## 90% 'Unweighted B.C.' (19.8, 24, 13)
## 90% 'Unweighted C.C.' (31.9, 20, 16)

#-# parameters:
#-#  Model                    Erdos
#-#  Number_of_nodes          300
#-#  Parameter_of_network     0.125
#-#  Distribution             exponential 0.1
#-#  Immunity_update          1.3 0.8
#-#  N_graph                  5
#-#  N_initial                1
#-#  Number_of_simulations    5
## Averages of all simulations (peak, t_peak, t_half)
## 0% 'Initial'          (231.2, 6, 15)
## 20% 'Weights'         (217.0, 9, 12)
## 20% 'Weighted B.C.'   (190.1, 10, 11)
## 20% 'Weighted C.C.'   (222.2, 6, 14)
## 20% 'Unweighted B.C.' (235.6, 6, 15)
## 20% 'Unweighted C.C.' (227.2, 7, 14)
## 30% 'Weights'         (176.5, 12, 11)
## 30% 'Weighted B.C.'   (187.4, 11, 11)
## 30% 'Weighted C.C.'   (196.4, 8, 14)
## 30% 'Unweighted B.C.' (229.5, 7, 14)
## 30% 'Unweighted C.C.' (230.1, 7, 13)
## 40% 'Weights'         (135.7, 16, 11)
## 40% 'Weighted B.C.'   (146.2, 16, 10)
## 40% 'Weighted C.C.'   (187.3, 8, 13)
## 40% 'Unweighted B.C.' (216.8, 9, 12)
## 40% 'Unweighted C.C.' (200.6, 8, 13)
## 50% 'Weights'         (65.2, 23, 15)
## 50% 'Weighted B.C.'   (88.0, 20, 12)
## 50% 'Weighted C.C.'   (146.3, 9, 13)
## 50% 'Unweighted B.C.' (214.5, 9, 12)
## 50% 'Unweighted C.C.' (206.8, 8, 13)
## 80% 'Weights'         (1.0, 0, 3)
## 80% 'Weighted B.C.'   (1.0, 0, 7)
## 80% 'Weighted C.C.'   (35.3, 12, 11)
## 80% 'Unweighted B.C.' (94.7, 17, 10)
## 80% 'Unweighted C.C.' (63.9, 13, 11)
## 90% 'Weights'         (1.0, 0, 3)
## 90% 'Weighted B.C.'   (1.0, 0, 17)
## 90% 'Weighted C.C.'   (13.6, 25, 11)
## 90% 'Unweighted B.C.' (8.0, 27, 20)
## 90% 'Unweighted C.C.' (25.2, 21, 11)

#-# parameters:
#-#  Model                    Barabasi
#-#  Number_of_nodes          300
#-#  Parameter_of_network     20
#-#  Distribution             exponential 0.1
#-#  Immunity_update          1.4 0.8
#-#  N_graph                  5
#-#  N_initial                1
#-#  Number_of_simulations    5
## Averages of all simulations (peak, t_peak, t_half)
## 0% 'Initial'          (235.4, 6, 12)
## 20% 'Weights'         (191.1, 9, 10)
## 20% 'Weighted B.C.'   (196.2, 9, 10)
## 20% 'Weighted C.C.'   (225.3, 6, 12)
## 20% 'Unweighted B.C.' (230.7, 6, 12)
## 20% 'Unweighted C.C.' (229.2, 7, 11)
## 30% 'Weights'         (163.8, 10, 10)
## 30% 'Weighted B.C.'   (162.6, 11, 9)
## 30% 'Weighted C.C.'   (227.0, 7, 11)
## 30% 'Unweighted B.C.' (199.9, 8, 11)
## 30% 'Unweighted C.C.' (194.4, 7, 11)
## 40% 'Weights'         (85.6, 15, 9)
```

```
## 40% 'Weighted B.C.'   (123.0, 14, 9)
## 40% 'Weighted C.C.'   (187.0, 8, 12)
## 40% 'Unweighted B.C.' (203.9, 8, 10)
## 40% 'Unweighted C.C.' (216.4, 8, 10)
## 50% 'Weights'         (70.6, 20, 11)
## 50% 'Weighted B.C.'   (82.8, 16, 11)
## 50% 'Weighted C.C.'   (168.6, 9, 10)
## 50% 'Unweighted B.C.' (185.9, 10, 10)
## 50% 'Unweighted C.C.' (199.4, 8, 11)
## 80% 'Weights'         (1.0, 0, 9)
## 80% 'Weighted B.C.'   (2.1, 15, 35)
## 80% 'Weighted C.C.'   (60.3, 15, 9)
## 80% 'Unweighted B.C.' (51.9, 17, 10)
## 80% 'Unweighted C.C.' (75.4, 15, 9)
## 90% 'Weights'         (1.0, 0, 5)
## 90% 'Weighted B.C.'   (1.0, 0, 3)
## 90% 'Weighted C.C.'   (10.0, 21, 12)
## 90% 'Unweighted B.C.' (10.9, 21, 14)
## 90% 'Unweighted C.C.' (14.3, 25, 13)

#-# parameters:
#-#  Model                    Erdos
#-#  Number_of_nodes          300
#-#  Parameter_of_network     0.125
#-#  Distribution             exponential 0.1
#-#  Immunity_update          1.4 0.8
#-#  N_graph                  5
#-#  N_initial                1
#-#  Number_of_simulations    5
## Averages of all simulations (peak, t_peak, t_half)
## 0% 'Initial'          (243.4, 5, 13)
## 20% 'Weights'         (213.9, 9, 10)
## 20% 'Weighted B.C.'   (207.4, 9, 10)
## 20% 'Weighted C.C.'   (210.3, 7, 12)
## 20% 'Unweighted B.C.' (237.9, 6, 12)
## 20% 'Unweighted C.C.' (232.2, 7, 11)
## 30% 'Weights'         (165.5, 11, 10)
## 30% 'Weighted B.C.'   (183.2, 11, 10)
## 30% 'Weighted C.C.'   (217.8, 8, 11)
## 30% 'Unweighted B.C.' (228.8, 7, 11)
## 30% 'Unweighted C.C.' (208.9, 7, 11)
## 40% 'Weights'         (118.6, 14, 10)
## 40% 'Weighted B.C.'   (135.5, 14, 9)
## 40% 'Weighted C.C.'   (180.5, 8, 11)
## 40% 'Unweighted B.C.' (222.2, 8, 10)
## 40% 'Unweighted C.C.' (209.2, 8, 11)
## 50% 'Weights'         (61.8, 21, 12)
## 50% 'Weighted B.C.'   (67.6, 21, 11)
## 50% 'Weighted C.C.'   (167.7, 8, 11)
## 50% 'Unweighted B.C.' (188.9, 10, 9)
## 50% 'Unweighted C.C.' (203.9, 8, 11)
## 80% 'Weights'         (1.0, 0, 5)
## 80% 'Weighted B.C.'   (1.0, 0, 4)
## 80% 'Weighted C.C.'   (59.0, 13, 9)
## 80% 'Unweighted B.C.' (104.5, 19, 9)
## 80% 'Unweighted C.C.' (72.9, 14, 9)
## 90% 'Weights'         (1.0, 0, 4)
## 90% 'Weighted B.C.'   (1.0, 0, 12)
## 90% 'Weighted C.C.'   (16.0, 16, 11)
## 90% 'Unweighted B.C.' (7.7, 26, 27)
## 90% 'Unweighted C.C.' (26.6, 18, 9)

#-# parameters:
#-#  Model                    Barabasi
#-#  Number_of_nodes          300
#-#  Parameter_of_network     20
#-#  Distribution             exponential 0.1
#-#  Immunity_update          1.5 0.8
#-#  N_graph                  5
#-#  N_initial                1
#-#  Number_of_simulations    5
## Averages of all simulations (peak, t_peak, t_half)
## 0% 'Initial'          (241.0, 5, 11)
```

```
## 20% 'Weights'          (200.8, 9, 9)
## 20% 'Weighted B.C.'    (186.0, 9, 9)
## 20% 'Weighted C.C.'    (228.7, 6, 10)
## 20% 'Unweighted B.C.' (225.7, 7, 10)
## 20% 'Unweighted C.C.' (227.8, 7, 10)
## 30% 'Weights'          (146.9, 10, 9)
## 30% 'Weighted B.C.'    (149.6, 11, 8)
## 30% 'Weighted C.C.'    (214.2, 7, 10)
## 30% 'Unweighted B.C.' (224.6, 7, 9)
## 30% 'Unweighted C.C.' (210.5, 7, 10)
## 40% 'Weights'          (125.1, 13, 10)
## 40% 'Weighted B.C.'    (121.9, 13, 9)
## 40% 'Weighted C.C.'    (213.3, 8, 9)
## 40% 'Unweighted B.C.' (185.2, 8, 9)
## 40% 'Unweighted C.C.' (209.8, 8, 9)
## 50% 'Weights'          (70.1, 18, 10)
## 50% 'Weighted B.C.'    (74.0, 15, 12)
## 50% 'Weighted C.C.'    (190.0, 9, 9)
## 50% 'Unweighted B.C.' (175.2, 9, 9)
## 50% 'Unweighted C.C.' (187.6, 8, 9)
## 80% 'Weights'          (1.0, 0, 3)
## 80% 'Weighted B.C.'    (4.1, 22, 16)
## 80% 'Weighted C.C.'    (66.0, 15, 10)
## 80% 'Unweighted B.C.' (80.8, 16, 11)
## 80% 'Unweighted C.C.' (85.7, 14, 10)
## 90% 'Weights'          (1.0, 0, 3)
## 90% 'Weighted B.C.'    (1.0, 0, 17)
## 90% 'Weighted C.C.'    (15.5, 18, 12)
## 90% 'Unweighted B.C.' (16.4, 15, 14)
## 90% 'Unweighted C.C.' (21.6, 22, 11)

#-# parameters:
#-#  Model                  Erdos
#-#  Number_of_nodes        300
#-#  Parameter_of_network   0.125
#-#  Distribution           exponential 0.1
#-#  Immunity_update        1.5 0.8
#-#  N_graph                5
#-#  N_initial              1
#-#  Number_of_simulations  5
## Averages of all simulations (peak, t_peak, t_half)
## 0% 'Initial'           (242.3, 5, 11)
## 20% 'Weights'          (200.4, 9, 9)
## 20% 'Weighted B.C.'    (210.5, 9, 9)
## 20% 'Weighted C.C.'    (225.4, 7, 10)
## 20% 'Unweighted B.C.' (226.0, 6, 10)
## 20% 'Unweighted C.C.' (220.0, 6, 11)
## 30% 'Weights'          (153.0, 12, 8)
## 30% 'Weighted B.C.'    (143.9, 12, 8)
## 30% 'Weighted C.C.'    (215.0, 7, 10)
## 30% 'Unweighted B.C.' (228.0, 7, 10)
## 30% 'Unweighted C.C.' (210.0, 7, 9)
## 40% 'Weights'          (127.6, 15, 9)
## 40% 'Weighted B.C.'    (136.4, 16, 8)
## 40% 'Weighted C.C.'    (176.1, 8, 9)
## 40% 'Unweighted B.C.' (219.8, 8, 9)
## 40% 'Unweighted C.C.' (206.6, 8, 9)
## 50% 'Weights'          (64.3, 23, 10)
## 50% 'Weighted B.C.'    (75.0, 22, 10)
## 50% 'Weighted C.C.'    (143.2, 10, 8)
## 50% 'Unweighted B.C.' (199.8, 9, 9)
## 50% 'Unweighted C.C.' (150.0, 9, 10)
## 80% 'Weights'          (1.0, 0, 5)
## 80% 'Weighted B.C.'    (1.0, 0, 10)
## 80% 'Weighted C.C.'    (53.4, 14, 9)
## 80% 'Unweighted B.C.' (65.8, 18, 10)
## 80% 'Unweighted C.C.' (82.6, 11, 9)
## 90% 'Weights'          (1.0, 0, 2)
## 90% 'Weighted B.C.'    (1.0, 0, 4)
## 90% 'Weighted C.C.'    (13.0, 19, 10)
## 90% 'Unweighted B.C.' (9.2, 32, 27)
## 90% 'Unweighted C.C.' (25.2, 19, 8)
```

```
#-# parameters:
#-#  Model                   Barabasi
#-#  Number_of_nodes         300
#-#  Parameter_of_network    20
#-#  Distribution            exponential 0.1
#-#  Immunity_update         1.6 0.8
#-#  N_graph                 5
#-#  N_initial               1
#-#  Number_of_simulations   5
## Averages of all simulations (peak, t_peak, t_half)
## 0% 'Initial'          (237.3, 5, 10)
## 20% 'Weights'         (187.0, 9, 8)
## 20% 'Weighted B.C.'   (184.3, 8, 8)
## 20% 'Weighted C.C.'   (224.6, 6, 9)
## 20% 'Unweighted B.C.' (222.8, 7, 9)
## 20% 'Unweighted C.C.' (220.0, 6, 10)
## 30% 'Weights'         (159.9, 10, 9)
## 30% 'Weighted B.C.'   (159.8, 10, 8)
## 30% 'Weighted C.C.'   (224.6, 7, 8)
## 30% 'Unweighted B.C.' (199.3, 7, 9)
## 30% 'Unweighted C.C.' (224.6, 7, 9)
## 40% 'Weights'         (115.0, 13, 8)
## 40% 'Weighted B.C.'   (114.1, 13, 9)
## 40% 'Weighted C.C.'   (188.8, 8, 9)
## 40% 'Unweighted B.C.' (193.9, 8, 9)
## 40% 'Unweighted C.C.' (200.8, 8, 8)
## 50% 'Weights'         (50.3, 17, 12)
## 50% 'Weighted B.C.'   (76.9, 18, 11)
## 50% 'Weighted C.C.'   (181.6, 8, 8)
## 50% 'Unweighted B.C.' (147.7, 9, 9)
## 50% 'Unweighted C.C.' (183.5, 8, 9)
## 80% 'Weights'         (1.0, 0, 7)
## 80% 'Weighted B.C.'   (4.3, 21, 13)
## 80% 'Weighted C.C.'   (66.2, 13, 10)
## 80% 'Unweighted B.C.' (50.2, 15, 12)
## 80% 'Unweighted C.C.' (59.4, 14, 9)
## 90% 'Weights'         (1.0, 0, 5)
## 90% 'Weighted B.C.'   (1.0, 0, 7)
## 90% 'Weighted C.C.'   (24.5, 23, 10)
## 90% 'Unweighted B.C.' (10.1, 20, 16)
## 90% 'Unweighted C.C.' (18.1, 19, 11)

#-# parameters:
#-#  Model                   Erdos
#-#  Number_of_nodes         300
#-#  Parameter_of_network    0.125
#-#  Distribution            exponential 0.1
#-#  Immunity_update         1.6 0.8
#-#  N_graph                 5
#-#  N_initial               1
#-#  Number_of_simulations   5
## Averages of all simulations (peak, t_peak, t_half)
## 0% 'Initial'          (241.0, 6, 9)
## 20% 'Weights'         (207.8, 9, 8)
## 20% 'Weighted B.C.'   (205.4, 8, 9)
## 20% 'Weighted C.C.'   (223.9, 7, 9)
## 20% 'Unweighted B.C.' (225.3, 6, 9)
## 20% 'Unweighted C.C.' (236.0, 6, 9)
## 30% 'Weights'         (181.5, 11, 8)
## 30% 'Weighted B.C.'   (181.3, 11, 8)
## 30% 'Weighted C.C.'   (210.0, 7, 9)
## 30% 'Unweighted B.C.' (216.1, 7, 9)
## 30% 'Unweighted C.C.' (216.1, 7, 9)
## 40% 'Weights'         (118.4, 15, 8)
## 40% 'Weighted B.C.'   (122.2, 14, 9)
## 40% 'Weighted C.C.'   (191.2, 9, 8)
## 40% 'Unweighted B.C.' (209.0, 9, 8)
## 40% 'Unweighted C.C.' (216.2, 8, 8)
## 50% 'Weights'         (60.6, 23, 13)
## 50% 'Weighted B.C.'   (71.7, 21, 11)
## 50% 'Weighted C.C.'   (168.8, 8, 9)
## 50% 'Unweighted B.C.' (179.5, 9, 9)
## 50% 'Unweighted C.C.' (202.9, 8, 8)
```

```
## 80% 'Weights'          (1.0, 0, 3)
## 80% 'Weighted B.C.'    (1.0, 0, 16)
## 80% 'Weighted C.C.'    (49.7, 14, 7)
## 80% 'Unweighted B.C.' (79.1, 16, 10)
## 80% 'Unweighted C.C.' (77.7, 12, 9)
## 90% 'Weights'          (1.0, 0, 4)
## 90% 'Weighted B.C.'    (1.0, 0, 12)
## 90% 'Weighted C.C.'    (13.3, 19, 9)
## 90% 'Unweighted B.C.' (10.0, 29, 10)
## 90% 'Unweighted C.C.' (31.8, 14, 12)

#-# parameters:
#-#  Model                    Barabasi
#-#  Number_of_nodes          300
#-#  Parameter_of_network     20
#-#  Distribution             exponential 0.1
#-#  Immunity_update          1.7 0.8
#-#  N_graph                  5
#-#  N_initial                1
#-#  Number_of_simulations    5
## Averages of all simulations (peak, t_peak, t_half)
## 0% 'Initial'           (235.7, 6, 9)
## 20% 'Weights'          (182.6, 8, 8)
## 20% 'Weighted B.C.'    (193.5, 8, 8)
## 20% 'Weighted C.C.'    (221.3, 6, 10)
## 20% 'Unweighted B.C.' (222.0, 7, 8)
## 20% 'Unweighted C.C.' (230.5, 6, 9)
## 30% 'Weights'          (146.0, 10, 8)
## 30% 'Weighted B.C.'    (145.6, 11, 8)
## 30% 'Weighted C.C.'    (212.8, 7, 8)
## 30% 'Unweighted B.C.' (214.2, 7, 8)
## 30% 'Unweighted C.C.' (215.4, 7, 8)
## 40% 'Weights'          (111.3, 13, 8)
## 40% 'Weighted B.C.'    (92.4, 13, 8)
## 40% 'Weighted C.C.'    (188.3, 8, 8)
## 40% 'Unweighted B.C.' (194.2, 8, 8)
## 40% 'Unweighted C.C.' (200.6, 7, 8)
## 50% 'Weights'          (56.6, 16, 14)
## 50% 'Weighted B.C.'    (68.2, 17, 8)
## 50% 'Weighted C.C.'    (151.2, 9, 8)
## 50% 'Unweighted B.C.' (171.2, 10, 8)
## 50% 'Unweighted C.C.' (182.4, 8, 8)
## 80% 'Weights'          (1.0, 0, 8)
## 80% 'Weighted B.C.'    (3.1, 30, 12)
## 80% 'Weighted C.C.'    (73.2, 14, 9)
## 80% 'Unweighted B.C.' (53.3, 16, 10)
## 80% 'Unweighted C.C.' (66.1, 14, 8)
## 90% 'Weights'          (1.0, 0, 3)
## 90% 'Weighted B.C.'    (1.1, 8, 7)
## 90% 'Weighted C.C.'    (16.4, 23, 12)
## 90% 'Unweighted B.C.' (8.8, 17, 11)
## 90% 'Unweighted C.C.' (19.5, 27, 10)

#-# parameters:
#-#  Model                    Erdos
#-#  Number_of_nodes          300
#-#  Parameter_of_network     0.125
#-#  Distribution             exponential 0.1
#-#  Immunity_update          1.7 0.8
#-#  N_graph                  5
#-#  N_initial                1
#-#  Number_of_simulations    5
## Averages of all simulations (peak, t_peak, t_half)
## 0% 'Initial'           (240.2, 6, 9)
## 20% 'Weights'          (204.8, 9, 7)
## 20% 'Weighted B.C.'    (195.2, 9, 8)
## 20% 'Weighted C.C.'    (213.6, 7, 8)
## 20% 'Unweighted B.C.' (217.0, 6, 9)
## 20% 'Unweighted C.C.' (225.2, 6, 8)
## 30% 'Weights'          (163.0, 11, 8)
## 30% 'Weighted B.C.'    (175.8, 11, 7)
## 30% 'Weighted C.C.'    (201.0, 7, 9)
## 30% 'Unweighted B.C.' (223.6, 7, 8)
```

```
## 30% 'Unweighted C.C.' (226.9, 6, 9)
## 40% 'Weights'         (103.3, 15, 9)
## 40% 'Weighted B.C.'   (120.7, 15, 7)
## 40% 'Weighted C.C.'   (175.3, 7, 10)
## 40% 'Unweighted B.C.' (192.5, 8, 8)
## 40% 'Unweighted C.C.' (187.8, 7, 8)
## 50% 'Weights'         (48.7, 21, 12)
## 50% 'Weighted B.C.'   (51.7, 19, 10)
## 50% 'Weighted C.C.'   (123.7, 8, 9)
## 50% 'Unweighted B.C.' (185.2, 8, 8)
## 50% 'Unweighted C.C.' (184.2, 8, 8)
## 80% 'Weights'         (1.0, 0, 6)
## 80% 'Weighted B.C.'   (1.0, 0, 19)
## 80% 'Weighted C.C.'   (73.3, 13, 8)
## 80% 'Unweighted B.C.' (58.0, 15, 10)
## 80% 'Unweighted C.C.' (55.8, 15, 7)
## 90% 'Weights'         (1.0, 0, 5)
## 90% 'Weighted B.C.'   (1.0, 0, 5)
## 90% 'Weighted C.C.'   (20.4, 20, 8)
## 90% 'Unweighted B.C.' (12.4, 25, 21)
## 90% 'Unweighted C.C.' (17.4, 18, 10)

#-# parameters:
#-#  Model                   Barabasi
#-#  Number_of_nodes         300
#-#  Parameter_of_network    20
#-#  Distribution            exponential 0.1
#-#  Immunity_update         1.8 0.8
#-#  N_graph                 5
#-#  N_initial               1
#-#  Number_of_simulations   5
## Averages of all simulations (peak, t_peak, t_half)
## 0% 'Initial'          (227.6, 5, 9)
## 20% 'Weights'         (182.6, 8, 8)
## 20% 'Weighted B.C.'   (165.7, 8, 8)
## 20% 'Weighted C.C.'   (226.7, 7, 7)
## 20% 'Unweighted B.C.' (226.3, 6, 8)
## 20% 'Unweighted C.C.' (234.2, 6, 8)
## 30% 'Weights'         (140.3, 9, 9)
## 30% 'Weighted B.C.'   (158.2, 11, 7)
## 30% 'Weighted C.C.'   (203.1, 7, 8)
## 30% 'Unweighted B.C.' (190.7, 7, 8)
## 30% 'Unweighted C.C.' (212.5, 7, 7)
## 40% 'Weights'         (113.2, 14, 7)
## 40% 'Weighted B.C.'   (131.2, 11, 9)
## 40% 'Weighted C.C.'   (191.9, 8, 7)
## 40% 'Unweighted B.C.' (189.8, 8, 8)
## 40% 'Unweighted C.C.' (202.9, 7, 8)
## 50% 'Weights'         (70.0, 17, 10)
## 50% 'Weighted B.C.'   (60.8, 15, 9)
## 50% 'Weighted C.C.'   (192.4, 9, 7)
## 50% 'Unweighted B.C.' (166.2, 9, 8)
## 50% 'Unweighted C.C.' (194.9, 8, 8)
## 80% 'Weights'         (1.0, 0, 5)
## 80% 'Weighted B.C.'   (2.8, 28, 10)
## 80% 'Weighted C.C.'   (44.0, 15, 9)
## 80% 'Unweighted B.C.' (43.2, 15, 11)
## 80% 'Unweighted C.C.' (62.2, 15, 8)
## 90% 'Weights'         (1.0, 0, 5)
## 90% 'Weighted B.C.'   (1.0, 0, 8)
## 90% 'Weighted C.C.'   (17.4, 17, 15)
## 90% 'Unweighted B.C.' (11.4, 15, 16)
## 90% 'Unweighted C.C.' (20.6, 20, 14)

#-# parameters:
#-#  Model                   Erdos
#-#  Number_of_nodes         300
#-#  Parameter_of_network    0.125
#-#  Distribution            exponential 0.1
#-#  Immunity_update         1.8 0.8
#-#  N_graph                 5
#-#  N_initial               1
#-#  Number_of_simulations   5
```

```
## Averages of all simulations (peak, t_peak, t_half)
## 0% 'Initial'          (244.5, 5, 9)
## 20% 'Weights'         (196.1, 8, 7)
## 20% 'Weighted B.C.'   (203.4, 9, 7)
## 20% 'Weighted C.C.'   (211.2, 6, 8)
## 20% 'Unweighted B.C.' (222.6, 6, 9)
## 20% 'Unweighted C.C.' (227.4, 7, 8)
## 30% 'Weights'         (153.8, 11, 7)
## 30% 'Weighted B.C.'   (145.6, 11, 7)
## 30% 'Weighted C.C.'   (190.8, 7, 8)
## 30% 'Unweighted B.C.' (226.3, 7, 8)
## 30% 'Unweighted C.C.' (220.6, 7, 7)
## 40% 'Weights'         (99.6, 15, 8)
## 40% 'Weighted B.C.'   (123.1, 14, 8)
## 40% 'Weighted C.C.'   (166.2, 8, 8)
## 40% 'Unweighted B.C.' (205.3, 7, 8)
## 40% 'Unweighted C.C.' (202.1, 7, 8)
## 50% 'Weights'         (59.8, 21, 10)
## 50% 'Weighted B.C.'   (68.8, 20, 11)
## 50% 'Weighted C.C.'   (161.9, 8, 8)
## 50% 'Unweighted B.C.' (195.6, 8, 8)
## 50% 'Unweighted C.C.' (180.1, 8, 8)
## 80% 'Weights'         (1.0, 0, 3)
## 80% 'Weighted B.C.'   (1.0, 0, 25)
## 80% 'Weighted C.C.'   (53.2, 13, 10)
## 80% 'Unweighted B.C.' (78.2, 18, 8)
## 80% 'Unweighted C.C.' (75.4, 13, 8)
## 90% 'Weights'         (1.0, 0, 3)
## 90% 'Weighted B.C.'   (1.0, 0, 5)
## 90% 'Weighted C.C.'   (9.5, 12, 13)
## 90% 'Unweighted B.C.' (8.8, 28, 14)
## 90% 'Unweighted C.C.' (28.0, 16, 9)

#-# parameters:
#-#  Model                   Barabasi
#-#  Number_of_nodes         300
#-#  Parameter_of_network    20
#-#  Distribution            exponential 0.1
#-#  Immunity_update         1.9 0.8
#-#  N_graph                 5
#-#  N_initial               1
#-#  Number_of_simulations   5
## Averages of all simulations (peak, t_peak, t_half)
## 0% 'Initial'          (242.0, 5, 8)
## 20% 'Weights'         (164.9, 8, 7)
## 20% 'Weighted B.C.'   (183.3, 8, 7)
## 20% 'Weighted C.C.'   (226.2, 6, 8)
## 20% 'Unweighted B.C.' (222.9, 6, 8)
## 20% 'Unweighted C.C.' (212.0, 6, 8)
## 30% 'Weights'         (141.8, 10, 8)
## 30% 'Weighted B.C.'   (151.3, 10, 7)
## 30% 'Weighted C.C.'   (203.6, 7, 7)
## 30% 'Unweighted B.C.' (217.6, 7, 8)
## 30% 'Unweighted C.C.' (222.0, 6, 8)
## 40% 'Weights'         (102.3, 13, 8)
## 40% 'Weighted B.C.'   (105.1, 13, 9)
## 40% 'Weighted C.C.'   (151.7, 9, 7)
## 40% 'Unweighted B.C.' (175.5, 8, 8)
## 40% 'Unweighted C.C.' (192.6, 8, 7)
## 50% 'Weights'         (68.2, 18, 10)
## 50% 'Weighted B.C.'   (70.0, 16, 9)
## 50% 'Weighted C.C.'   (159.6, 8, 8)
## 50% 'Unweighted B.C.' (159.1, 9, 7)
## 50% 'Unweighted C.C.' (182.4, 8, 7)
## 80% 'Weights'         (1.0, 0, 2)
## 80% 'Weighted B.C.'   (4.4, 25, 19)
## 80% 'Weighted C.C.'   (75.4, 14, 8)
## 80% 'Unweighted B.C.' (56.4, 16, 9)
## 80% 'Unweighted C.C.' (67.4, 14, 8)
## 90% 'Weights'         (1.0, 0, 5)
## 90% 'Weighted B.C.'   (1.0, 0, 9)
## 90% 'Weighted C.C.'   (6.8, 25, 10)
## 90% 'Unweighted B.C.' (8.4, 16, 16)
```

```
## 90% 'Unweighted C.C.' (29.8, 21, 9)

#-# parameters:
#-#  Model                   Erdos
#-#  Number_of_nodes         300
#-#  Parameter_of_network    0.125
#-#  Distribution            exponential 0.1
#-#  Immunity_update         1.9 0.8
#-#  N_graph                 5
#-#  N_initial               1
#-#  Number_of_simulations   5
## Averages of all simulations (peak, t_peak, t_half)
## 0% 'Initial'          (240.7, 5, 8)
## 20% 'Weights'         (188.3, 9, 7)
## 20% 'Weighted B.C.'   (201.7, 9, 7)
## 20% 'Weighted C.C.'   (224.5, 6, 8)
## 20% 'Unweighted B.C.' (209.9, 6, 8)
## 20% 'Unweighted C.C.' (231.9, 6, 8)
## 30% 'Weights'         (150.2, 11, 8)
## 30% 'Weighted B.C.'   (167.8, 10, 7)
## 30% 'Weighted C.C.'   (210.2, 7, 8)
## 30% 'Unweighted B.C.' (212.2, 7, 8)
## 30% 'Unweighted C.C.' (222.8, 6, 8)
## 40% 'Weights'         (122.9, 15, 8)
## 40% 'Weighted B.C.'   (116.0, 15, 8)
## 40% 'Weighted C.C.'   (171.4, 7, 8)
## 40% 'Unweighted B.C.' (213.1, 8, 7)
## 40% 'Unweighted C.C.' (192.8, 8, 7)
## 50% 'Weights'         (57.2, 22, 11)
## 50% 'Weighted B.C.'   (74.2, 20, 9)
## 50% 'Weighted C.C.'   (146.5, 8, 8)
## 50% 'Unweighted B.C.' (186.2, 9, 8)
## 50% 'Unweighted C.C.' (161.4, 8, 8)
## 80% 'Weights'         (1.0, 0, 7)
## 80% 'Weighted B.C.'   (1.0, 0, 35)
## 80% 'Weighted C.C.'   (41.3, 14, 10)
## 80% 'Unweighted B.C.' (60.6, 14, 13)
## 80% 'Unweighted C.C.' (66.5, 12, 7)
## 90% 'Weights'         (1.0, 0, 3)
## 90% 'Weighted B.C.'   (1.0, 0, 6)
## 90% 'Weighted C.C.'   (17.4, 13, 15)
## 90% 'Unweighted B.C.' (13.5, 20, 23)
## 90% 'Unweighted C.C.' (17.2, 19, 9)

#-# parameters:
#-#  Model                   Barabasi
#-#  Number_of_nodes         300
#-#  Parameter_of_network    20
#-#  Distribution            exponential 0.1
#-#  Immunity_update         2.0 0.8
#-#  N_graph                 5
#-#  N_initial               1
#-#  Number_of_simulations   5
## Averages of all simulations (peak, t_peak, t_half)
## 0% 'Initial'          (229.9, 5, 8)
## 20% 'Weights'         (187.6, 9, 7)
## 20% 'Weighted B.C.'   (182.2, 8, 7)
## 20% 'Weighted C.C.'   (193.9, 6, 7)
## 20% 'Unweighted B.C.' (199.2, 7, 7)
## 20% 'Unweighted C.C.' (221.5, 6, 7)
## 30% 'Weights'         (121.1, 10, 7)
## 30% 'Weighted B.C.'   (163.5, 9, 7)
## 30% 'Weighted C.C.'   (210.6, 8, 6)
## 30% 'Unweighted B.C.' (215.9, 7, 7)
## 30% 'Unweighted C.C.' (191.0, 7, 7)
## 40% 'Weights'         (118.4, 14, 8)
## 40% 'Weighted B.C.'   (95.9, 13, 8)
## 40% 'Weighted C.C.'   (189.6, 7, 8)
## 40% 'Unweighted B.C.' (200.1, 8, 7)
## 40% 'Unweighted C.C.' (195.6, 7, 7)
## 50% 'Weights'         (61.6, 17, 9)
## 50% 'Weighted B.C.'   (69.3, 16, 10)
## 50% 'Weighted C.C.'   (167.8, 8, 8)
```

```
## 50% 'Unweighted B.C.' (173.4, 8, 8)
## 50% 'Unweighted C.C.' (180.8, 8, 7)
## 80% 'Weights'         (1.0, 0, 3)
## 80% 'Weighted B.C.'   (2.5, 18, 24)
## 80% 'Weighted C.C.'   (56.0, 14, 8)
## 80% 'Unweighted B.C.' (47.7, 15, 11)
## 80% 'Unweighted C.C.' (67.5, 14, 7)
## 90% 'Weights'         (1.0, 0, 4)
## 90% 'Weighted B.C.'   (1.0, 0, 15)
## 90% 'Weighted C.C.'   (19.2, 17, 12)
## 90% 'Unweighted B.C.' (9.6, 22, 11)
## 90% 'Unweighted C.C.' (23.8, 20, 13)

#-# parameters:
#-#  Model                    Erdos
#-#  Number_of_nodes          300
#-#  Parameter_of_network     0.125
#-#  Distribution             exponential 0.1
#-#  Immunity_update          2.0 0.8
#-#  N_graph                  5
#-#  N_initial                1
#-#  Number_of_simulations    5
## Averages of all simulations (peak, t_peak, t_half)
## 0% 'Initial'          (241.6, 5, 8)
## 20% 'Weights'         (198.8, 9, 7)
## 20% 'Weighted B.C.'   (193.8, 8, 8)
## 20% 'Weighted C.C.'   (203.9, 7, 7)
## 20% 'Unweighted B.C.' (211.6, 7, 7)
## 20% 'Unweighted C.C.' (233.6, 6, 7)
## 30% 'Weights'         (167.3, 11, 7)
## 30% 'Weighted B.C.'   (156.7, 10, 7)
## 30% 'Weighted C.C.'   (195.8, 7, 7)
## 30% 'Unweighted B.C.' (214.2, 7, 7)
## 30% 'Unweighted C.C.' (195.9, 7, 7)
## 40% 'Weights'         (108.0, 15, 9)
## 40% 'Weighted B.C.'   (131.4, 14, 8)
## 40% 'Weighted C.C.'   (184.8, 8, 7)
## 40% 'Unweighted B.C.' (208.7, 8, 7)
## 40% 'Unweighted C.C.' (199.5, 7, 7)
## 50% 'Weights'         (46.7, 23, 11)
## 50% 'Weighted B.C.'   (79.2, 17, 10)
## 50% 'Weighted C.C.'   (164.0, 8, 8)
## 50% 'Unweighted B.C.' (199.2, 8, 7)
## 50% 'Unweighted C.C.' (174.3, 9, 7)
## 80% 'Weights'         (1.0, 0, 7)
## 80% 'Weighted B.C.'   (1.0, 0, 38)
## 80% 'Weighted C.C.'   (57.2, 12, 7)
## 80% 'Unweighted B.C.' (73.2, 16, 8)
## 80% 'Unweighted C.C.' (76.2, 12, 8)
## 90% 'Weights'         (1.0, 0, 2)
## 90% 'Weighted B.C.'   (1.0, 0, 4)
## 90% 'Weighted C.C.'   (14.5, 16, 12)
## 90% 'Unweighted B.C.' (11.0, 27, 12)
## 90% 'Unweighted C.C.' (13.7, 18, 10)

#-# parameters:
#-#  Model                    Barabasi
#-#  Number_of_nodes          300
#-#  Parameter_of_network     20
#-#  Distribution             exponential 0.1
#-#  Immunity_update          1.5 0.6
#-#  N_graph                  5
#-#  N_initial                1
#-#  Number_of_simulations    5
## Averages of all simulations (peak, t_peak, t_half)
## 0% 'Initial'          (185.4, 6, 8)
## 20% 'Weights'         (114.8, 8, 7)
## 20% 'Weighted B.C.'   (126.6, 8, 7)
## 20% 'Weighted C.C.'   (190.6, 6, 8)
## 20% 'Unweighted B.C.' (183.4, 6, 8)
## 20% 'Unweighted C.C.' (186.9, 6, 8)
## 30% 'Weights'         (99.4, 10, 7)
## 30% 'Weighted B.C.'   (98.0, 9, 7)
```

```
## 30% 'Weighted C.C.'   (170.2, 7, 7)
## 30% 'Unweighted B.C.' (170.5, 7, 7)
## 30% 'Unweighted C.C.' (171.9, 6, 8)
## 40% 'Weights'         (46.3, 14, 7)
## 40% 'Weighted B.C.'   (60.4, 13, 7)
## 40% 'Weighted C.C.'   (156.4, 7, 7)
## 40% 'Unweighted B.C.' (153.6, 8, 6)
## 40% 'Unweighted C.C.' (151.2, 7, 7)
## 50% 'Weights'         (16.2, 19, 9)
## 50% 'Weighted B.C.'   (30.6, 13, 8)
## 50% 'Weighted C.C.'   (130.8, 9, 7)
## 50% 'Unweighted B.C.' (117.7, 9, 7)
## 50% 'Unweighted C.C.' (135.0, 8, 7)
## 80% 'Weights'         (1.0, 0, 3)
## 80% 'Weighted B.C.'   (1.0, 0, 4)
## 80% 'Weighted C.C.'   (22.5, 15, 9)
## 80% 'Unweighted B.C.' (17.4, 13, 13)
## 80% 'Unweighted C.C.' (40.6, 15, 7)
## 90% 'Weights'         (1.0, 0, 2)
## 90% 'Weighted B.C.'   (1.0, 0, 2)
## 90% 'Weighted C.C.'   (2.7, 25, 13)
## 90% 'Unweighted B.C.' (2.1, 14, 10)
## 90% 'Unweighted C.C.' (2.8, 11, 22)

#-# parameters:
#-#  Model                   Erdos
#-#  Number_of_nodes         300
#-#  Parameter_of_network    0.125
#-#  Distribution            exponential 0.1
#-#  Immunity_update         1.5 0.6
#-#  N_graph                 5
#-#  N_initial               1
#-#  Number_of_simulations   5
## Averages of all simulations (peak, t_peak, t_half)
## 0% 'Initial'          (206.4, 5, 8)
## 20% 'Weights'         (150.1, 8, 7)
## 20% 'Weighted B.C.'   (128.7, 9, 7)
## 20% 'Weighted C.C.'   (181.5, 6, 8)
## 20% 'Unweighted B.C.' (174.2, 6, 8)
## 20% 'Unweighted C.C.' (188.9, 6, 8)
## 30% 'Weights'         (90.2, 11, 8)
## 30% 'Weighted B.C.'   (123.0, 12, 5)
## 30% 'Weighted C.C.'   (158.4, 7, 7)
## 30% 'Unweighted B.C.' (180.2, 7, 7)
## 30% 'Unweighted C.C.' (176.0, 7, 7)
## 40% 'Weights'         (42.8, 18, 8)
## 40% 'Weighted B.C.'   (49.4, 17, 9)
## 40% 'Weighted C.C.'   (124.4, 8, 6)
## 40% 'Unweighted B.C.' (160.1, 7, 7)
## 40% 'Unweighted C.C.' (161.5, 7, 7)
## 50% 'Weights'         (11.1, 26, 11)
## 50% 'Weighted B.C.'   (20.4, 21, 11)
## 50% 'Weighted C.C.'   (106.4, 8, 7)
## 50% 'Unweighted B.C.' (140.1, 8, 7)
## 50% 'Unweighted C.C.' (127.5, 8, 7)
## 80% 'Weights'         (1.0, 0, 2)
## 80% 'Weighted B.C.'   (1.0, 0, 5)
## 80% 'Weighted C.C.'   (16.2, 13, 10)
## 80% 'Unweighted B.C.' (36.2, 16, 10)
## 80% 'Unweighted C.C.' (41.6, 12, 8)
## 90% 'Weights'         (1.0, 0, 1)
## 90% 'Weighted B.C.'   (1.0, 0, 2)
## 90% 'Weighted C.C.'   (4.0, 11, 8)
## 90% 'Unweighted B.C.' (1.4, 19, 8)
## 90% 'Unweighted C.C.' (7.0, 17, 11)

#-# parameters:
#-#  Model                   Barabasi
#-#  Number_of_nodes         300
#-#  Parameter_of_network    20
#-#  Distribution            exponential 0.1
#-#  Immunity_update         1.5 0.7
#-#  N_graph                 5
```

```
#-#  N_initial              1
#-#  Number_of_simulations   5
## Averages of all simulations (peak, t_peak, t_half)
## 0% 'Initial'           (218.8, 5, 9)
## 20% 'Weights'          (173.1, 8, 7)
## 20% 'Weighted B.C.'    (148.8, 8, 8)
## 20% 'Weighted C.C.'    (196.2, 6, 8)
## 20% 'Unweighted B.C.' (191.8, 6, 9)
## 20% 'Unweighted C.C.' (193.9, 6, 8)
## 30% 'Weights'          (106.4, 11, 7)
## 30% 'Weighted B.C.'    (120.2, 10, 9)
## 30% 'Weighted C.C.'    (201.6, 7, 8)
## 30% 'Unweighted B.C.' (191.3, 7, 8)
## 30% 'Unweighted C.C.' (187.6, 6, 9)
## 40% 'Weights'          (71.6, 13, 8)
## 40% 'Weighted B.C.'    (78.1, 12, 8)
## 40% 'Weighted C.C.'    (161.2, 8, 8)
## 40% 'Unweighted B.C.' (148.3, 8, 8)
## 40% 'Unweighted C.C.' (177.3, 7, 9)
## 50% 'Weights'          (33.0, 17, 9)
## 50% 'Weighted B.C.'    (54.5, 17, 8)
## 50% 'Weighted C.C.'    (168.4, 8, 8)
## 50% 'Unweighted B.C.' (141.1, 9, 8)
## 50% 'Unweighted C.C.' (114.5, 8, 7)
## 80% 'Weights'          (1.0, 0, 3)
## 80% 'Weighted B.C.'    (1.0, 0, 2)
## 80% 'Weighted C.C.'    (40.4, 14, 8)
## 80% 'Unweighted B.C.' (31.6, 14, 10)
## 80% 'Unweighted C.C.' (41.2, 14, 9)
## 90% 'Weights'          (1.0, 0, 2)
## 90% 'Weighted B.C.'    (1.0, 0, 3)
## 90% 'Weighted C.C.'    (7.7, 17, 13)
## 90% 'Unweighted B.C.' (5.5, 12, 13)
## 90% 'Unweighted C.C.' (16.0, 19, 13)

#-# parameters:
#-#  Model                  Erdos
#-#  Number_of_nodes        300
#-#  Parameter_of_network    0.125
#-#  Distribution           exponential 0.1
#-#  Immunity_update        1.5 0.7
#-#  N_graph                5
#-#  N_initial              1
#-#  Number_of_simulations   5
## Averages of all simulations (peak, t_peak, t_half)
## 0% 'Initial'           (219.1, 5, 10)
## 20% 'Weights'          (164.9, 9, 7)
## 20% 'Weighted B.C.'    (176.1, 8, 8)
## 20% 'Weighted C.C.'    (202.1, 7, 8)
## 20% 'Unweighted B.C.' (201.5, 6, 9)
## 20% 'Unweighted C.C.' (193.0, 6, 9)
## 30% 'Weights'          (124.6, 11, 7)
## 30% 'Weighted B.C.'    (133.0, 12, 6)
## 30% 'Weighted C.C.'    (176.6, 7, 8)
## 30% 'Unweighted B.C.' (205.5, 6, 9)
## 30% 'Unweighted C.C.' (204.7, 6, 9)
## 40% 'Weights'          (69.2, 16, 9)
## 40% 'Weighted B.C.'    (78.2, 15, 9)
## 40% 'Weighted C.C.'    (131.8, 9, 7)
## 40% 'Unweighted B.C.' (178.0, 7, 8)
## 40% 'Unweighted C.C.' (178.0, 8, 7)
## 50% 'Weights'          (24.4, 22, 15)
## 50% 'Weighted B.C.'    (35.6, 24, 10)
## 50% 'Weighted C.C.'    (96.4, 8, 8)
## 50% 'Unweighted B.C.' (176.4, 8, 8)
## 50% 'Unweighted C.C.' (159.2, 8, 8)
## 80% 'Weights'          (1.0, 0, 3)
## 80% 'Weighted B.C.'    (1.0, 0, 7)
## 80% 'Weighted C.C.'    (35.4, 13, 7)
## 80% 'Unweighted B.C.' (43.0, 17, 9)
## 80% 'Unweighted C.C.' (59.0, 13, 7)
## 90% 'Weights'          (1.0, 0, 3)
## 90% 'Weighted B.C.'    (1.0, 0, 4)
```

```
## 90% 'Weighted C.C.'   (11.0, 15, 11)
## 90% 'Unweighted B.C.' (2.0, 32, 11)
## 90% 'Unweighted C.C.' (8.0, 18, 14)

#-# parameters:
#-#  Model                   Barabasi
#-#  Number_of_nodes         300
#-#  Parameter_of_network    20
#-#  Distribution            exponential 0.1
#-#  Immunity_update         1.5 0.8
#-#  N_graph                 5
#-#  N_initial               1
#-#  Number_of_simulations   5
## Averages of all simulations (peak, t_peak, t_half)
## 0% 'Initial'          (241.0, 5, 11)
## 20% 'Weights'         (200.8, 9, 9)
## 20% 'Weighted B.C.'   (186.0, 9, 9)
## 20% 'Weighted C.C.'   (228.7, 6, 10)
## 20% 'Unweighted B.C.' (225.7, 7, 10)
## 20% 'Unweighted C.C.' (227.8, 7, 10)
## 30% 'Weights'         (146.9, 10, 9)
## 30% 'Weighted B.C.'   (149.6, 11, 8)
## 30% 'Weighted C.C.'   (214.2, 7, 10)
## 30% 'Unweighted B.C.' (224.6, 7, 9)
## 30% 'Unweighted C.C.' (210.5, 7, 10)
## 40% 'Weights'         (125.1, 13, 10)
## 40% 'Weighted B.C.'   (121.9, 13, 9)
## 40% 'Weighted C.C.'   (213.3, 8, 9)
## 40% 'Unweighted B.C.' (185.2, 8, 9)
## 40% 'Unweighted C.C.' (209.8, 8, 9)
## 50% 'Weights'         (70.1, 18, 10)
## 50% 'Weighted B.C.'   (74.0, 15, 12)
## 50% 'Weighted C.C.'   (190.0, 9, 9)
## 50% 'Unweighted B.C.' (175.2, 9, 9)
## 50% 'Unweighted C.C.' (187.6, 8, 9)
## 80% 'Weights'         (1.0, 0, 3)
## 80% 'Weighted B.C.'   (4.1, 22, 16)
## 80% 'Weighted C.C.'   (66.0, 15, 10)
## 80% 'Unweighted B.C.' (80.8, 16, 11)
## 80% 'Unweighted C.C.' (85.7, 14, 10)
## 90% 'Weights'         (1.0, 0, 3)
## 90% 'Weighted B.C.'   (1.0, 0, 17)
## 90% 'Weighted C.C.'   (15.5, 18, 12)
## 90% 'Unweighted B.C.' (16.4, 15, 14)
## 90% 'Unweighted C.C.' (21.6, 22, 11)

#-# parameters:
#-#  Model                   Erdos
#-#  Number_of_nodes         300
#-#  Parameter_of_network    0.125
#-#  Distribution            exponential 0.1
#-#  Immunity_update         1.5 0.8
#-#  N_graph                 5
#-#  N_initial               1
#-#  Number_of_simulations   5
## Averages of all simulations (peak, t_peak, t_half)
## 0% 'Initial'          (242.3, 5, 11)
## 20% 'Weights'         (200.4, 9, 9)
## 20% 'Weighted B.C.'   (210.5, 9, 9)
## 20% 'Weighted C.C.'   (225.4, 7, 10)
## 20% 'Unweighted B.C.' (226.0, 6, 10)
## 20% 'Unweighted C.C.' (220.0, 6, 11)
## 30% 'Weights'         (153.0, 12, 8)
## 30% 'Weighted B.C.'   (143.9, 12, 8)
## 30% 'Weighted C.C.'   (215.0, 7, 10)
## 30% 'Unweighted B.C.' (228.0, 7, 10)
## 30% 'Unweighted C.C.' (210.0, 7, 9)
## 40% 'Weights'         (127.6, 15, 9)
## 40% 'Weighted B.C.'   (136.4, 16, 8)
## 40% 'Weighted C.C.'   (176.1, 8, 9)
## 40% 'Unweighted B.C.' (219.8, 8, 9)
## 40% 'Unweighted C.C.' (206.6, 8, 9)
## 50% 'Weights'         (64.3, 23, 10)
```

```
## 50% 'Weighted B.C.'   (75.0, 22, 10)
## 50% 'Weighted C.C.'   (143.2, 10, 8)
## 50% 'Unweighted B.C.' (199.8, 9, 9)
## 50% 'Unweighted C.C.' (150.0, 9, 10)
## 80% 'Weights'         (1.0, 0, 5)
## 80% 'Weighted B.C.'   (1.0, 0, 10)
## 80% 'Weighted C.C.'   (53.4, 14, 9)
## 80% 'Unweighted B.C.' (65.8, 18, 10)
## 80% 'Unweighted C.C.' (82.6, 11, 9)
## 90% 'Weights'         (1.0, 0, 2)
## 90% 'Weighted B.C.'   (1.0, 0, 4)
## 90% 'Weighted C.C.'   (13.0, 19, 10)
## 90% 'Unweighted B.C.' (9.2, 32, 27)
## 90% 'Unweighted C.C.' (25.2, 19, 8)

#-# parameters:
#-#  Model                     Barabasi
#-#  Number_of_nodes           300
#-#  Parameter_of_network      20
#-#  Distribution              exponential 0.1
#-#  Immunity_update           1.5 0.85
#-#  N_graph                   5
#-#  N_initial                 1
#-#  Number_of_simulations     5
## Averages of all simulations (peak, t_peak, t_half)
## 0% 'Initial'          (251.4, 6, 12)
## 20% 'Weights'         (203.2, 9, 11)
## 20% 'Weighted B.C.'   (204.5, 9, 10)
## 20% 'Weighted C.C.'   (225.2, 7, 12)
## 20% 'Unweighted B.C.' (241.6, 7, 12)
## 20% 'Unweighted C.C.' (243.4, 6, 12)
## 30% 'Weights'         (171.1, 11, 10)
## 30% 'Weighted B.C.'   (197.1, 10, 10)
## 30% 'Weighted C.C.'   (237.8, 8, 11)
## 30% 'Unweighted B.C.' (209.1, 9, 10)
## 30% 'Unweighted C.C.' (226.3, 8, 11)
## 40% 'Weights'         (111.0, 16, 10)
## 40% 'Weighted B.C.'   (132.7, 13, 10)
## 40% 'Weighted C.C.'   (208.1, 8, 11)
## 40% 'Unweighted B.C.' (216.9, 8, 11)
## 40% 'Unweighted C.C.' (226.2, 7, 12)
## 50% 'Weights'         (89.9, 20, 14)
## 50% 'Weighted B.C.'   (121.1, 17, 11)
## 50% 'Weighted C.C.'   (190.1, 9, 11)
## 50% 'Unweighted B.C.' (183.2, 10, 11)
## 50% 'Unweighted C.C.' (199.3, 9, 11)
## 80% 'Weights'         (1.0, 0, 14)
## 80% 'Weighted B.C.'   (7.2, 27, 20)
## 80% 'Weighted C.C.'   (61.7, 16, 12)
## 80% 'Unweighted B.C.' (57.0, 15, 19)
## 80% 'Unweighted C.C.' (95.9, 14, 11)
## 90% 'Weights'         (1.0, 0, 5)
## 90% 'Weighted B.C.'   (1.0, 0, 19)
## 90% 'Weighted C.C.'   (30.8, 19, 16)
## 90% 'Unweighted B.C.' (13.6, 15, 16)
## 90% 'Unweighted C.C.' (24.5, 21, 15)

#-# parameters:
#-#  Model                     Erdos
#-#  Number_of_nodes           300
#-#  Parameter_of_network      0.125
#-#  Distribution              exponential 0.1
#-#  Immunity_update           1.5 0.85
#-#  N_graph                   5
#-#  N_initial                 1
#-#  Number_of_simulations     5
## Averages of all simulations (peak, t_peak, t_half)
## 0% 'Initial'          (257.2, 6, 12)
## 20% 'Weights'         (229.9, 9, 10)
## 20% 'Weighted B.C.'   (218.2, 8, 11)
## 20% 'Weighted C.C.'   (223.8, 7, 11)
## 20% 'Unweighted B.C.' (247.1, 7, 11)
## 20% 'Unweighted C.C.' (239.4, 6, 13)
```

```
## 30% 'Weights'         (177.8, 11, 10)
## 30% 'Weighted B.C.'   (212.6, 11, 10)
## 30% 'Weighted C.C.'   (223.1, 7, 11)
## 30% 'Unweighted B.C.' (229.2, 7, 12)
## 30% 'Unweighted C.C.' (233.0, 7, 11)
## 40% 'Weights'         (147.3, 15, 10)
## 40% 'Weighted B.C.'   (161.5, 14, 11)
## 40% 'Weighted C.C.'   (219.4, 9, 10)
## 40% 'Unweighted B.C.' (226.0, 8, 11)
## 40% 'Unweighted C.C.' (205.6, 8, 11)
## 50% 'Weights'         (99.3, 22, 14)
## 50% 'Weighted B.C.'   (104.4, 20, 11)
## 50% 'Weighted C.C.'   (159.4, 10, 10)
## 50% 'Unweighted B.C.' (215.6, 9, 10)
## 50% 'Unweighted C.C.' (185.1, 8, 12)
## 80% 'Weights'         (1.0, 0, 12)
## 80% 'Weighted B.C.'   (3.1, 37, 19)
## 80% 'Weighted C.C.'   (53.2, 15, 9)
## 80% 'Unweighted B.C.' (103.4, 15, 11)
## 80% 'Unweighted C.C.' (109.5, 13, 11)
## 90% 'Weights'         (1.0, 0, 9)
## 90% 'Weighted B.C.'   (1.0, 0, 10)
## 90% 'Weighted C.C.'   (17.0, 18, 10)
## 90% 'Unweighted B.C.' (24.6, 25, 15)
## 90% 'Unweighted C.C.' (45.6, 19, 9)

#-# parameters:
#-#  Model                   Barabasi
#-#  Number_of_nodes         300
#-#  Parameter_of_network    20
#-#  Distribution            exponential 0.1
#-#  Immunity_update         1.5 0.9
#-#  N_graph                 5
#-#  N_initial               1
#-#  Number_of_simulations   5
## Averages of all simulations (peak, t_peak, t_half)
## 0% 'Initial'          (264.8, 6, 15)
## 20% 'Weights'         (216.0, 9, 14)
## 20% 'Weighted B.C.'   (237.3, 10, 13)
## 20% 'Weighted C.C.'   (255.6, 7, 15)
## 20% 'Unweighted B.C.' (260.1, 7, 14)
## 20% 'Unweighted C.C.' (247.6, 7, 15)
## 30% 'Weights'         (209.5, 11, 13)
## 30% 'Weighted B.C.'   (199.1, 11, 14)
## 30% 'Weighted C.C.'   (244.1, 7, 15)
## 30% 'Unweighted B.C.' (247.2, 8, 14)
## 30% 'Unweighted C.C.' (248.4, 8, 14)
## 40% 'Weights'         (175.3, 15, 12)
## 40% 'Weighted B.C.'   (199.2, 13, 13)
## 40% 'Weighted C.C.'   (243.8, 8, 14)
## 40% 'Unweighted B.C.' (230.0, 9, 13)
## 40% 'Unweighted C.C.' (218.0, 7, 14)
## 50% 'Weights'         (137.3, 19, 15)
## 50% 'Weighted B.C.'   (130.0, 16, 14)
## 50% 'Weighted C.C.'   (211.6, 11, 14)
## 50% 'Unweighted B.C.' (215.6, 11, 13)
## 50% 'Unweighted C.C.' (231.6, 9, 13)
## 80% 'Weights'         (1.0, 0, 62)
## 80% 'Weighted B.C.'   (23.6, 42, 22)
## 80% 'Weighted C.C.'   (94.0, 15, 13)
## 80% 'Unweighted B.C.' (100.0, 18, 15)
## 80% 'Unweighted C.C.' (101.6, 16, 13)
## 90% 'Weights'         (1.0, 0, 6)
## 90% 'Weighted B.C.'   (1.0, 0, 8)
## 90% 'Weighted C.C.'   (34.8, 22, 14)
## 90% 'Unweighted B.C.' (27.4, 25, 21)
## 90% 'Unweighted C.C.' (40.1, 25, 16)

#-# parameters:
#-#  Model                   Erdos
#-#  Number_of_nodes         300
#-#  Parameter_of_network    0.125
#-#  Distribution            exponential 0.1
```

```
#-#  Immunity_update         1.5 0.9
#-#  N_graph                 5
#-#  N_initial               1
#-#  Number_of_simulations   5
## Averages of all simulations (peak, t_peak, t_half)
## 0% 'Initial'          (267.8, 6, 15)
## 20% 'Weights'         (246.2, 9, 14)
## 20% 'Weighted B.C.'   (249.0, 9, 13)
## 20% 'Weighted C.C.'   (259.2, 7, 14)
## 20% 'Unweighted B.C.' (262.2, 6, 15)
## 20% 'Unweighted C.C.' (262.5, 6, 15)
## 30% 'Weights'         (221.8, 12, 13)
## 30% 'Weighted B.C.'   (217.4, 11, 13)
## 30% 'Weighted C.C.'   (227.2, 8, 13)
## 30% 'Unweighted B.C.' (237.8, 7, 14)
## 30% 'Unweighted C.C.' (246.9, 7, 15)
## 40% 'Weights'         (199.2, 15, 13)
## 40% 'Weighted B.C.'   (203.1, 15, 13)
## 40% 'Weighted C.C.'   (225.4, 9, 14)
## 40% 'Unweighted B.C.' (232.2, 8, 14)
## 40% 'Unweighted C.C.' (246.5, 8, 14)
## 50% 'Weights'         (161.2, 22, 13)
## 50% 'Weighted B.C.'   (160.2, 20, 13)
## 50% 'Weighted C.C.'   (203.1, 10, 13)
## 50% 'Unweighted B.C.' (243.6, 9, 13)
## 50% 'Unweighted C.C.' (207.5, 10, 12)
## 80% 'Weights'         (1.3, 10, 15)
## 80% 'Weighted B.C.'   (4.8, 46, 24)
## 80% 'Weighted C.C.'   (88.4, 13, 13)
## 80% 'Unweighted B.C.' (104.3, 19, 13)
## 80% 'Unweighted C.C.' (120.1, 14, 15)
## 90% 'Weights'         (1.0, 0, 6)
## 90% 'Weighted B.C.'   (1.4, 46, 11)
## 90% 'Weighted C.C.'   (41.9, 18, 14)
## 90% 'Unweighted B.C.' (38.9, 31, 17)
## 90% 'Unweighted C.C.' (36.8, 17, 15)

#-# parameters:
#-#  Model                   Barabasi
#-#  Number_of_nodes         300
#-#  Parameter_of_network    20
#-#  Distribution            exponential 0.1
#-#  Immunity_update         1.5 0.6
#-#  N_graph                 5
#-#  N_initial               1
#-#  Number_of_simulations   5
## Averages of all simulations (peak, t_peak, t_half)
## 0% 'Initial'          (185.4, 6, 8)
## 20% 'Weights'         (114.8, 8, 7)
## 20% 'Weighted B.C.'   (126.6, 8, 7)
## 20% 'Weighted C.C.'   (190.6, 6, 8)
## 20% 'Unweighted B.C.' (183.4, 6, 8)
## 20% 'Unweighted C.C.' (186.9, 6, 8)
## 30% 'Weights'         (99.4, 10, 7)
## 30% 'Weighted B.C.'   (98.0, 9, 7)
## 30% 'Weighted C.C.'   (170.2, 7, 7)
## 30% 'Unweighted B.C.' (170.5, 7, 7)
## 30% 'Unweighted C.C.' (171.9, 6, 8)
## 40% 'Weights'         (46.3, 14, 7)
## 40% 'Weighted B.C.'   (60.4, 13, 7)
## 40% 'Weighted C.C.'   (156.4, 7, 7)
## 40% 'Unweighted B.C.' (153.6, 8, 6)
## 40% 'Unweighted C.C.' (151.2, 7, 7)
## 50% 'Weights'         (16.2, 19, 9)
## 50% 'Weighted B.C.'   (30.6, 13, 8)
## 50% 'Weighted C.C.'   (130.8, 9, 7)
## 50% 'Unweighted B.C.' (117.7, 9, 7)
## 50% 'Unweighted C.C.' (135.0, 8, 7)
## 80% 'Weights'         (1.0, 0, 3)
## 80% 'Weighted B.C.'   (1.0, 0, 4)
## 80% 'Weighted C.C.'   (22.5, 15, 9)
## 80% 'Unweighted B.C.' (17.4, 13, 13)
## 80% 'Unweighted C.C.' (40.6, 15, 7)
```

```
## 90% 'Weights'         (1.0, 0, 2)
## 90% 'Weighted B.C.'   (1.0, 0, 2)
## 90% 'Weighted C.C.'   (2.7, 25, 13)
## 90% 'Unweighted B.C.' (2.1, 14, 10)
## 90% 'Unweighted C.C.' (2.8, 11, 22)

#-# parameters:
#-#  Model                   Erdos
#-#  Number_of_nodes         300
#-#  Parameter_of_network    0.125
#-#  Distribution            exponential 0.1
#-#  Immunity_update         1.5 0.6
#-#  N_graph                 5
#-#  N_initial               1
#-#  Number_of_simulations   5
## Averages of all simulations (peak, t_peak, t_half)
## 0% 'Initial'          (206.4, 5, 8)
## 20% 'Weights'         (150.1, 8, 7)
## 20% 'Weighted B.C.'   (128.7, 9, 7)
## 20% 'Weighted C.C.'   (181.5, 6, 8)
## 20% 'Unweighted B.C.' (174.2, 6, 8)
## 20% 'Unweighted C.C.' (188.9, 6, 8)
## 30% 'Weights'         (90.2, 11, 8)
## 30% 'Weighted B.C.'   (123.0, 12, 5)
## 30% 'Weighted C.C.'   (158.4, 7, 7)
## 30% 'Unweighted B.C.' (180.2, 7, 7)
## 30% 'Unweighted C.C.' (176.0, 7, 7)
## 40% 'Weights'         (42.8, 18, 8)
## 40% 'Weighted B.C.'   (49.4, 17, 9)
## 40% 'Weighted C.C.'   (124.4, 8, 6)
## 40% 'Unweighted B.C.' (160.1, 7, 7)
## 40% 'Unweighted C.C.' (161.5, 7, 7)
## 50% 'Weights'         (11.1, 26, 11)
## 50% 'Weighted B.C.'   (20.4, 21, 11)
## 50% 'Weighted C.C.'   (106.4, 8, 7)
## 50% 'Unweighted B.C.' (140.1, 8, 7)
## 50% 'Unweighted C.C.' (127.5, 8, 7)
## 80% 'Weights'         (1.0, 0, 2)
## 80% 'Weighted B.C.'   (1.0, 0, 5)
## 80% 'Weighted C.C.'   (16.2, 13, 10)
## 80% 'Unweighted B.C.' (36.2, 16, 10)
## 80% 'Unweighted C.C.' (41.6, 12, 8)
## 90% 'Weights'         (1.0, 0, 1)
## 90% 'Weighted B.C.'   (1.0, 0, 2)
## 90% 'Weighted C.C.'   (4.0, 11, 8)
## 90% 'Unweighted B.C.' (1.4, 19, 8)
## 90% 'Unweighted C.C.' (7.0, 17, 11)

#-# parameters:
#-#  Model                   Barabasi
#-#  Number_of_nodes         300
#-#  Parameter_of_network    20
#-#  Distribution            exponential 0.1
#-#  Immunity_update         1.5 0.7
#-#  N_graph                 5
#-#  N_initial               1
#-#  Number_of_simulations   5
## Averages of all simulations (peak, t_peak, t_half)
## 0% 'Initial'          (218.8, 5, 9)
## 20% 'Weights'         (173.1, 8, 7)
## 20% 'Weighted B.C.'   (148.8, 8, 8)
## 20% 'Weighted C.C.'   (196.2, 6, 8)
## 20% 'Unweighted B.C.' (191.8, 6, 9)
## 20% 'Unweighted C.C.' (193.9, 6, 8)
## 30% 'Weights'         (106.4, 11, 7)
## 30% 'Weighted B.C.'   (120.2, 10, 9)
## 30% 'Weighted C.C.'   (201.6, 7, 8)
## 30% 'Unweighted B.C.' (191.3, 7, 8)
## 30% 'Unweighted C.C.' (187.6, 6, 9)
## 40% 'Weights'         (71.6, 13, 8)
## 40% 'Weighted B.C.'   (78.1, 12, 8)
## 40% 'Weighted C.C.'   (161.2, 8, 8)
## 40% 'Unweighted B.C.' (148.3, 8, 8)
```

```
## 40% 'Unweighted C.C.' (177.3, 7, 9)
## 50% 'Weights'         (33.0, 17, 9)
## 50% 'Weighted B.C.'   (54.5, 17, 8)
## 50% 'Weighted C.C.'   (168.4, 8, 8)
## 50% 'Unweighted B.C.' (141.1, 9, 8)
## 50% 'Unweighted C.C.' (114.5, 8, 7)
## 80% 'Weights'         (1.0, 0, 3)
## 80% 'Weighted B.C.'   (1.0, 0, 2)
## 80% 'Weighted C.C.'   (40.4, 14, 8)
## 80% 'Unweighted B.C.' (31.6, 14, 10)
## 80% 'Unweighted C.C.' (41.2, 14, 9)
## 90% 'Weights'         (1.0, 0, 2)
## 90% 'Weighted B.C.'   (1.0, 0, 3)
## 90% 'Weighted C.C.'   (7.7, 17, 13)
## 90% 'Unweighted B.C.' (5.5, 12, 13)
## 90% 'Unweighted C.C.' (16.0, 19, 13)

#-# parameters:
#-#  Model                    Erdos
#-#  Number_of_nodes          300
#-#  Parameter_of_network     0.125
#-#  Distribution             exponential 0.1
#-#  Immunity_update          1.5 0.7
#-#  N_graph                  5
#-#  N_initial                1
#-#  Number_of_simulations    5
## Averages of all simulations (peak, t_peak, t_half)
## 0% 'Initial'          (219.1, 5, 10)
## 20% 'Weights'         (164.9, 9, 7)
## 20% 'Weighted B.C.'   (176.1, 8, 8)
## 20% 'Weighted C.C.'   (202.1, 7, 8)
## 20% 'Unweighted B.C.' (201.5, 6, 9)
## 20% 'Unweighted C.C.' (193.0, 6, 9)
## 30% 'Weights'         (124.6, 11, 7)
## 30% 'Weighted B.C.'   (133.0, 12, 6)
## 30% 'Weighted C.C.'   (176.6, 7, 8)
## 30% 'Unweighted B.C.' (205.5, 6, 9)
## 30% 'Unweighted C.C.' (204.7, 6, 9)
## 40% 'Weights'         (69.2, 16, 9)
## 40% 'Weighted B.C.'   (78.2, 15, 9)
## 40% 'Weighted C.C.'   (131.8, 9, 7)
## 40% 'Unweighted B.C.' (178.0, 7, 8)
## 40% 'Unweighted C.C.' (178.0, 8, 7)
## 50% 'Weights'         (24.4, 22, 15)
## 50% 'Weighted B.C.'   (35.6, 24, 10)
## 50% 'Weighted C.C.'   (96.4, 8, 8)
## 50% 'Unweighted B.C.' (176.4, 8, 8)
## 50% 'Unweighted C.C.' (159.2, 8, 8)
## 80% 'Weights'         (1.0, 0, 3)
## 80% 'Weighted B.C.'   (1.0, 0, 7)
## 80% 'Weighted C.C.'   (35.4, 13, 7)
## 80% 'Unweighted B.C.' (43.0, 17, 9)
## 80% 'Unweighted C.C.' (59.0, 13, 7)
## 90% 'Weights'         (1.0, 0, 3)
## 90% 'Weighted B.C.'   (1.0, 0, 4)
## 90% 'Weighted C.C.'   (11.0, 15, 11)
## 90% 'Unweighted B.C.' (2.0, 32, 11)
## 90% 'Unweighted C.C.' (8.0, 18, 14)

#-# parameters:
#-#  Model                    Barabasi
#-#  Number_of_nodes          300
#-#  Parameter_of_network     20
#-#  Distribution             exponential 0.1
#-#  Immunity_update          1.5 0.8
#-#  N_graph                  5
#-#  N_initial                1
#-#  Number_of_simulations    5
## Averages of all simulations (peak, t_peak, t_half)
## 0% 'Initial'          (241.0, 5, 11)
## 20% 'Weights'         (200.8, 9, 9)
## 20% 'Weighted B.C.'   (186.0, 9, 9)
## 20% 'Weighted C.C.'   (228.7, 6, 10)
```

```
## 20% 'Unweighted B.C.' (225.7, 7, 10)
## 20% 'Unweighted C.C.' (227.8, 7, 10)
## 30% 'Weights'         (146.9, 10, 9)
## 30% 'Weighted B.C.'   (149.6, 11, 8)
## 30% 'Weighted C.C.'   (214.2, 7, 10)
## 30% 'Unweighted B.C.' (224.6, 7, 9)
## 30% 'Unweighted C.C.' (210.5, 7, 10)
## 40% 'Weights'         (125.1, 13, 10)
## 40% 'Weighted B.C.'   (121.9, 13, 9)
## 40% 'Weighted C.C.'   (213.3, 8, 9)
## 40% 'Unweighted B.C.' (185.2, 8, 9)
## 40% 'Unweighted C.C.' (209.8, 8, 9)
## 50% 'Weights'         (70.1, 18, 10)
## 50% 'Weighted B.C.'   (74.0, 15, 12)
## 50% 'Weighted C.C.'   (190.0, 9, 9)
## 50% 'Unweighted B.C.' (175.2, 9, 9)
## 50% 'Unweighted C.C.' (187.6, 8, 9)
## 80% 'Weights'         (1.0, 0, 3)
## 80% 'Weighted B.C.'   (4.1, 22, 16)
## 80% 'Weighted C.C.'   (66.0, 15, 10)
## 80% 'Unweighted B.C.' (80.8, 16, 11)
## 80% 'Unweighted C.C.' (85.7, 14, 10)
## 90% 'Weights'         (1.0, 0, 3)
## 90% 'Weighted B.C.'   (1.0, 0, 17)
## 90% 'Weighted C.C.'   (15.5, 18, 12)
## 90% 'Unweighted B.C.' (16.4, 15, 14)
## 90% 'Unweighted C.C.' (21.6, 22, 11)

#-# parameters:
#-#  Model                   Erdos
#-#  Number_of_nodes         300
#-#  Parameter_of_network    0.125
#-#  Distribution            exponential 0.1
#-#  Immunity_update         1.5 0.8
#-#  N_graph                 5
#-#  N_initial               1
#-#  Number_of_simulations   5
## Averages of all simulations (peak, t_peak, t_half)
## 0% 'Initial'          (242.3, 5, 11)
## 20% 'Weights'         (200.4, 9, 9)
## 20% 'Weighted B.C.'   (210.5, 9, 9)
## 20% 'Weighted C.C.'   (225.4, 7, 10)
## 20% 'Unweighted B.C.' (226.0, 6, 10)
## 20% 'Unweighted C.C.' (220.0, 6, 11)
## 30% 'Weights'         (153.0, 12, 8)
## 30% 'Weighted B.C.'   (143.9, 12, 8)
## 30% 'Weighted C.C.'   (215.0, 7, 10)
## 30% 'Unweighted B.C.' (228.0, 7, 10)
## 30% 'Unweighted C.C.' (210.0, 7, 9)
## 40% 'Weights'         (127.6, 15, 9)
## 40% 'Weighted B.C.'   (136.4, 16, 8)
## 40% 'Weighted C.C.'   (176.1, 8, 9)
## 40% 'Unweighted B.C.' (219.8, 8, 9)
## 40% 'Unweighted C.C.' (206.6, 8, 9)
## 50% 'Weights'         (64.3, 23, 10)
## 50% 'Weighted B.C.'   (75.0, 22, 10)
## 50% 'Weighted C.C.'   (143.2, 10, 8)
## 50% 'Unweighted B.C.' (199.8, 9, 9)
## 50% 'Unweighted C.C.' (150.0, 9, 10)
## 80% 'Weights'         (1.0, 0, 5)
## 80% 'Weighted B.C.'   (1.0, 0, 10)
## 80% 'Weighted C.C.'   (53.4, 14, 9)
## 80% 'Unweighted B.C.' (65.8, 18, 10)
## 80% 'Unweighted C.C.' (82.6, 11, 9)
## 90% 'Weights'         (1.0, 0, 2)
## 90% 'Weighted B.C.'   (1.0, 0, 4)
## 90% 'Weighted C.C.'   (13.0, 19, 10)
## 90% 'Unweighted B.C.' (9.2, 32, 27)
## 90% 'Unweighted C.C.' (25.2, 19, 8)

#-# parameters:
#-#  Model                   Barabasi
#-#  Number_of_nodes         300
```

```
#-#  Parameter_of_network    20
#-#  Distribution            exponential 0.1
#-#  Immunity_update         1.5 0.85
#-#  N_graph                 5
#-#  N_initial               1
#-#  Number_of_simulations   5
## Averages of all simulations (peak, t_peak, t_half)
## 0% 'Initial'          (251.4, 6, 12)
## 20% 'Weights'         (203.2, 9, 11)
## 20% 'Weighted B.C.'   (204.5, 9, 10)
## 20% 'Weighted C.C.'   (225.2, 7, 12)
## 20% 'Unweighted B.C.' (241.6, 7, 12)
## 20% 'Unweighted C.C.' (243.4, 6, 12)
## 30% 'Weights'         (171.1, 11, 10)
## 30% 'Weighted B.C.'   (197.1, 10, 10)
## 30% 'Weighted C.C.'   (237.8, 8, 11)
## 30% 'Unweighted B.C.' (209.1, 9, 10)
## 30% 'Unweighted C.C.' (226.3, 8, 11)
## 40% 'Weights'         (111.0, 16, 10)
## 40% 'Weighted B.C.'   (132.7, 13, 10)
## 40% 'Weighted C.C.'   (208.1, 8, 11)
## 40% 'Unweighted B.C.' (216.9, 8, 11)
## 40% 'Unweighted C.C.' (226.2, 7, 12)
## 50% 'Weights'         (89.9, 20, 14)
## 50% 'Weighted B.C.'   (121.1, 17, 11)
## 50% 'Weighted C.C.'   (190.1, 9, 11)
## 50% 'Unweighted B.C.' (183.2, 10, 11)
## 50% 'Unweighted C.C.' (199.3, 9, 11)
## 80% 'Weights'         (1.0, 0, 14)
## 80% 'Weighted B.C.'   (7.2, 27, 20)
## 80% 'Weighted C.C.'   (61.7, 16, 12)
## 80% 'Unweighted B.C.' (57.0, 15, 19)
## 80% 'Unweighted C.C.' (95.9, 14, 11)
## 90% 'Weights'         (1.0, 0, 5)
## 90% 'Weighted B.C.'   (1.0, 0, 19)
## 90% 'Weighted C.C.'   (30.8, 19, 16)
## 90% 'Unweighted B.C.' (13.6, 15, 16)
## 90% 'Unweighted C.C.' (24.5, 21, 15)

#-# parameters:
#-#  Model                   Erdos
#-#  Number_of_nodes         300
#-#  Parameter_of_network    0.125
#-#  Distribution            exponential 0.1
#-#  Immunity_update         1.5 0.85
#-#  N_graph                 5
#-#  N_initial               1
#-#  Number_of_simulations   5
## Averages of all simulations (peak, t_peak, t_half)
## 0% 'Initial'          (257.2, 6, 12)
## 20% 'Weights'         (229.9, 9, 10)
## 20% 'Weighted B.C.'   (218.2, 8, 11)
## 20% 'Weighted C.C.'   (223.8, 7, 11)
## 20% 'Unweighted B.C.' (247.1, 7, 11)
## 20% 'Unweighted C.C.' (239.4, 6, 13)
## 30% 'Weights'         (177.8, 11, 10)
## 30% 'Weighted B.C.'   (212.6, 11, 10)
## 30% 'Weighted C.C.'   (223.1, 7, 11)
## 30% 'Unweighted B.C.' (229.2, 7, 12)
## 30% 'Unweighted C.C.' (233.0, 7, 11)
## 40% 'Weights'         (147.3, 15, 10)
## 40% 'Weighted B.C.'   (161.5, 14, 11)
## 40% 'Weighted C.C.'   (219.4, 9, 10)
## 40% 'Unweighted B.C.' (226.0, 8, 11)
## 40% 'Unweighted C.C.' (205.6, 8, 11)
## 50% 'Weights'         (99.3, 22, 14)
## 50% 'Weighted B.C.'   (104.4, 20, 11)
## 50% 'Weighted C.C.'   (159.4, 10, 10)
## 50% 'Unweighted B.C.' (215.6, 9, 10)
## 50% 'Unweighted C.C.' (185.1, 8, 12)
## 80% 'Weights'         (1.0, 0, 12)
## 80% 'Weighted B.C.'   (3.1, 37, 19)
## 80% 'Weighted C.C.'   (53.2, 15, 9)
```

```
## 80% 'Unweighted B.C.' (103.4, 15, 11)
## 80% 'Unweighted C.C.' (109.5, 13, 11)
## 90% 'Weights'         (1.0, 0, 9)
## 90% 'Weighted B.C.'   (1.0, 0, 10)
## 90% 'Weighted C.C.'   (17.0, 18, 10)
## 90% 'Unweighted B.C.' (24.6, 25, 15)
## 90% 'Unweighted C.C.' (45.6, 19, 9)

#-# parameters:
#-#  Model                   Barabasi
#-#  Number_of_nodes         300
#-#  Parameter_of_network    20
#-#  Distribution            exponential 0.1
#-#  Immunity_update         1.5 0.9
#-#  N_graph                 5
#-#  N_initial               1
#-#  Number_of_simulations   5
## Averages of all simulations (peak, t_peak, t_half)
## 0% 'Initial'          (264.8, 6, 15)
## 20% 'Weights'         (216.0, 9, 14)
## 20% 'Weighted B.C.'   (237.3, 10, 13)
## 20% 'Weighted C.C.'   (255.6, 7, 15)
## 20% 'Unweighted B.C.' (260.1, 7, 14)
## 20% 'Unweighted C.C.' (247.6, 7, 15)
## 30% 'Weights'         (209.5, 11, 13)
## 30% 'Weighted B.C.'   (199.1, 11, 14)
## 30% 'Weighted C.C.'   (244.1, 7, 15)
## 30% 'Unweighted B.C.' (247.2, 8, 14)
## 30% 'Unweighted C.C.' (248.4, 8, 14)
## 40% 'Weights'         (175.3, 15, 12)
## 40% 'Weighted B.C.'   (199.2, 13, 13)
## 40% 'Weighted C.C.'   (243.8, 8, 14)
## 40% 'Unweighted B.C.' (230.0, 9, 13)
## 40% 'Unweighted C.C.' (218.0, 7, 14)
## 50% 'Weights'         (137.3, 19, 15)
## 50% 'Weighted B.C.'   (130.0, 16, 14)
## 50% 'Weighted C.C.'   (211.6, 11, 14)
## 50% 'Unweighted B.C.' (215.6, 11, 13)
## 50% 'Unweighted C.C.' (231.6, 9, 13)
## 80% 'Weights'         (1.0, 0, 62)
## 80% 'Weighted B.C.'   (23.6, 42, 22)
## 80% 'Weighted C.C.'   (94.0, 15, 13)
## 80% 'Unweighted B.C.' (100.0, 18, 15)
## 80% 'Unweighted C.C.' (101.6, 16, 13)
## 90% 'Weights'         (1.0, 0, 6)
## 90% 'Weighted B.C.'   (1.0, 0, 8)
## 90% 'Weighted C.C.'   (34.8, 22, 14)
## 90% 'Unweighted B.C.' (27.4, 25, 21)
## 90% 'Unweighted C.C.' (40.1, 25, 16)

#-# parameters:
#-#  Model                   Erdos
#-#  Number_of_nodes         300
#-#  Parameter_of_network    0.125
#-#  Distribution            exponential 0.1
#-#  Immunity_update         1.5 0.9
#-#  N_graph                 5
#-#  N_initial               1
#-#  Number_of_simulations   5
## Averages of all simulations (peak, t_peak, t_half)
## 0% 'Initial'          (267.8, 6, 15)
## 20% 'Weights'         (246.2, 9, 14)
## 20% 'Weighted B.C.'   (249.0, 9, 13)
## 20% 'Weighted C.C.'   (259.2, 7, 14)
## 20% 'Unweighted B.C.' (262.2, 6, 15)
## 20% 'Unweighted C.C.' (262.5, 6, 15)
## 30% 'Weights'         (221.8, 12, 13)
## 30% 'Weighted B.C.'   (217.4, 11, 13)
## 30% 'Weighted C.C.'   (227.2, 8, 13)
## 30% 'Unweighted B.C.' (237.8, 7, 14)
## 30% 'Unweighted C.C.' (246.9, 7, 15)
## 40% 'Weights'         (199.2, 15, 13)
## 40% 'Weighted B.C.'   (203.1, 15, 13)
```

```
## 40% 'Weighted C.C.'   (225.4, 9, 14)
## 40% 'Unweighted B.C.' (232.2, 8, 14)
## 40% 'Unweighted C.C.' (246.5, 8, 14)
## 50% 'Weights'         (161.2, 22, 13)
## 50% 'Weighted B.C.'   (160.2, 20, 13)
## 50% 'Weighted C.C.'   (203.1, 10, 13)
## 50% 'Unweighted B.C.' (243.6, 9, 13)
## 50% 'Unweighted C.C.' (207.5, 10, 12)
## 80% 'Weights'         (1.3, 10, 15)
## 80% 'Weighted B.C.'   (4.8, 46, 24)
## 80% 'Weighted C.C.'   (88.4, 13, 13)
## 80% 'Unweighted B.C.' (104.3, 19, 13)
## 80% 'Unweighted C.C.' (120.1, 14, 15)
## 90% 'Weights'         (1.0, 0, 6)
## 90% 'Weighted B.C.'   (1.4, 46, 11)
## 90% 'Weighted C.C.'   (41.9, 18, 14)
## 90% 'Unweighted B.C.' (38.9, 31, 17)
## 90% 'Unweighted C.C.' (36.8, 17, 15)

#-# parameters:
#-#  Model                  Barabasi
#-#  Number_of_nodes        300
#-#  Parameter_of_network   20
#-#  Distribution           exponential 0.1
#-#  Immunity_update        1.5 0.8
#-#  N_graph                50
#-#  N_initial              1
#-#  Number_of_simulations  5
## Averages of all simulations (peak, t_peak, t_half)
## 0% 'Initial'          (234.7, 6, 10)
## 20% 'Weights'         (201.3, 8, 9)
## 20% 'Weighted B.C.'   (185.9, 8, 10)
## 20% 'Weighted C.C.'   (229.7, 6, 10)
## 20% 'Unweighted B.C.' (217.6, 7, 10)
## 20% 'Unweighted C.C.' (225.8, 6, 10)
## 30% 'Weights'         (156.4, 10, 9)
## 30% 'Weighted B.C.'   (156.9, 10, 10)
## 30% 'Weighted C.C.'   (221.9, 7, 10)
## 30% 'Unweighted B.C.' (211.7, 7, 10)
## 30% 'Unweighted C.C.' (211.1, 7, 10)
## 40% 'Weights'         (119.8, 13, 10)
## 40% 'Weighted B.C.'   (105.4, 13, 10)
## 40% 'Weighted C.C.'   (201.8, 8, 9)
## 40% 'Unweighted B.C.' (197.0, 8, 10)
## 40% 'Unweighted C.C.' (197.8, 7, 10)
## 50% 'Weights'         (68.5, 18, 11)
## 50% 'Weighted B.C.'   (74.7, 16, 11)
## 50% 'Weighted C.C.'   (180.0, 9, 9)
## 50% 'Unweighted B.C.' (170.6, 9, 10)
## 50% 'Unweighted C.C.' (178.8, 8, 10)
## 80% 'Weights'         (1.0, 0, 5)
## 80% 'Weighted B.C.'   (2.7, 21, 18)
## 80% 'Weighted C.C.'   (68.1, 14, 10)
## 80% 'Unweighted B.C.' (56.8, 15, 12)
## 80% 'Unweighted C.C.' (71.7, 14, 9)
## 90% 'Weights'         (1.0, 0, 3)
## 90% 'Weighted B.C.'   (1.0, 0, 6)
## 90% 'Weighted C.C.'   (13.3, 19, 14)
## 90% 'Unweighted B.C.' (8.5, 21, 20)
## 90% 'Unweighted C.C.' (19.0, 21, 13)

#-# parameters:
#-#  Model                  Erdos
#-#  Number_of_nodes        300
#-#  Parameter_of_network   0.125
#-#  Distribution           exponential 0.1
#-#  Immunity_update        1.5 0.8
#-#  N_graph                50
#-#  N_initial              1
#-#  Number_of_simulations  5
## Averages of all simulations (peak, t_peak, t_half)
## 0% 'Initial'          (239.5, 5, 11)
## 20% 'Weights'         (202.1, 9, 9)
```

```
## 20% 'Weighted B.C.'   (202.6, 9, 9)
## 20% 'Weighted C.C.'   (216.8, 7, 10)
## 20% 'Unweighted B.C.' (228.7, 7, 10)
## 20% 'Unweighted C.C.' (231.3, 6, 11)
## 30% 'Weights'         (167.8, 11, 9)
## 30% 'Weighted B.C.'   (166.2, 11, 9)
## 30% 'Weighted C.C.'   (204.2, 7, 10)
## 30% 'Unweighted B.C.' (221.8, 7, 10)
## 30% 'Unweighted C.C.' (221.0, 7, 10)
## 40% 'Weights'         (125.2, 15, 9)
## 40% 'Weighted B.C.'   (123.3, 14, 9)
## 40% 'Weighted C.C.'   (182.9, 8, 10)
## 40% 'Unweighted B.C.' (211.2, 8, 9)
## 40% 'Unweighted C.C.' (203.8, 8, 9)
## 50% 'Weights'         (66.3, 22, 11)
## 50% 'Weighted B.C.'   (68.9, 20, 11)
## 50% 'Weighted C.C.'   (163.1, 9, 9)
## 50% 'Unweighted B.C.' (190.2, 9, 9)
## 50% 'Unweighted C.C.' (182.1, 8, 10)
## 80% 'Weights'         (1.0, 0, 6)
## 80% 'Weighted B.C.'   (1.0, 0, 17)
## 80% 'Weighted C.C.'   (51.5, 13, 9)
## 80% 'Unweighted B.C.' (83.5, 17, 10)
## 80% 'Unweighted C.C.' (74.0, 13, 9)
## 90% 'Weights'         (1.0, 0, 4)
## 90% 'Weighted B.C.'   (1.0, 0, 5)
## 90% 'Weighted C.C.'   (17.0, 16, 12)
## 90% 'Unweighted B.C.' (10.8, 26, 16)
## 90% 'Unweighted C.C.' (23.3, 17, 11)
```
